# The role of personality beliefs and “small talk” in strategic behaviour

**DOI:** 10.1371/journal.pone.0269523

**Published:** 2022-09-02

**Authors:** Neha Bose, Daniel Sgroi

**Affiliations:** 1 Department of Economics, University of Warwick, Coventry, United Kingdom; 2 ESRC CAGE Centre, University of Warwick, Coventry, United Kingdom; 3 IZA, Bonn, Germany; Universidad Carlos III de Madrid Departamento de Matematicas, SPAIN

## Abstract

Humans are predisposed to forming “first impressions” about the people we encounter including impressions about their personality traits. While the relationship between personality and strategic decision-making has been widely explored, we examine the role of *personality impressions* in predicting strategic behaviour and devising behavioural responses. In a laboratory setting, after only 4-minutes of “small talk”, subjects developed a sense of the personality of their partners, particularly extraversion, which consequently changed their behaviour in future interactions. Subjects cooperated more in public goods games when they believed their partner to be extraverted and found it more difficult to out-guess opponents they perceived as similar to themselves in a level-k reasoning task, having engaged in conversation with them. We trace how language can generate these effects using text analysis, showing that talking more makes individuals appear extraverted and pro-social which in turn engenders pro-social behaviour in others.

## 1 Introduction

It is human nature to form “first impressions” or perceptions about the people we meet based on observable verbal and non-verbal behaviours. Social psychologists suggest that the central unit used to understand the behaviour of those around us is closely bound to our perceptions about personality traits [1]. Information about others’ traits plays an integral role when inferring their behaviour in a new setting [2], which in turn can help us prepare our own behavioural response when we interact with them. The implication is that anything that helps us learn about the personality of others can and will change our behaviour towards them in the future.

Personality impressions can be based on a wide variety of elements, such as conversations, manner of speaking, non-verbal actions and physical appearance. Much of the prior literature has focused on personality beliefs formulated from observed physical appearance [3], recorded expressions or behaviour [4] and face-to-face interactions [5]. In our experimental study, we focus on personality beliefs formed in a brief (4-minute) period of “small talk” communication conducted using instant messaging software, together with the ensuing impact of such beliefs on behaviour in later strategic interactions in the laboratory. The emphasis on small talk follows from its ubiquitous role in any social interaction. In a period of negotiation there is often an initial burst of small talk, during a typical working day office workers might chat next to the water cooler or in the office corridor, and appointments with a doctor or financial adviser might begin with pleasantries and a mention of the weather. Opting for instant messaging in the laboratory allows us to omit any confounding effects originating from visual and auditory stimuli. Also, by allowing communication only before the nature of future interactions is known we avoid discussions about future strategies.

Personality theory has become a useful tool in Economics to explain strategic behaviour [6–10]. We hope to expand on the usefulness of personality theory by exploring the impact of impressions about another individual’s personality on subsequent strategic interactions with them. Given our controlled laboratory setting and the brevity of the communication, our analysis focuses on the two broadest and most fundamental personality traits, extraversion and neuroticism [11]. Extraversion and neuroticism, which are associated with positive and negative affect, respectively [11–14] are most likely to be detected in a short bout of interaction due to their pervasive nature. Extraverts, characterised by sociability, warmth, gregariousness and positive emotions [15], stand out in most social settings. On the other hand, the temperamental traits of high emotions, fear, anger and poor inhibition of impulse, associated with neuroticism [11], could also be distinctive in a brief interaction. In line with this literature, our results confirm that beliefs about the other three “Big Five” traits, openness, conscientiousness and agreeableness cannot be accurately detected in our experiment. In fact our results suggest that, of the two fundamental traits, subjects could only form reasonably accurate beliefs about extraversion after a short conversation.

Our research strategy is to consider free-form communication: subjects in our laboratory experiment were not aware that they would eventually face each other in strategic settings, but even if they realised that it was likely they had no inkling of the rules of the games to follow. Nevertheless, in the treatment setting they were given the opportunity to communicate with each other: an opportunity not made available to those in the control setting, who instead produced text in an unrelated placebo task. The advantage of this setting is of course that any variation in behaviour between treatment and control groups must be linked causally to the treatment. A second key feature is the brevity of the communication itself: communication was restricted to a mere 4 minutes: a period of time that is purposefully kept short to reflect the briefest of first impressions or to reflect the kind of small talk that occurs in daily life. The brevity of communication should make the impact of communication all the more remarkable.

Subjects were asked to complete both a standard personality test (the Big Five Inventory or BFI [16] which are extraversion, neuroticism, agreeableness, conscientiousness and openness) as well as an IQ test. They then communicate with a partner for 4 minutes in the treatment, or undertake a placebo task in the control. After this phase they are asked to guess how their partner might have answered the same personality and IQ questions. This guess enabled us to measure the role of a very brief period of communication in developing a cohesive set of beliefs about the personality of their partner. Subjects were also asked to take the “Eyes Test” [17], which served as a measure of the mental modelling of others, otherwise known as “Theory of Mind” [18], which could potentially affect the accuracy of belief formation. The Eyes Test and belief elicitation are incentivised as there are measurable correct answers.

Following belief elicitation, subjects engaged in two archetypal and well-understood games: the two-person public goods game and the 11–20 money request game. The public goods game examines social preferences and free-riding and can also be seen as the simplest possible setting in which there is tension between team-work and individual rationality. The 11–20 money request game [19], on the other hand, is a simple two player game which triggers level-k reasoning [20] and tests cognitive ability in a competitive environment [21]. The public goods game requires players to specify how much they are willing to contribute to a communal pot [22, 23]. While both players benefit from contributions, the individually rational choice is to contribute nothing, hoping to free-ride on the other player’s contributions. The 11–20 game grants players payment equal to their numerical choice but with a high bonus if they pick a number one below that of their rival. The game is normally modelled using level-k reasoning: if level 0 (L0) involves the non-strategic choice of 20, then L1 (defined as the best response to L0) would be to pick 19. More generally LK, best responding to LK-1 necessitates a choice of 20-K, enabling us to infer the cognitive level of a player through their numerical choice. To omit learning effects the experiment is restricted to one-shot games. Just prior to playing these games, players were asked to predict how their partners might play which was again incentivised: giving us an insight into belief formation. In this way we form a direct link from communication to belief formation to behaviour in two distinct settings.

Our results indicate that beliefs about others’ personalities, formed after engaging in small talk with them, can influence decisions made in outcome interdependent games. The impact of personality beliefs on strategic behaviour was significantly more pronounced among the treated subjects who engaged in small talk, compared to the control, who had no information upon which to base predictions about their partner’s personality. However, the manner in which personality beliefs influence decision-making depends on the nature of the game. In the level-k reasoning task, where the objective is to out-think the partner, what matters is the perceived difference between the player and their partner’s personalities, which may be due to the human tendency of anchoring to self-knowledge when inferring the choices of similar others [24]. In particular, the level chosen in the 11–20 money request game is influenced by the perceived similarity (or difference) between the player and their partner’s extraversion. The smaller the perceived difference, the higher the level chosen. This result is consistent with the *perceived similarity hypothesis* [25]. The hypothesis posits that individuals believe that those perceived as similar to themselves will think and act like them when faced with the same situation. When the perceived difference between the player and the partner’s personality is small, the player chooses a higher level, suspecting that the partner will reason likewise and choose a higher level themselves. When the perceived difference between the player and the partner is small it is harder for a player to best respond to the distribution of level-k beliefs, as it becomes harder to out-think the opponent. Note that in the paper we use the terms *opponent* and *partner* interchangeably to refer to the individual the subject was randomly matched with, as the study involved both competitive and cooperative tasks. However, to keep the language neutral, during the experiment the partner or opponent was referred to as ‘the other player’ (see the experiment script in part D of the S1 File).

In contrast, choices in the social preferences game are influenced by the absolute value of the partner’s perceived type. We find that, for players who engage in small talk with their partner, cooperation in the public goods game increases when the partner is believed to be extraverted. This result is in line with the known association of trait extraversion with pro-social behaviours like cooperation [26, 27]. Moreover, *beliefs* about partner’s extraversion has a greater effect on cooperation relative to *own* extraversion, a finding robust to whether we use Ordinary Least Squares (OLS) or 2-stage least squares (2SLS) instrumental variable regression specification.

Since small talk communication is the only means that players have to develop personality beliefs in the study, and the opportunity to communicate is the only difference between the control and treatment groups, we conducted a direct examination of the text used during small talk. We observed that the more talkative partners are believed to be extraverted, consistent with [28], who found that personality judges rated talkative individuals higher on extraversion. While the number of words used is especially helpful as a mechanism for detecting extraverts, providing a reasonably accurate forecast of type, there remains a persistent own-type bias: particularly, extraverts are prone to *complementary self projection bias* making them likely to overstate the extraversion in their partners.

Extraversion is particularly relevant when examining the role of personality beliefs in influencing strategic behaviour. Of all the personality traits, subjects could only form reasonably accurate beliefs about a stranger’s extraversion, after engaging in small talk with them for a brief period. Extraverts, due to their sociability, vigour and outgoing friendliness, are distinctive by nature, making them the most detectable in a brief interaction. Accurate impressions about the other personality dimensions might require future research involving longer interaction times in real-world settings.

Alongside our main contribution on the role of personality beliefs on strategic behaviour, we contribute to research exploring *personality attribution*, by focusing on impressions formed from instant messaging rather than physical appearance or face-to-face interaction [3, 5, 29, 30]. We also add to the existing modest research on the role of small talk which has focused on topics such as building solidarity in work places [31], examining investor sentiment using discussions on stock message boards [32] and improving medical outcomes [33]. Our study instead focuses on the role of small talk on unknown future strategic settings and in particular on the relationship with personality theory which in turn feeds into belief formation. Our focus is therefore on the mechanism that allows unstructured communication to alter behaviour and outcomes that are unknown at the time of communication. Lastly, our study contributes to the literature on strategic sophistication which finds that individuals adjust strategies given the information they have about the opponents [21, 34, 35]. Existing work finds that people adjust strategies based on *exogenous* information provided such as information about the opponent’s cognitive ability [21]. We add to this literature through a novel examination of how individuals adjust their behaviour in the light of *endogenous* belief formation about the opponent’s personality.

We should also contrast the literature on “small talk” with the the large literature on communication with *prior knowledge* of what is to follow [36–39] in which individuals can send messages that relate to future decision-making. In contrast to this “cheap talk” literature, our paper studies how communication between players can affect behaviour when the nature of any future interaction (“rules of the game”) is unknown to the players which makes it harder to incorporate strategic content into communication, forcing our subjects to engage in small talk.

The rest of the paper is structured as follows. Section 2 details the experimental design and the core hypotheses. Section 3 presents the results from the experiment. Section 4 concludes. As the very first study of the interaction between personality beliefs, small talk and strategic behaviour, our work will be necessarily exploratory. Thus, the study can act as a first step before further research: we discuss our results further in a speculative discussion presented in part A of the S1 File.

## 2 Methodology

### 2.1 Experimental design

The experiment was conducted in a laboratory setting with University of Warwick Departmental IRB approval (12–03-2018) and all subjects were required to provide written consent prior to participation.

First, at the onset of the experiment each subject was asked to take the 44-item Big Five Inventory personality test or BFI [16]. The answers to the BFI questionnaire were used to compute an average score for each of the 5 personality traits and the trait scores were then standardised (so each trait distribution had mean 0 and standard deviation 1).

Second, the BFI was followed by an incentivised cognitive ability test, taken from the Raven’s Progressive Matrices test [40], in which subjects were asked to attempt 30 visual puzzles (adapted from [6]). The test was incentivised to motivate cognitive effort required in the task, as is the standard approach within Economics [6, 41].

Third, after the Raven’s test the subjects were asked their beliefs about their own performance in the test which was also incentivised.

Next, each subject was randomly allocated to one of two groups and randomly paired with a partner from the same group as follows:

**Control**. Players were not allowed to communicate with their partners in this condition. Subjects were asked to take part in a placebo task for 4 minutes (full experiment instructions are provided in part D of the S1 File). Then the players were asked their beliefs about their partner’s personality and cognitive abilities. For the former, beliefs were elicited using an 11-item short version of the BFI questionnaire, adapted from [42] and modified to allow subjects to indicate how they felt their partners would answer the questions (the personality belief questionnaire is presented in part E of the S1 File). In essence, players were asked to retake the BFI, albeit a shorter version, but rather than considering how they would answer each question, they were instead asked how their partner would answer. The responses to this task allow us to form a belief in much the same way as we formed implied trait values. The 11-item questionnaire consists of 2 items each for the traits extraversion, conscientiousness, openness and neuroticism and 3 items for the agreeableness trait. An average score was computed for each trait and the trait scores were then standardised.

We could then form personality beliefs directly from the answers they provided. For the latter, subjects were asked how they felt their partner’s performed in the Raven’s task. After answering the questions related to beliefs, subjects were told the rules of the first game. Subjects were then asked for their beliefs about their partner’s strategy, following which, on a separate screen, they were asked for their own decision in the game. After completing the first game they were told the rules of the second game. As with game 1, they were asked their beliefs about the partner’s strategy and their own decision in the game. The partner remained the same for both games. The outcomes of both games were announced at the end of the experiment. Beliefs about the partner’s cognitive abilities and personality, and beliefs about their strategies were incentivised.

**Treatment**. The procedure in the treatment group was the same as the control except, instead of the placebo task, subjects were allowed to electronically communicate with their partners through a chat box on their screens. Note that crucially communication occurred before the nature of future decisions were apparent which makes it difficult to incorporate strategic content specific to the game into communication. Communication time was limited to 4 minutes. Following communication, the players were asked to answer the same belief questions as the control group. After answering the questions, the subjects were told the rules of the first game and asked to play the game. The process was repeated with the second game, as with the control condition.

Subjects were asked to play 2 games, the public goods game and the 11–20 money request game (where each player had the same partner for both games). In the *public goods game* each subject was allocated 20 Experimental Pounds (EP) and, along with their partner, were asked to choose (simultaneously) how much to contribute (*c*_*i*_) to a joint project. *c*_*i*_ was restricted to be an integer between 0 and 20. Payoffs were determined as: $\pi_{i}=\left(20-c_{i}\right)+\frac{3}{4}\left(c_{i}+c_{j}\right)$ where *i* and *j* were the two players. Higher contributions while more costly, were more socially beneficial. Note that the multiplier for cooperation (0.75) is a little higher than the typical 0.4–0.5 in an effort to raise the likelihood of cooperation in our setting since it is not the overall incidence of cooperation we are interested in but rather the differential effect of our treatment, so it was important to ensure a high enough take-up of the opportunity to cooperate. In the public goods game, the selfish equilibrium is 0 and the mutually cooperative response is 20. In the *11–20 money request game* participants were asked to play the basic version of the game [19]. Each player was asked to request an amount of money, an integer between 11 and 20 EP. Each player then received the amount they requested. A player received an additional amount of 20 EP if they asked for exactly one less than their partner. This game has been used to study cognitive hierarchy and in particular level-k thinking. In level-k hierarchy models [43–45] players’ levels or types are heterogeneous but they are assumed to be drawn from the same distribution. Peoples’ beliefs are based on naive initial assessment of others’ likely response called level-0 (or L0) and then beliefs are modified via iterated best response. So level 1 (L1) best responds to L0, L2 to L1 and so on. [19] argue that setting L0 as 20 is the instinctive and salient choice since 20 generates the highest payoff absent any strategic considerations and we follow them in also setting L0 to be 20. This L0 choice implies that a choice of 19 is the L1 choice as it best responds to the L0 strategy and in general the level-X choice is to request 20-X. In the level-k model, the level chosen by a subject is a measure of their strategic sophistication or *type* or rather a measure of the player’s beliefs about the partner’s strategic sophistication or type [34]. The game has no pure Nash equilibrium. The order of the 2 games was randomised across sessions.

Following the two games, subjects were asked to take the *Eyes Test* [17]. For this test, subjects were shown 36 close-up photographs of the eyes and surrounding areas of the face of celebrities and were provided with 4 response options (such as playful, terrified, joking etc.) per photograph. The participants were asked to pick the option which most closely described the mental state of the person in the photograph. Subjects were then asked to answer a list of 30 questions about their risk attitude, the Domain Specific Risk Taking Scale or DOSPERT [46]. Each subject was then asked a series of socio-demographic questions including age, gender and native language.

### 2.2 Logistics

The experiment was conducted between May and November 2018. Subjects were recruited through the SONA online recruitment system at the University of Warwick in the UK. The participants were undergraduate, postgraduate and (non-academic) staff members at the University. The experiment was implemented using Z-tree [47] and pre-registered with the AEA RCT registry [48]. The experiment received ethical approval from Economics Department Internal Ethical Approval Process, University of Warwick. 338 subjects took part in the study, with 170 subjects in the control condition and 168 in the treatment group. Note that we originally recruited around 200 for the treatment and 200 for the control but 2 sessions were removed due to technical errors (which resulted in participants dropping out prior to completion). Out of the 170 control group subjects, 110 subjects played the public goods game first, followed by the 11–20 money request game, and 60 subjects played the games in reverse order. Out of 168 treatment group subjects, 106 played the public goods game first and 62 played the 11–20 money request game first. There were 17 sessions conducted, 20 subjects per session on average. An experimental session lasted for approximately 75 minutes.

The final payoff for subjects in the experiment was made up of several components. Firstly, there was a show-up fee of £4. Second, the players received payoffs based on performance in either the public goods game or 11–20 money request game (chosen randomly). The payoffs for the games were in experimental pounds (EP) with the exchange rate as 5 EP = £1. Third, 2 questions out of the 36 questions of the Eyes Test and 2 puzzles of the 30 puzzles of the Raven’s test were randomly selected with each correct answer accruing a further £1. Lastly, belief questions (about own-cognitive ability, partner’s personality and cognitive ability, and beliefs about partner’s decisions in the 2 tasks) were also incentivised. For the personality beliefs, 1 out of 11 questions was randomly picked and if the answer matched that of the partner then the subject was awarded £1. For the other 4 belief questions, subject was awarded £1 for each correct answer. The socio-demographic questions were not incentivised. The average earnings from the study was £13.20 (including the show-up fee of £4), with a minimum earning of £8.35 and maximum of £18.

### 2.3 Hypotheses

Of the “Big Five” personality traits, the scope of our paper is limited to the two broadest, most fundamental and pervasive traits: extraversion and neuroticism [11]. These two traits were the original “Big Two” personality dimensions [49]. Extraversion and neuroticism, have garnered much attention in the literature owing to their well-established association with positive and negative affect, respectively [11–14] which gives these two traits the greatest chance to be detected in a short bout of communication.

Extraverts by their nature stand out and even in a few minutes it may become clear that you are dealing with someone who is characterised by sociability, gregariousness, assertiveness, warmth, activity and overall positive emotions [15]. On the other hand, the temperamental traits of general emotionality, fearfulness, anger and impulsivity, are associated with the neuroticism trait, and are related to high negative affect [11], which might also be detectable in a brief conversation. These prior observations in the literature make any short communication, such as in our study, more suited to developing reliable beliefs about the partner’s (or the opponent’s) extraversion and neuroticism traits, which can be interpreted by the perceiver as positive and negative vibe given off by the opponent, respectively. However, a brief small talk conversation seems insufficient to form beliefs about the partner’s remaining three Big Five traits. While a brief chat is sufficient to form an overall positive (*extraversion*) or negative (*neuroticism*) view about someone, it is not adequate to convey any usable information about whether the opponent is trusting (an aspect of trait *agreeableness*) or lazy (an aspect of trait *conscientiousness*) or imaginative (an aspect of trait *openness*). Thus, we will limit our hypotheses to the effect of the fundamental personality traits on belief formation and strategic decision making. With respect to beliefs about the opponent’s IQ, we will refrain from formulating any hypotheses given the lack of available literature and where appropriate we will present our results about IQ beliefs as more speculative.

Our experimental setup gives us the following testable hypotheses.

Hypothesis 1: *Personality beliefs about the opponent are not only influenced by the opponent’s true personality measure, but the beliefs are also influenced by the player’s own personality*.

This hypothesis is consistent with the conceptual framework for the impact of social environment on personality proposed by [5], which posits that perceptions (or predictions) about any individual’s personality trait can be influenced by the degree to which the predictor possesses that specific trait themselves. The suggestion in [5] seems particularly true for extraverts who stimulate a positive social environment around them due to their own positivity, making them prone to projecting their extraversion or sociability onto others [5, 50]. For our study, we would only expect to see personality projection in the treatment group since any personality beliefs that appear in the control group must be spurious (given the control group have no information whatsoever upon which to base predictions about their partner’s personality).

Hypothesis 2: *Strategic decision making in outcome interdependent tasks is affected by the individual’s beliefs about the opponent’s personality, an effect which is significantly more pronounced among treatment group subjects who engage in small talk communication*.

We also formulate individual hypotheses about the unique way in which personality beliefs can affect the two different tasks.

Hypothesis 2a: *In the 11–20 money request game, rather than one’s own personality or beliefs about the opponent’s personality, we hypothesise that choices in the game will be influenced by the perceived differences in the pair’s personalities*.

Due to the strategic nature of the 11–20 money request game, the objective of this level-k reasoning game is to correctly gauge the opponent’s choice and then attempt to out-think them. Thus, the game does not solely depend on one’s own type, but success in the game is determined by the ability to out-guess the opponent by assessing their type. Despite the well established link between IQ and level-k reasoning [35], *beliefs* about opponent’s IQ might seem like an unreliable measure of the opponent’s strategic sophistication or type in the limited interaction time available. Beliefs about the opponent’s fundamental personality traits on the other hand can appear as a more reliable measure of the opponent’s type due to the increased likelihood of them being detected through a brief chat. While personality itself lacks any association with level-k reasoning, any difference (or similarity) between the pair’s types (which for our study is personality types) can be interpreted by the player as an indicator of the opponent’s behaviour and thus, in turn, can act as a determinant of own decision making. Consistent with simulation theories of social cognition, individuals tend to anchor on self-knowledge to form mental images about similar others [24]. The *perceived similarity hypothesis* states that the greater the perceived similarity between the individual and their opponent the more likely it is that the individual will believe their opponent to think and act like themselves [25], making perceived similarity or differences a potential contributor to iterative reasoning processes.

Hypothesis 2b: *Players who believe their opponents (or partners) are extraverted, will believe that their opponents will cooperate more and then they in turn will cooperate more themselves*.

This hypothesis seems reasonable given the known association between extraversion and pro-social behaviours like cooperation [26, 27]. This association might encourage the individual to cooperate more, with the hope of mutual cooperation boosting earnings.

Hypothesis 3: *More talkative opponents are believed to be extraverted*.

In this paper, we randomly allocate players either to a treatment in which they engage in small talk with their partners or to a control in which they do not. Since small talk is the only interaction the subjects engage in before eliciting beliefs about the partners’ personalities, it must form the basis for these beliefs. From the player’s perspective the number of words is relatively simple to calculate, arguably easier than say considering the emotional content of words in a very brief conversation. Thus, it is hypothesised that subjects using more words will be rated higher on the extraversion scale as extraverts are usually characterised by their sociability and talkativeness [51, 52]. Further, in a study of personality traits in its natural habitat, personality judges rated talkative participants as more extraverted [28]. We will also evaluate other linguistic features, namely valence, arousal and dominance content of the words spoken by the partner. Valence refers to the pleasantness of a stimulus, arousal is the intensity of emotion provoked by a stimulus, and dominance is the degree of control exerted by a stimulus [53].

Note, while the hypotheses related to personality beliefs (*hypothesis 1*) and the strategic decision making tasks (*hypotheses 2a* and *2b*) were formulated before the experimental trials (based on the pertinent literature cited), the results from the text analysis (*hypothesis 3*) were harder to predict prior to the study owing to the novelty of the setup and were thus more exploratory in nature.

## 3 Results

This section tests our core hypotheses. Part A of the S1 File offers a more in-depth discussion of the key findings of the paper. All regressions reported were run with standardised variables with standard errors clustered at the pair level. The summary statistics of the variables used in the paper are presented in the Table A.2 in the S1 File and the balance tests for the intervention groups are provided in Table A.3 in the S1 File.

### 3.1 Result 1: Personality projection

We begin by looking at the factors that might affect the beliefs which players develop about their partners’ personality traits. The aim is to examine *hypothesis 1* which proposes that beliefs about an individual’s personality depend not only on their true personality traits but are also affected by the predictor’s personality.

Table 1 reports the results of an OLS regression model. The dependent variable is the belief reported by the player about their partner’s level of extraversion and neuroticism. Recall that beliefs are formed in much the same way as underlying values: while personality is assessed using the BFI questionnaire, personality beliefs are elicited using a shorter version of the BFI [42]. For both, average trait scores are calculated and the standardised values are used in the regressions. The independent variables in columns 1 and 3 are the player’s own personality scores, the partner’s true personality scores (as reported by the partner using the BFI), and their interactions with the treatment dummy which equals 1 if the player was in the small talk condition and 0 otherwise. Columns 2 and 4 also control for the subject’s IQ, Eyes Test score, age, a dummy variable for being female, and risk aversion (along with the interactions of the control variables with the treatment dummy). Column 2 shows that in the treatment group, an increase in the *player’s own extraversion* by 1 standard deviation increases the beliefs about *partner’s extraversion* by 0.3 standard deviations more than in the control group (p-value < 0.05). Furthermore, an increase in 1 standard deviation in partner’s true extraversion increases the player’s beliefs about their partner’s extraversion by 0.4 standard deviations more in the treatment group than in the control group (p-value < 0.01). Note that the negative coefficient in the control group for Partner’s extraversion (in columns 1 and 2) is spurious and a statistical artifact driven by noise, since in the control group subjects had no reliable source of information about their partners’ true extraversion. This finding biases the coefficient for Partner’s Extraversion × Treatment upwards. However, the effect of partner’s true extraversion on beliefs developed about the partner’s extraversion remains significant when limiting the analysis just to the treatment group, even after adding the control variables, with coefficient.286 and p-value < 0.01. This coefficient reflects the impact of partner’s true extraversion on extraversion beliefs, as compared to an ‘ideal’ control group with a coefficient of 0 (which of course is impossible to replicate using human subjects).

**Table 1:** Impact of own personality and partner’s true personality on beliefs about partner’s personality.

	Extraversion Belief		Neuroticism Belief	
	(1)	(2)	(3)	(4)
Own Extraversion × Treatment	0.2139*	0.2962**	-0.1105	-0.1241
	(0.117)	(0.125)	(0.117)	(0.130)
Own Neuroticism × Treatment	0.1484	0.1531	-0.0470	-0.0418
	(0.125)	(0.131)	(0.110)	(0.109)
Partner’s Extraversion × Treatment	0.4108***	0.4199***		
	(0.108)	(0.110)		
Partner’s Neuroticism × Treatment			0.0269	-0.0005
			(0.103)	(0.102)
Own Extraversion	0.0209	0.0248	-0.0822	-0.0718
	(0.073)	(0.080)	(0.073)	(0.075)
Own Neuroticism	-0.0075	0.0008	0.0462	0.0600
	(0.085)	(0.087)	(0.083)	(0.080)
Partner’s Extraversion	-0.1280*	-0.1339*		
	(0.070)	(0.075)		
Partner’s Neuroticism			0.0866	0.1069
			(0.071)	(0.070)
Treatment	0.3539***	-0.3127	-0.5100***	-0.1983
	(0.098)	(0.632)	(0.102)	(0.550)
Controls	No	Yes	No	Yes
*N*	338	338	338	338

Standard errors in parentheses. Statistical significance indicated as follows:

* *p* < 0.10,

** *p* < 0.05,

*** *p* < 0.01

The specification for the OLS regressions is:
$$\begin{array}{c} E_{i}\left(pers_{j}\right)=\beta_{1}pers_{i}\times Treat+\beta_{2}pers_{j}\times Treat+\gamma_{1}pers_{i}+\gamma_{2}pers_{j}+\varphi z_{i}\times Treat+\lambda Treat+\omega z_{i}+\epsilon_{i} \end{array}$$ (1)

*pers*_*i*_ is player *i*’s personality, *E*_*i*_(*pers*_*j*_) is player *i*’s beliefs about partner *j*’s personality and *pers*_*j*_ is partner *j*’s true personality. Also, *Treat* is the treatment dummy which equals 1 if the player is in the small talk group and 0 otherwise, *z*_*i*_ are individual characteristics of *i* (i.e. the control variables, namely player *i*’s IQ, Eyes Test score, age, a dummy variable for being female, and risk aversion) and *ε*_*i*_ is an idiosyncratic error term.

**Fig 1:**
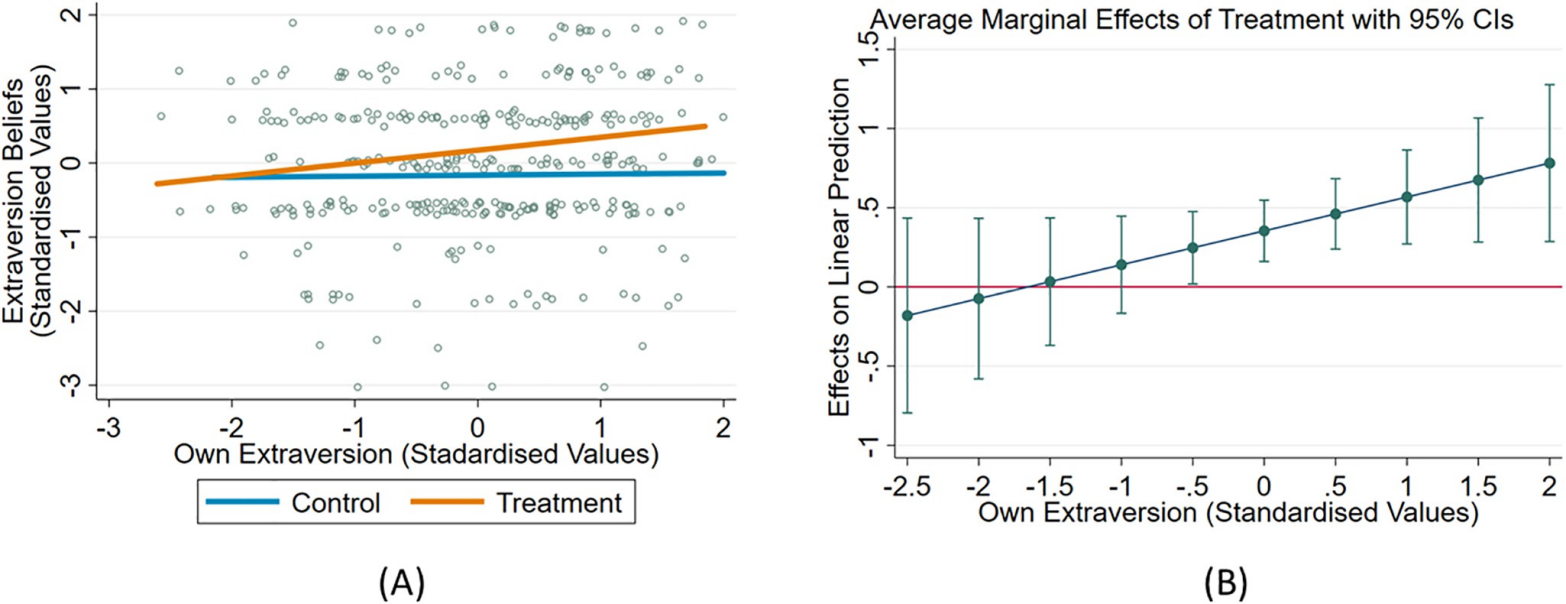
Relationship between the player’s beliefs about partner’s extraversion and the player’s own extraversion score. (A) shows that individuals are more likely to project their own extraversion on to their partners in the Treatment group compared to Control. (B) shows that this difference in extraversion projection between the Treatment and the Control group increases with the value of the predictor’s own extraversion.

Column 4 shows that in the treatment group, an increase in the player’s extraversion by 1 standard deviation decreases the beliefs about partner’s neuroticism by 0.1 standard deviations more than in the control group, although the differential effect is statistically insignificant. Column 4 also shows that a partner’s true neuroticism has no significant effect on beliefs developed about their neuroticism trait. Thus, we find that a 4-minute small talk chat can lead to reliable beliefs about a partner’s extraversion but *not* neuroticism. The relation between own extraversion and beliefs about partner’s extraversion is depicted in Fig 1. Consistent with *hypothesis 1*, we observe that extraverts project their positive affect onto their partners.

For the other 3 Big Five Traits, agreeableness, conscientiousness and openness, the Pearson correlation coefficients between beliefs and true values in the treatment group were trivial and statistically insignificant, with coefficients (r) 0.0372 (p-value = 0.6319), 0.0403 (p-value = 0.6044) and -0.0588 (p-value = 0.4491), respectively. Only for extraversion did we observe significant correlation (r = 0.2513, p-value = 0.0010) between beliefs and true scores in the treatment group, while the coefficient for neuroticism was also insignificant (r = 0.1169, p-value = 0.1314).

We also observed that overestimation of partner’s extraversion increases with the player’s own extraversion (Table A.4 in the S1 File). This overestimation is significantly (p-value < 0.05) more pronounced in the treatment group, compared to the control. Further, we found that with increasing performance in the eyes test, the inaccuracy in the player’s beliefs about partner’s extraversion is significantly (p-value < 0.10) lower in the treatment group compared to the control. This finding is consistent with the literature on the eyes test [17], which posits that better performance in the eyes test indicates increased theory of mind ability, which in turn leads to improved understanding of others’ mental states. With regards to beliefs about partners’ cognitive abilities, it was observed that players project beliefs about their own IQ onto beliefs about partners’ IQ, irrespective of whether they are in the control or treatment group (Table A.5 in the S1 File).

### 3.2 Result 2: Strategic decision-making and personality

Since we divided hypothesis 2 into two parts, each associated with one of our two games, we will also divide our results in the same way.

#### 3.2.1 Result 2a: Level-k reasoning and perceived similarity

**Fig 2:**
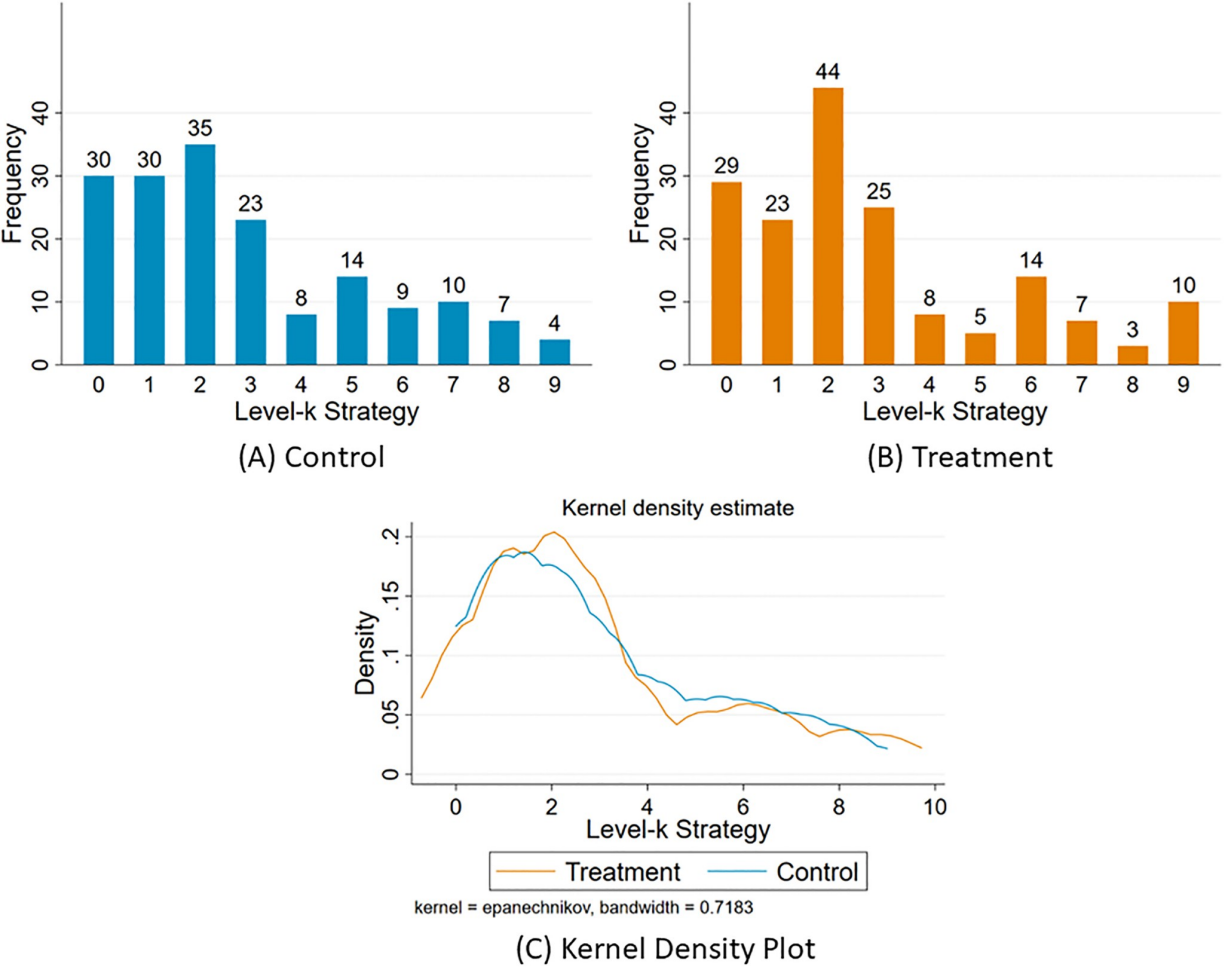
The distribution of level-k strategy chosen in the 11–20 money request game. Note: The level 0 choice in the 11–20 money request game is to request 20, level 1 choice is to request 19 and so on. In general the level-X choice is to request 20-X.

Recall that *hypothesis 2a* claims that level-k reasoning is influenced by the perceived differences (or similarities) in the player and their opponent’s types (which for our study is personality types). In our data, L2 is the most frequently played strategy in both conditions: where 20.6% players choose L2 in the control condition and over 26% do so in the treatment condition (Fig 2). The Kolmogorov-Smirnov test revealed that there is no statistical difference between the distribution of levels of the 2 groups. Further, there is no significant difference between the payoffs earned in the 11–20 game by the control and the treatment group subjects (while the treatment group earns 19.7 EP on average, the control group earns 19.6 EP). Since the level-k game is a competitive game, so long as the communication is two-sided, small talk is unlikely to benefit either player.

Table 2 reports the results of OLS regressions. In columns 1–3 the dependent variable is the player’s beliefs about the level-k strategy chosen by the partner and in columns 4–6 the dependent variable is the level-k strategy chosen by the player. The independent variables are perceived differences between player’s own personality and the partner’s personality, and the interaction of perceived differences with the treatment dummy. The perceived differences are computed by taking the standardised absolute difference between the player’s own personality trait scores and the player’s beliefs about the partner’s personality trait scores. Columns 2 and 4 also include the player’s own personality and the personality measures interacted with the treatment dummy as explanatory variables. Columns 3 and 6 include sensible control variables i.e. player’s eyes test score, IQ, gender, the player’s beliefs about partner’s IQ and the order of play of the two games, which is a dummy that equals 1 when the 11–20 game is played first and 0 when the public goods game is played first (along with the variables interacted with the treatment dummy). Columns 3 and 6 also include the control variables—player’s age and risk aversion, along with their interactions with the treatment dummy. Column 3 shows that an increase in 1 standard deviation in perceived difference in extraversion decreases the player’s beliefs about partner’s level choice by 0.5 more in the treatment group than in the control group (p-value < 0.10). Column 6 shows that an increase in 1 standard deviation in perceived difference in extraversion decreases the player’s own level-k strategy by 0.6 more in the treatment group than in the control group (p-value < 0.05).

**Table 2:** Impact of (absolute) difference between own personality and beliefs about partner’s personality on level-k strategy chosen.

	Level Belief			Level Chosen		
	(1)	(2)	(3)	(4)	(5)	(6)
DiffExtraversion × Treatment	-0.5302*	-0.5562*	-0.5260*	-0.6597***	-0.7373***	-0.6442**
	(0.269)	(0.283)	(0.289)	(0.237)	(0.242)	(0.254)
DiffNeuroticism × Treatment	0.1879	0.2460	0.3734	-0.0415	0.0235	0.1925
	(0.248)	(0.258)	(0.292)	(0.248)	(0.243)	(0.265)
DiffExtraversion	0.1470	0.1430	0.1036	0.2046	0.1792	0.1345
	(0.198)	(0.194)	(0.197)	(0.177)	(0.172)	(0.175)
DiffNeuroticism	-0.1579	-0.1632	-0.2618	-0.1604	-0.1620	-0.2974
	(0.183)	(0.188)	(0.213)	(0.174)	(0.178)	(0.186)
Treatment	0.1668	0.1515	-2.8375	0.0677	0.0330	-2.2355
	(0.267)	(0.268)	(2.058)	(0.279)	(0.276)	(1.860)
Own Extraversion × Treatment		-0.0312	0.0404		-0.1293	0.0116
		(0.294)	(0.344)		(0.290)	(0.312)
Own Neuroticism × Treatment		-0.2018	-0.1717		-0.4371	-0.4405
		(0.279)	(0.306)		(0.278)	(0.279)
Own Extraversion		-0.0532	-0.1518		-0.1726	-0.2696
		(0.195)	(0.201)		(0.211)	(0.212)
Own Neuroticism		0.0132	-0.1102		0.1998	0.0391
		(0.198)	(0.216)		(0.198)	(0.196)
Eyes Test Score × Treatment			0.5507*			0.6041*
			(0.303)			(0.309)
Own IQ × Treatment			-0.2617			-0.2965
			(0.292)			(0.299)
IQ Belief × Treatment			0.3253			0.1933
			(0.311)			(0.264)
Female × Treatment			-0.7230			-0.8284
			(0.611)			(0.555)
Order × Treatment			1.0992*			1.0541*
			(0.576)			(0.592)
Eyes Test Score			-0.4245*			-0.4401*
			(0.247)			(0.248)
Own IQ			0.1777			0.2357
			(0.200)			(0.210)
IQ Belief			-0.3339			-0.3220*
			(0.204)			(0.192)
Female			1.1333***			1.4426***
			(0.431)			(0.384)
Order			-0.7822**			-1.0035**
			(0.392)			(0.408)
Controls	No	No	Yes	No	No	Yes
*N*	338	338	338	338	338	338

Standard errors in parentheses. Statistical significance indicated as follows:

* *p* < 0.10,

** *p* < 0.05,

*** *p* < 0.01

The specification for the OLS regressions is: 
$$\begin{array}{c} Y_{i}=\nu Diffpers_{i}\times Treat+\tau Diffpers_{i}+\eta Treat+\kappa pers_{i}\times Treat+\theta pers_{i}+\rho z_{i}\times Treat+\psi z_{i}+\xi_{i} \end{array}$$ (2)

*Y*_*i*_ is player *i*’s beliefs about partner *j*’s level chosen in the 11–20 game in columns 1–3. For columns 4–6 *Y*_*i*_ is the level chosen by player *i* in the game. *Diffpers*_*i*_ i.e. the absolute difference in *i* and *j*’s personalities as perceived by *i* i.e. |*E*_*i*_(*pers*_*j*_) − *pers*_*i*_| where *pers*_*i*_ is player *i*’s personality, *E*_*i*_(*pers*_*j*_) is player *i*’s beliefs about partner *j*’s personality and *pers*_*j*_ is partner *j*’s true personality. Also, *Treat* is the treatment dummy, *z*_*i*_ are individual characteristics of *i* and *ξ*_*i*_ is an idiosyncratic error term. *z*_*i*_ includes player *i*’s eyes test score, IQ, gender, the *i*’s beliefs about partner *j*’s IQ, the order of play of the two games, which is a dummy that equals 1 when the 11–20 game is played first and 0 when the public goods game is played first and the additional control variables, player *i*’s age and risk aversion.

Thus, there is an inverse relationship between the perceived difference in extraversion between the players, and the player’s level-k strategy, as well as the player’s beliefs about their partner’s level-k strategy choice. Hence, the smaller the perceived difference between the two players the greater the beliefs about partner’s level choice and the greater the level chosen by the player. Note that the results remain similar when we control for beliefs about partner’s personality. The results are omitted here for parsimony but presented in Table A.6 in the S1 File. This result supports *hypothesis 2a* and is consistent with the *perceived similarity hypothesis* which posits that people project their own thinking and decision-making process to predict how their partners might think and act when individuals believe their partners to possess attributes similar to their own [25]. Thus, when players believe their partners to be similar to themselves (small perceived difference), they believe their partners will reason more and choose a higher level (i.e. lower number in the 11–20 game). This logic in turn makes the player choose a higher level. Similar results were not observed for perceived difference between player’s own IQ and partner’s IQ.

Being female enhances beliefs about partner’s level-k choice, as well as player’s own level-k choice, although there is no significant differential treatment effect. Note that [54, 55] have found that women score higher on the social-cognitive element of theory of mind, indicating greater ability to reason about others’ mental states. This result could explain why women choose higher levels.

Further, an increase in the eyes test score by 1 standard deviation increases level belief and level chosen by 0.5 and 0.6 more in the treatment than in the control group, respectively, which supports the finding [21, 34] that greater engagement in theory of mind is associated with superior level-k reasoning, though in this study the effect is significantly (p-value < 0.10) stronger in the treatment group when the players are able to engage in small talk with their partners, compared to the control group. In the control group, order of the tasks has a negative effect on the level-k belief and their own level-k action, whereas in the treatment group the coefficients are positive.

**Fig 3:**
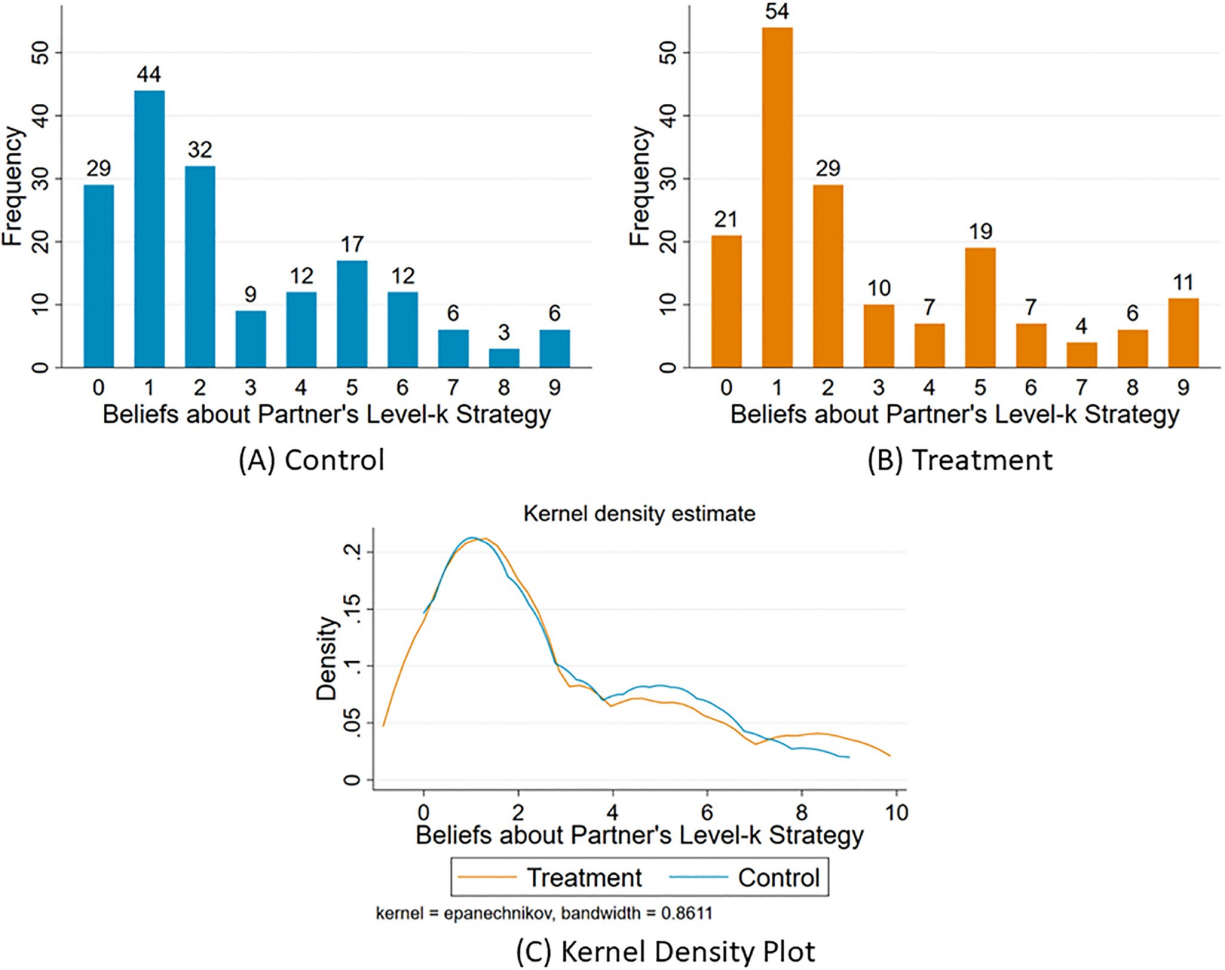
The distribution of the player’s beliefs about partner’s level-k strategy in the 11–20 money request game. The level 0 choice in the 11–20 money request game is to request 20, level 1 choice is to request 19 and so on. In general the level-X choice is to request 20-X.

Next, the paper looks at the distribution of the players’ beliefs about the level-k strategy chosen by their partners (Fig 3). The distribution is presented in Table 3, along with the unique mixed strategy Nash equilibrium distribution for risk-neutral players. The distributions of beliefs observed in both treatment and control groups are different from the equilibrium distribution. In both groups, L1 (i.e choosing 19) is the most frequently believed level-k choice by partners. Table 4 calculates the expected payoffs based on the distribution of level-k beliefs observed. For both control and treatment groups, L2 (choosing 18) has the highest associated expected payoffs. It should be noted that the number of people who best-responded to their own belief about their partner’s level choice i.e. chose to request an amount which was exactly 1 lower than what they believed their partner would choose was 184 out of 334 (94 in the control group and 90 in the treatment group) i.e. 54.4%. The low proportion of people best-responding to their own belief suggests that rather than having an exact belief about their partner’s level choice, they may have formed a distribution of beliefs. The Pearson correlation correlation between a binary variable which takes the value 1 if the subject requested an amount which was exactly 1 lower than what they believed their partner would choose in the 11–20 game and 0 otherwise and the subject’s IQ was 0.1 with p-value = 0.05.

**Table 3:** Distribution of level-k beliefs.

Level	0	1	2	3	4	5	6	7	8	9
Equilibrium (%)	5	10	15	20	25	25				
Treatment (%)	12.50	**32.14**	17.26	5.95	4.17	11.31	4.17	2.38	3.57	6.55
Control (%)	17.06	**25.88**	18.82	5.29	7.06	10.00	7.06	3.53	1.76	3.53

**Table 4:** Expected payoffs from the distribution of level-k beliefs.

Level	0	1	2	3	4	5	6	7	8	9
Treatment (EP)	20.00	21.50	**24.43**	20.45	17.19	15.83	16.26	13.83	12.48	11.71
Control (EP)	20.00	22.41	**23.18**	20.76	17.06	16.41	16.00	14.41	12.71	11.35

Table 5 uses a probit model to examine the effect of perceived differences in the player’s and their partner’s personalities on the probability of best responding to the distribution of level-k beliefs, in the control and treatment groups separately. The dependent variable is the probability of choosing the best response to the distribution of beliefs which is L2 for both control and treatment groups. Column 4 shows that the probability of best responding increases significantly (p-value < 0.01) by 9 percentage points with a 1 standard deviation increase in the perceived difference in extraversion in the treatment group. The effect is negative and insignificant in the control group. Hence, greater the perceived difference in extraversion, higher the chances of best responding by the player in the treatment group. Alternatively, this finding implies that greater the *perceived similarity* between the player and their partner, lower are the chances of the player best responding in the treatment group. This result is consistent with *hypothesis 2a* which supports the perceived similarity hypothesis. When the perceived difference in extraversion is small, the player believes that their partner will act similar to themselves which makes it harder to out-think or out-reason the opponent, thus reducing the probability of best responding. This result holds only when the players engage in small talk as otherwise the players have nothing to base their personality beliefs on and so absent small talk, their beliefs are unlikely to affect decision making.

**Table 5:** Impact of (absolute) difference between own personality and beliefs about partner’s personality on the probability of choosing the best response—Probit model.

	Control		Treatment	
	(1) Pr(Level = 2)	(2) Pr(Level = 2)	(3) Pr(Level = 2)	(4) Pr(Level = 2)
DiffExtraversion	-0.0453	-0.0492	0.0846***	0.0945***
	(0.038)	(0.036)	(0.030)	(0.032)
DiffNeuroticism	-0.0008	-0.0078	-0.0459	-0.0362
	(0.031)	(0.031)	(0.032)	(0.034)
Own Extraversion		0.0115		0.0017
		(0.029)		(0.045)
Own Neuroticism		0.0573*		-0.0399
		(0.032)		(0.037)
Own IQ		0.0655*		0.0566
		(0.035)		(0.039)
IQ Belief		-0.0482*		-0.0070
		(0.029)		(0.035)
Eyes Test Score		0.0541		0.0498
		(0.038)		(0.032)
Controls	No	Yes	No	Yes
*N*	170	170	168	168

Standard errors in parentheses. Statistical significance indicated as follows:

* *p* < 0.10,

** *p* < 0.05,

*** *p* < 0.01

The table reports the average marginal effects from Probit regressions. ‘Controls’ imply the player’s age, gender, risk aversion, and the order of the two games played.

The results hold even after controlling for the player’s IQ and eyes test score, the player’s beliefs about partner’s IQ and other controls—player’s age, gender, risk aversion and the order of games played. In the control group, increase in the player’s IQ by 1 standard deviation increases the probability of best responding by 6 percentage points where as increase in beliefs about the partner’s IQ decreases the probability of best responding by 5 percentage points. The player’s own neuroticism measure also has a significantly (p-value < 0.10) positive effect on the probability of best responding in the control group. Note that the results are robust to the inclusion of personality beliefs as control variables, which are omitted here for parsimony, but are presented in Table A.7 in the S1 File. The results also remain similar when a logit model is used instead of probit as shown in Table A.8 in the S1 File.

**Fig 4:**
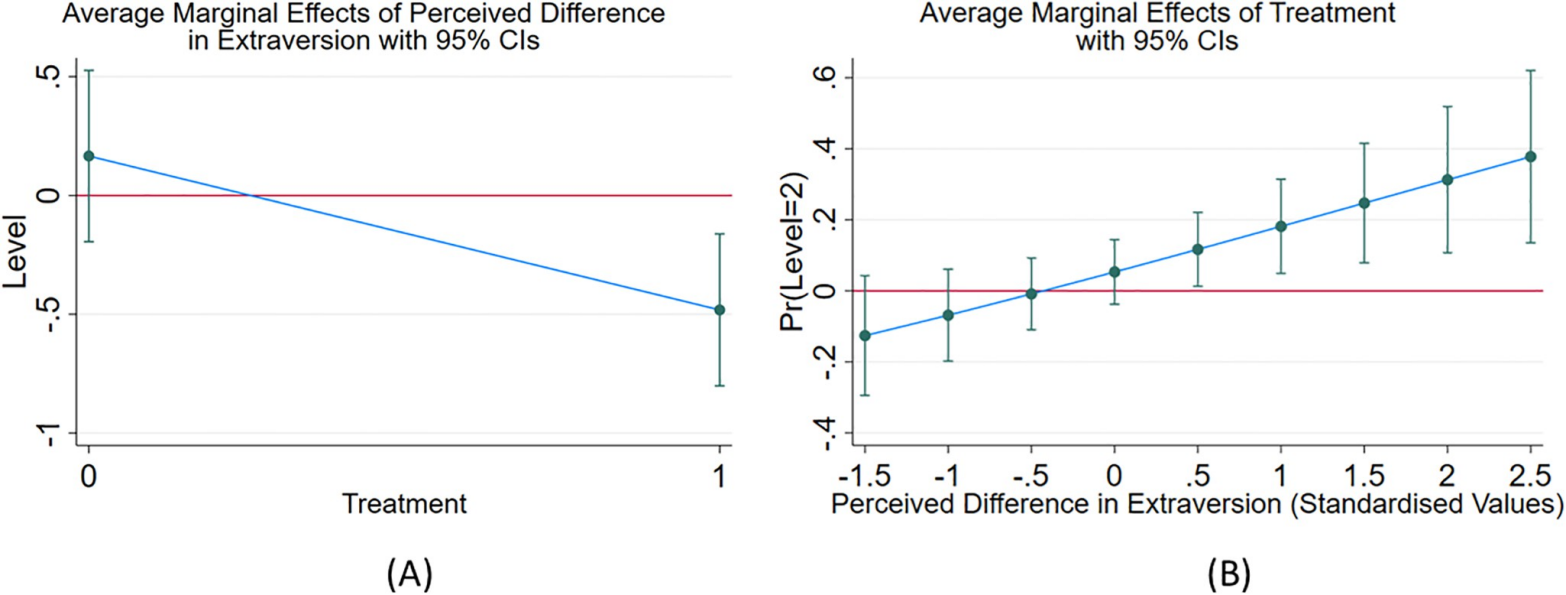
Perceived differences in the players and their partners’ extraversion, and level-k choices made. (A) Effect of perceived difference in extraversion on level choice in control and treatment groups. The figure shows that perceived difference in extraversion has a significant negative effect on the player’s level-k choice in the treatment group. (B) shows that the effect of small talk treatment on probability of best responding to the distribution of level beliefs increases as the perceived difference in extraversion increases.

The relationship between level choice and perceived difference in extraversion is depicted in Fig 4.

#### 3.2.2 Result 2b: Cooperation and extraversion beliefs

Next, we examine the results of the public goods game to test *hypothesis 2b* which states that a player’s cooperation in the game will increase with their beliefs about their opponent’s extraversion, since the player will expect an extraverted opponent to cooperate more. Of the two fundamental personality traits, we expect extraversion to be especially relevant for the public goods game, since it is extraversion that is most associated with pro-social behaviours [26, 27]. We also see from Table A.9 in the S1 File that beliefs about partner’s neuroticism has no significant effect on decision making in the public goods game.

**Fig 5:**
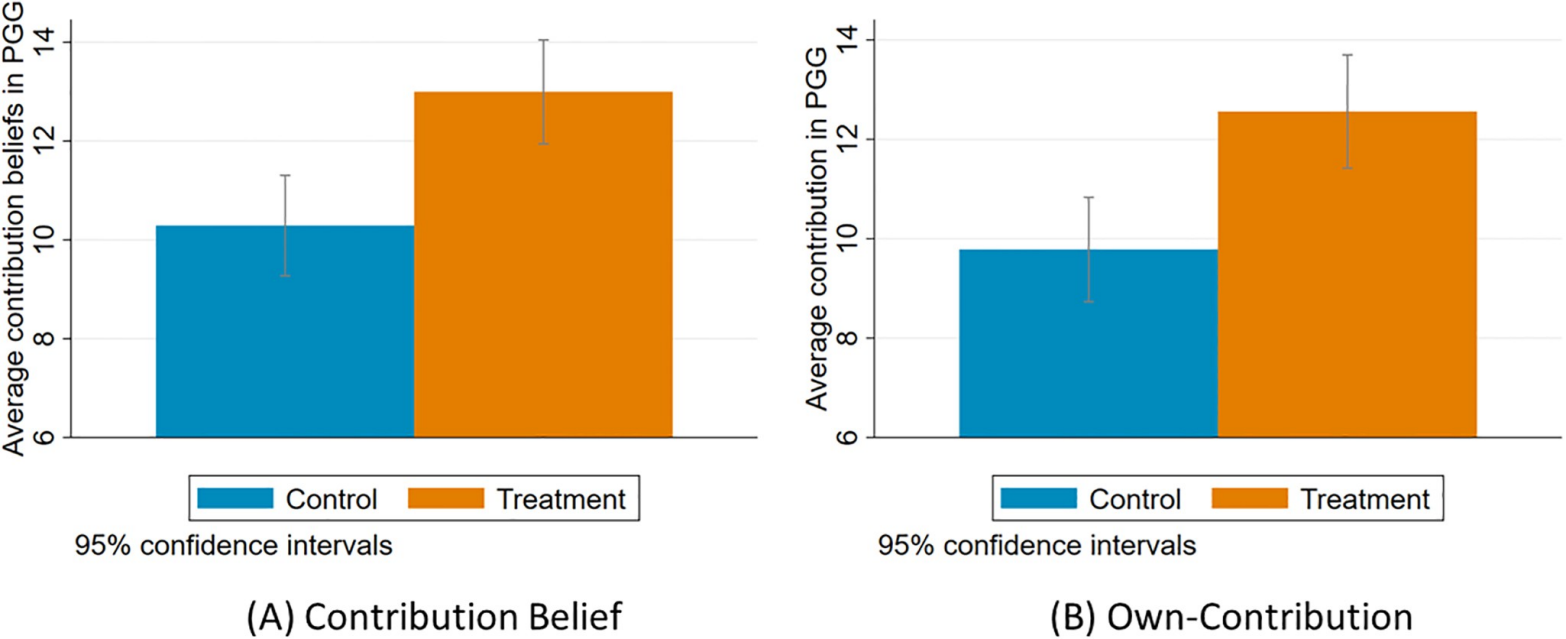
(A) Average Beliefs about Partner’s Contribution and (B) Average Contribution in the Public Goods Game.

In the public goods game, the average beliefs about partner’s contribution in the treatment group was 13 experimental pounds (EP), where as in the control group it was 10.3 EP. This difference is statistically significant with p-value < 0.01 and a t-statistic of -3.640. The average contribution in the treatment group was 12.6 EP, whereas in the control group it was 9.8 EP. This difference is statistically significant with p-value < 0.01 and a t-statistic of -3.525 (Fig 5). The Kolmogorov-Smirnov tests for equality of distributions of own contribution as well as beliefs about partner’s contribution between the treatment and control groups were rejected with p-value < 0.01 for both. This finding is consistent with the existing literature which finds that pre-game communication of any form increases cooperation rates [37, 39].

Our analysis for the public goods game will only consider the observations in which the subjects played the public goods game before the level-k reasoning game. The rationale is that playing the level-k game first seems to trigger level-k reasoning [34], thus biasing decision-making in the social preferences task. On the other hand, since the level-k game strictly requires level-k reasoning, without invoking any social preferences (a point made explicitly in [19]), the results of the 11–20 game are not biased by playing the public goods game first. Further, treated subjects contribute significantly more on average compared to control group subjects, only when the public goods game is played first, where as the difference is insignificant when the public goods game is played second (Figure A.2 in the S1 File). The results from the public goods game, for those who played the 11–20 game first are presented in Figure A.3 and Table A.12 in the S1 File. Further, the results from the public goods game for both orders of play combined are provided in Table A.13 in the S1 File.

We examine *hypothesis 2b* using Eq 3. *Choice*_*i*_ is player *i*’s choice (or contribution) in the public goods game, *pers*_*i*_ is player *i*’s personality, *E*_*i*_(*pers*_*j*_) is player *i*’s beliefs about partner *j*’s personality, *z*_*i*_ are individual characteristics of *i* and *ε*_*i*_ is an idiosyncratic error term.

$$\begin{array}{c} Choice_{i}=\beta_{1}pers_{i}+\beta_{2}E_{i}\left(pers_{j}\right)+\gamma z_{i}+\varepsilon_{i} \end{array}$$ (3)

$$\begin{array}{c} E_{i}\left(pers_{j}\right)=\lambda_{1}pers_{j}+\lambda_{2}pers_{i}+\rho z_{i}+\epsilon_{i} \end{array}$$ (4)

Players’ tendency to project their own extraversion onto their partners creates an endogeneity issue (result 1), and as such estimation of Eq 3 requires valid instruments. Beliefs about partner’s extraversion depend on two components—the player’s own extraversion and the partner’s true extraversion, as discussed in section 3.1. These two components are independent as the two players are randomly matched. Therefore, beliefs about partner’s extraversion can be instrumented with the partner’s true extraversion. Eq 4 is the first stage. *pers*_*j*_ is the partner *j*’s true personality.

The first stage results presented in Table 6 show that partner’s true extraversion significantly enhances beliefs about partner’s extraversion in the treatment, but not in the control group, since in the control group the player has no interaction with their partner. To test for weak instruments, a Wald test is conducted, which tests the null that the coefficients of the endogenous regressors are zero. The null for the treatment group, is rejected at the 5% level. This finding suggests that weak instruments are not an issue here. Further, the F-statistic in the first stage regression (for two-stage least squares) is greater than 10, which indicates that the instruments are strong [56] for the treatment group.

**Table 6:** First stage: Extraversion beliefs and public goods game.

	Control		Treatment	
	(1) Extraversion Belief	(2) Extraversion Belief	(3) Extraversion Belief	(4) Extraversion Belief
Own Extraversion	0.0299	0.0333	0.2147**	0.2614**
	(0.086)	(0.102)	(0.106)	(0.103)
Partner’s Extraversion	-0.1015	-0.0977	0.3541***	0.3648***
	(0.081)	(0.092)	(0.093)	(0.094)
Own IQ		-0.1034		0.0121
		(0.103)		(0.102)
IQ Belief		-0.0559		0.0166
		(0.147)		(0.095)
Eyes Test Score		-0.0470		0.1195
		(0.107)		(0.073)
Controls	No	Yes	No	Yes
*N*	110	110	106	106

Standard errors in parentheses. Statistical significance indicated as follows:

* *p* < 0.10,

** *p* < 0.05,

*** *p* < 0.01

‘Controls’ refers to the player’s age, gender and risk aversion.

Table 7 presents the results of a two-stage least squares instrumental variable (IV) regression for the treatment group. Since the endogeneity bias only exists for the treatment group, Eq 3 is estimated without an instrumental variable for the control group, and is presented in columns 1 and 2 of Table 7.

**Table 7:** Impact of beliefs about partner’s personality and own personality on beliefs about partner’s contribution and own contribution in public goods game.

	Control OLS		Treatment IV	
	(1) Contribution Belief	(2) Own Contribution	(3) Contribution Belief	(4) Own Contribution
Extraversion Belief	0.0601	0.1110	0.6091**	0.5184**
	(0.082)	(0.092)	(0.264)	(0.262)
Own Extraversion	-0.0733	-0.2041**	-0.3074**	-0.2018
	(0.095)	(0.088)	(0.134)	(0.138)
Own IQ	-0.0583	-0.0417	0.0856	0.1548
	(0.096)	(0.084)	(0.094)	(0.103)
IQ Belief	0.1250	0.1140	0.0871	0.2402***
	(0.091)	(0.100)	(0.086)	(0.088)
Eyes Test Score	-0.0431	-0.0015	0.1043	0.1502
	(0.096)	(0.118)	(0.117)	(0.139)
Controls	Yes	Yes	Yes	Yes
*N*	110	110	106	106

Standard errors in parentheses. Statistical significance indicated as follows:

* *p* < 0.10,

** *p* < 0.05,

*** *p* < 0.01

‘Controls’ refers to the player’s age, gender and risk aversion.

Columns 3 and 4 of Table 7 show that in the treatment group, an increase in 1 standard deviation in extraversion belief, increases beliefs about partner’s contribution and own-contribution by 0.6 and 0.5 standard deviations, respectively (p-value < 0.05 for both). On the other hand, an increase in 1 standard deviation in own-extraversion decreases beliefs about partner’s contribution, as well as the player’s own-contribution by 0.3 (p-value < 0.05) and 0.2 (insignificant) standard deviations, respectively. Thus, beliefs about partner’s extraversion has a positive and relatively larger effect, compared to own-extraversion, on decision-making in the public goods game in the treatment group. For the control group, column 2 shows that the player’s extraversion significantly (p-value < 0.05) and negatively impacts contribution level. Beliefs about partner’s extraversion has no significant effect on both beliefs about partner’s contribution and own-contribution in the control group (which makes perfect sense since in the control group, where there is no interaction, players have no basis upon which to form sensible beliefs about their partners). Columns 3 and 4 can essentially be summarised as showing that there are two forces at work in determining how the contribution level is affected by extraversion: a direct and negative effect of own-extraversion, and an indirect and positive effect that works through beliefs about the partner’s extraversion. Overall, the role of beliefs seems stronger than own-extraversion though both are important. Estimating Eq 3 for the treatment group using OLS, and not an IV approach, yields similar results where, in the treatment group, beliefs about partner’s extraversion has a significant positive effect on both beliefs about partner’s contribution as well as own contribution in the public goods game and own-extraversion has an insignificant negative impact on both (Table A.10 in the S1 File). However, given the scope for endogeneity bias, the IV approach is likely to be more appropriate.

Moreover, consistent with *hypothesis 2b*, we find that players cooperate more in the public goods game when they believe their partners to be extraverted.

Following [57], we divide extraversion of the player into 2 facets, assertiveness and activity. This is in line with [57] who propose forming 10 facet scores, 2 for each of the Big Five traits, by dividing the 44 items in the BFI questionnaire. Assertiveness and activity facet scores are formed for each individual based on their responses to specific items in the BFI. This division is carried out to examine which particular facet of extraversion is responsible for driving cooperation decisions. While assertiveness can be defined as preference for exerting control in a group setting [58], activity (or enthusiasm) describes both positive emotions and outgoing friendliness or sociability [59]. The facet analysis (Table A.11 in the S1 File) revealed that of the 2 facets of extraversion, it is facet assertiveness which is responsible for the negative effect of the player’s extraversion on beliefs about partner’s contribution, as well as own contribution in the public goods game.

## 4 Conclusion

The link between personality and strategic behaviour has garnered much attention in recent Economic literature. We expand on this relationship by providing evidence of the impact of impressions about others’ personalities on subsequent strategic interactions with them. In a laboratory setting we show that, when subjects engage in brief small talk interaction with strangers via an instant messaging software, they develop beliefs about the stranger’s personality traits, particularly extraversion, which affect their ensuing strategic behaviour. Extraverts, who are characterised by sociability and gregariousness, tend to be distinctive by nature, making extraversion the most detectable trait in a short bout of communication. Perceptions of trait extraversion, thus, played a crucial role in two well-known strategic decision making tasks—the 11–20 money request game which examines level-k reasoning and the public goods game which is a game of cooperation. Analysis of the pre-game interaction revealed that subjects use the number of words spoken as a a mechanism for detecting extraverts, which does indeed provide a reasonably accurate forecast of type. However, perceptions about extraversion can be coloured by *complementary self projection bias* which makes extraverts prone to projecting their extraversion or positive affect onto those they interact with. Indeed this self-projection bias could partially explain the extent to which we observed a significant effect of perceived differences in players’ personalities on decision-making rather than actual differences in their personalities. This has the effect of limiting the extent to which we can use our results to predict behaviour on the basis of true underlying personality (other than perceived personality). This could potentially be an interesting area for future research that goes beyond “small talk” and into long-run repeated interaction which might engender more accurate personality beliefs which could in turn feed into behaviour. More generally, we hope that this study paves the way for future research exploring the association between personality impressions and strategic behaviour in a variety of tasks and real world contexts.

## Supporting information

S1 File Online supporting materials. (PDF)

S2 File (ZIP)
