# Supplementary material for: The role of personality beliefs and “small talk” in strategic behaviour
Source: PLoS One. 2022 Sep 2;17(9):e0269523. doi: 10.1371/journal.pone.0269523 (PMC9438804; doi:10.1371/journal.pone.0269523)
Supplement: S1 File — (PDF) [file pone.0269523.s001.pdf]

Supplementary Information for “The Role of Personality Beliefs and “Small Talk” in Strategic Behaviour”

Neha Bose[1], Daniel Sgroi[1,2,3*]

1 Department of Economics, University of Warwick, Coventry, UK
2 ESRC CAGE Centre, University of Warwick, Coventry, UK
3 IZA, Bonn, Germany

* Daniel.Sgroi@warwick.ac.uk

Abstract

This document provides additional material related to “The Role of Personality Beliefs and “Small Talk” in Strategic Behaviour” including an extended discussion of the results, additional tables and figures, and the full experimental script.

A Discussion

While the association between an individual’s personality and strategic behaviour has been well established within Economics [1–5], what remains unexplored is the impact of perceptions about another individual’s personality and how these perceptions might influence subsequent strategic interactions. We examine this potential impact by providing subjects the opportunity to develop beliefs about the fundamental personality traits of their partner in a controlled laboratory setting, through a brief (4-minute) chat via their computer screens. We label this type of communication as *small talk* since there is no prior knowledge of the definitive rules of any future strategic interaction between the pair and also based on our observations of the nature of the communication. Following the short period of small talk and subsequent personality belief elicitation, the pair engage in two well-known one-shot strategic decision making tasks: the 11-20 money request game [6], which examines level-k choices, and the public goods game, which is a game of cooperation. We examine the causal effect of beliefs about the partner’s personality on decisions made in the two tasks through comparison with a control group, where subjects participated in an independent placebo task instead of engaging in small talk.

An examination of personality beliefs in section 3.1 of the main paper revealed that beliefs about an individual’s extraversion are not only determined by their true extraversion trait, but are also enhanced by the predictor’s extraversion: extraverts tend to believe that their partners are also extraverted. This finding lends support to our first hypothesis which states that while formulating beliefs about someone’s personality, individuals tend to project their own traits. This effect is significantly stronger in the treatment group than in the control group. This finding also links closely with the psychological literature on extraversion: an extraverted person, who is subject to positive emotions, fosters a positive social environment around them and projects their extraversion or sociability onto others [7, 8], making them prone to *complementary self projection bias.* No such projection was observed for neuroticism.[1] We also found that

[1]This finding on neuroticism is contradictory to the theory of *neurotic projection* which is a form of defence mechanism through which people tend to project negative feelings, motives or behaviour they might possess and are uncomfortable with, onto others. Our results could be attributed to the negative

personality beliefs developed about a partner, after engaging in small talk with them, were only a reliable or accurate measure of the partner's extraversion, but not their neuroticism. This result is consistent with the findings of [7] who also observed accurate perceptions about a stranger's extraversion after a 5-minute in-person face-to-face conversation.

In section 3.2.1 *result 2a* in the main paper, we observed that the perceived similarity or difference between the personalities of the players and their partners influenced decision making in level-k reasoning games, consistent with *hypothesis 2a*. Particularly, perceived differences in the pair's extraversion traits inversely affect the player's level-k choice, an effect which is significantly stronger in the treatment group, compared to the control. In level-k reasoning games a player's strategy reflects the player's beliefs about the opponent's type. The player best responds to these beliefs, attempting to out-reason or out-think their opponent. In accordance with simulation-based theories of mental modelling, perceptions anchor onto own-reasoning processes and likely choices and are then adjusted for any discrepancy between self and other, while inferring choices of similar others [9]. Thus, in level-k games, the perceived similarity or differences between the type of player and their partner, play a crucial role in predicting how the opponent might behave and in turn determine own strategy choice. The *perceived similarity hypothesis* [10] states that when a player thinks they are faced by a similar opponent, they believe the opponent will reason and act in ways similar to themselves. Thus, when the player assumes the partner's type is similar to their own, it becomes harder for them to out-reason the partner in the level-k game. When faced by a similar other, player believes that the opponent, undergoing the same thinking process, will reason harder and pick a higher level which in turn should make the player choose a higher level as well. Consequently, when the player suspects their partner's type is similar to their own, the probability of them best responding to the distribution of level-k beliefs falls. This result holds only when the players engage in small talk as in the control condition the player has no reliable indicator of perceived similarity with the opponent.

In section 3.2.2 *result 2b* in the main paper, we found that when a player thinks that their opponent is extraverted, they believe that their opponent will cooperate more, a result only observed in the small talk treatment. The result that extraverts are expected to cooperate more in social situations, is consistent with the finding in psychology that higher levels of the extraversion trait are associated with pro-social behaviour [11, 12]. Thus, the player themselves cooperate, expecting cooperation from their opponent. In contrast, the literature is conflicted on the effect of a subject's own extraversion on cooperation. While [5] and [13] find a positive effect of extraversion on cooperation, [14] find the opposite. [5] posit that individuals who score highly on the enthusiasm facet of extraversion, owing to their positive outlook, view cooperation as rewarding and expect cooperative behaviour from their partners as well. The opposing argument is that introverts, and not extraverts, are likely to cooperate more as they are more inclined to avoid conflicts [14]. This paper supports the latter argument. We would also argue that some of the contradictions seen in the literature stem from missing the subtle interactions with beliefs that are highlighted in our results. Further, this negative effect of extraversion is driven by the assertive facet of an extravert's personality. Lastly, beliefs about opponent's extraversion have a relatively larger effect on decision-making in the public goods game than own-extraversion. Since these effects work in opposite directions they may partly explain the apparent contradictions seen in the general literature on extraversion and cooperation since they only become apparent when we disentangle the impact of beliefs and own-characteristics.

Consistent with *hypothesis 2*, we show that beliefs about a partner's personality -

---

connotations of the trait neuroticism. Individuals are less keen to project trait neuroticism as it is likely to draw attention to their own neuroticism.

specifically beliefs about partner's extraversion - developed after engaging in small talk, significantly impact choices made in subsequent strategic interactions. The reason why extraversion plays a big role in our study is likely because, out of the two fundamental personality traits, subjects could only form reasonably accurate beliefs about the partner's extraversion. Extraverts, characterised by their sociability, enthusiasm and gregariousness, tend to stand out by nature, making extraversion the most detectable trait, especially after a brief chat. Extraversion, as one of the principal dimensions of personality, can explain a wide variety of outcomes, such as subjective well-being measures [15], health outcomes [16], relationship satisfaction [17] and occupational choices [18]. These features of extraversion may explain why beliefs about the extraversion of a partner is crucial for explaining strategic behaviour in our study.

The brief period of small talk, as the key experimental manipulation in our study, was the only opportunity for the players to interact and hence the primary basis for developing personality beliefs. We might ask how such a brief period of communication could change beliefs about personality. One way to consider this question would to examine the text data present in the small talk directly. In particular, in line with *hypothesis 3* we will focus on the effect of the number of words spoken by the partner on beliefs to check whether more talkative players are indeed believed to be extraverted. The number of words is perhaps the easiest language characteristic to calculate. We also examine the scores for three affective or emotional components of the partner's language use, namely *valence*, *arousal* and *dominance*, using the score-ratings proposed by [19]. The valence rating of a word refers to the pleasant emotion conveyed by a word, with the rating increasing as it moves from unhappy to happy. Arousal rating of a word increases with the degree of excitement emoted by it. Finally, the dominance rating of a word increases with the degree to which it conveys the emotion of *being in control*.[2]

Table A.1 reports the results for the quantitative language characteristics that we consider. The dependant variables are beliefs about the partner's fundamental personality traits. Column 1 shows that beliefs about partner's extraversion increase with the number of words spoken by the partner (p-value < 0.01), consistent with *hypothesis 3* and the findings of [20] who also find talkative subjects are rated as more extraverted. The coefficient for number of words remains similar even after adding valence, arousal and dominance as explanatory variables in column 2. Column 3 shows that the result persists even after controlling for the player's IQ, eyes test score, age, gender, beliefs about partner's IQ, a dummy for non-native speaker (equals 1 if the player is a non-native English speaker and 0 otherwise) and a dummy for first speaker (equals 1 if the player started the conversation and 0 otherwise). Columns 4-6 show that beliefs about partner's neuroticism decrease with the number of words spoken by the partner, although the impact is insignificant. Valence, arousal and dominance ratings did not have a significant impact on beliefs about either of the two fundamental personality traits. We also consider whether the beliefs formed by examining the number of words used in communication provide an *accurate* picture of someone's true personality type. What we see from the results in Table A.14 is that extraverts

[2]We might also consider the choice of words used by participants. Figure A.4 shows a word cloud of the words spoken by the subjects during the pre-game small talk communication which depicts the very general and trivial nature of small talk. Figure A.5 attempts to distinguish between the most frequently used words by subjects believed to have different personalities. Through a simple examination of word usage, it's hard to distinguish between the nature of language used by subjects believed to have different personalities. Those who are believed to be highly extraverted (believed to have above median extraversion scores) have a similar set of most frequently used words when compared to those who are believed to be less extraverted (believed to have below median extraversion scores) which are likely to reflect the social norms of small talk (Figures A.5 (A) and (B)). Figures A.5 (C) and (D) show a similar story for neuroticism beliefs. This finding is not surprising given the unstructured nature of the small talk but we know from our results and experimental design that language is playing an important role, so we will focus on more quantitative measures here.

Table A.1. Impact of number of words and emotional content of the text spoken by the partner on beliefs about partner's personality

	Extraversion Belief			Neuroticism Belief		
	(1)	(2)	(3)	(4)	(5)	(6)
Number of Words	0.2744***	0.2604***	0.2355***	-0.0573	-0.0368	-0.0455
	(0.079)	(0.076)	(0.078)	(0.072)	(0.076)	(0.073)
Valence		-0.2723	-0.2545		-0.0578	0.0328
		(0.233)	(0.234)		(0.263)	(0.267)
Arousal		0.1718	0.2191		-0.0763	-0.1241
		(0.149)	(0.147)		(0.116)	(0.115)
Dominance		0.1918	0.1728		0.0361	-0.0407
		(0.256)	(0.253)		(0.256)	(0.255)
Own IQ			-0.1178			0.1278
			(0.086)			(0.079)
Eyes Test Score			0.0603			0.0276
			(0.058)			(0.097)
Age			0.0227			-0.0429**
			(0.022)			(0.020)
Female			-0.0659			-0.1659
			(0.157)			(0.157)
IQ Belief			0.1327			-0.0978
			(0.081)			(0.086)
Non-Native Speaker			0.3788**			-0.2491
			(0.151)			(0.158)
First Speaker			-0.0036			-0.3199**
			(0.140)			(0.152)
N	168	168	168	168	168	168

Standard errors in parentheses

* $p < 0.10$, ** $p < 0.05$, *** $p < 0.01$

The explanatory variables, namely number of words, and valence, arousal and dominance ratings are standardised for comparability across coefficients.

genuinely do seem to use more words, a result which is significant (p-value < 0.05), with and without the addition of valence, arousal and dominance as explanatory variables and a list of sensible control variables.

Overall, we find evidence suggesting that beliefs about a partner's fundamental personality traits, particularly extraversion, are a significant determinant of decisions made in any subsequent strategic interaction with them. This impact of beliefs on choices can either be through the absolute value of beliefs about partner's extraversion (as in the public goods game) or the perceived differences in the pair's extraversion (as in the 11-20 money request game). We hope that our study might open avenues for future research exploring how beliefs about other's personality traits affect choices made in various strategic interactions with them. Being the first study of its kind, our work is limited in scope due to the controlled laboratory setting and limited communication time, making it impossible to examine the role of the remaining personality traits, agreeableness, conscientiousness and openness, and even intelligence. In order to give these traits a more reasonable chance of playing a role, a longer, more sustained series of small talk conversations, something more akin to what occurs in the real-world seems sensible, which is only going to be feasible in the setting of a field experiment. Our hope

is that our results will give impetus to new research that looks at repeated interactions in a more realistic setting.

B Additional Tables and Figures

Here we present additional tables and figures referenced in the main text or discussion.

Table A.2. Summary Statistics

Variable	Mean	Std. Dev.	Min.	Max.	N
Own Extraversion	3.372	0.814	1.25	5	338
Own Neuroticism	2.935	0.811	1	5	338
Extraversion Belief	3.499	0.827	1	5	338
Neuroticism Belief	2.818	0.865	1	5	338
Perceived diff Extraversion	0.882	0.689	0	3.25	338
Perceived diff Neuroticism	0.899	0.714	0	3.125	338
Level Chosen in 11-20 game	2.891	2.522	0	9	338
Level Belief in 11-20 game	2.787	2.566	0	9	338
Own Contribution in PGG	11.163	7.363	0	20	338
Contribution Belief in PGG	11.633	6.956	0	20	338
Own IQ	18.604	4.464	4	28	338
IQ Belief	18.213	4.825	1	30	338
Eyes Test Score	27.817	3.759	11	35	338
Age	21.154	3.622	17	42	338
Risk Aversion	4.317	0.767	1.533	6	338
Female	0.615	0.487	0	1	338
Non-native English speaker	0.349	0.477	0	1	338

Table A.3. Balance Test for Treatment and Control groups

Variable	(1) Control Mean/SE	(2) Treatment Mean/SE	T-test P-value
Own Extraversion	3.4213	3.3222	0.2632
	(0.0639)	(0.0612)	
Own Neuroticism	2.9529	2.9174	0.6880
	(0.0611)	(0.0639)	
Eyes Test Score	27.4706	28.1667	0.0888*
	(0.2516)	(0.3217)	
Age	20.9353	21.3750	0.2650
	(0.2765)	(0.2805)	
Female	0.6529	0.5774	0.1543
	(0.0366)	(0.0382)	
Own IQ	18.2059	19.0060	0.0996*
	(0.3434)	(0.3417)	
Risk Aversion	4.2863	4.3474	0.4645
	(0.0600)	(0.0580)	
Non-native English speaker	0.3588	0.3393	0.7074
	(0.0369)	(0.0366)	
N	170	168	
F-test of joint significance (p-value)			0.2363
F-test, number of observations			338

Statistical significance indicated as follows:
* $p < 0.10$, ** $p < 0.05$, *** $p < 0.01$

Table A.4. Overestimation and inaccuracy of personality beliefs

	Overestimation of Extraversion Belief		Inaccuracy of Extraversion Belief	Overestimation of Neuroticism Belief		Inaccuracy of Neuroticism Belief
	(1)	(2)	(3)	(4)	(5)	(6)
OwnExtraversion × Treatment	0.1601*	0.2170**	-0.0132	-0.0760	-0.0954	0.0489
	(0.086)	(0.092)	(0.112)	(0.092)	(0.100)	(0.118)
OwnNeuroticism × Treatment	0.1040	0.1121	0.1135	-0.0404	-0.0321	-0.0213
	(0.093)	(0.096)	(0.119)	(0.085)	(0.083)	(0.117)
PartnerExtraversion × Treatment	0.3031***	0.3075***	-0.3722***			
	(0.079)	(0.081)	(0.124)			
PartnerNeuroticism × Treatment				0.0169	-0.0004	0.4717***
				(0.078)	(0.078)	(0.131)
Eyes Test Score × Treatment	0.0663	0.0773	-0.1817*	0.1146	0.1503	-0.0833
	(0.072)	(0.072)	(0.105)	(0.099)	(0.102)	(0.125)
Own Extraversion	0.0101	0.0181	0.1391*	-0.0740	-0.0552	-0.0261
	(0.052)	(0.059)	(0.082)	(0.058)	(0.058)	(0.073)
Own Neuroticism	-0.0061	0.0006	0.0144	0.0343	0.0461	-0.0194
	(0.062)	(0.064)	(0.094)	(0.062)	(0.061)	(0.091)
Partner's Extraversion	-0.8160***	-0.8189***	-0.0259			
	(0.052)	(0.055)	(0.091)			
Partner's Neuroticism				-0.6530***	-0.6395***	-0.1162
				(0.053)	(0.054)	(0.098)
Eyes Test Score	-0.0459	-0.0368	0.1731**	-0.0930	-0.1352*	-0.0816
	(0.054)	(0.057)	(0.080)	(0.070)	(0.074)	(0.090)
Treatment	0.2609***	-0.2290	1.0108	-0.3866***	-0.1525	-0.4028
	(0.071)	(0.463)	(0.613)	(0.080)	(0.423)	(0.576)
Controls	No	Yes	Yes	No	Yes	Yes
N	338	338	338	338	338	338

Standard errors in parentheses. Statistical significance indicated as follows:

* $p < 0.10$, ** $p < 0.05$, *** $p < 0.01$

The dependant variable, Overestimation of personality beliefs, is computed by taking the difference between the player's beliefs about their partner's personality and the partner's true personality scores. This difference is then standardised. The dependent variable is thus a measure of exaggeration or overestimation of the partner's personality by the player. The dependent variable, Inaccuracy of personality beliefs, is computed by taking the absolute difference between the player's beliefs about their partner's personality and the partner's true personality scores. This difference is then standardised. This dependent variable is thus a measure of the error or inaccuracy in the player's beliefs about their partner's personality. The independent variables are the player's own personality traits, the true personality trait score of the partner, the player's eyes test score and these variables interacted with the treatment dummy. The control variables are the player's IQ, gender, age and risk aversion and these variables interacted with the treatment dummy. Columns 1 and 2 show that overestimation of partner's extraversion increases with the player's own extraversion, an effect which is significantly stronger in the treatment group compared to the control group. In column 3, the negative significant (p-value < 0.10) interaction term between the player's eyes test score and the treatment dummy shows that with increasing eyes test score, the inaccuracy in the player's beliefs about partner's extraversion is significantly lower in the treatment group compared to the control. Columns 4 and 5 show no significant effect of own extraversion or neuroticism on overestimation of the partner's neuroticism in either of the two groups. Column 6 shows that the player's performance in the eyes test has no significant impact on the inaccuracy of their beliefs about partner's neuroticism.

Table A.5. Impact of beliefs about own cognitive ability on beliefs about partner's cognitive ability

	IQ Belief		Overestimation of IQ Belief		Inaccuracy of IQ Belief	
	(1)	(2)	(3)	(4)	(5)	(6)
Own IQ Belief × Treatment	-0.0588	-0.0626	-0.0445	-0.0474	-0.1807	-0.3183**
	(0.086)	(0.116)	(0.065)	(0.088)	(0.112)	(0.143)
Partner's IQ × Treatment	-0.0345	-0.0186	-0.0261	-0.0141	0.0912	0.0881
	(0.081)	(0.082)	(0.061)	(0.062)	(0.148)	(0.145)
Own IQ belief	0.6706***	0.7319***	0.5077***	0.5541***	-0.1120	0.0198
	(0.060)	(0.078)	(0.045)	(0.059)	(0.079)	(0.105)
Partner's IQ	0.0937*	0.0894*	-0.6296***	-0.6328***	-0.1668**	-0.1588**
	(0.050)	(0.050)	(0.038)	(0.038)	(0.082)	(0.077)
Treatment	-0.0833	0.4362	-0.0631	0.3303	0.0693	0.3750
	(0.082)	(0.506)	(0.062)	(0.383)	(0.108)	(0.625)
Own IQ × Treatment		-0.0172		-0.0130		0.1404
		(0.110)		(0.083)		(0.122)
Eyes Test Score × Treatment		0.0276		0.0209		0.1534
		(0.099)		(0.075)		(0.122)
Own IQ		-0.0714		-0.0541		-0.1146
		(0.069)		(0.053)		(0.087)
Eyes Test Score		0.0194		0.0147		-0.1784**
		(0.077)		(0.058)		(0.082)
Controls	No	Yes	No	Yes	No	Yes
N	338	338	338	338	338	338

Standard errors in parentheses. Statistical significance indicated as follows:
* $p < 0.10$, ** $p < 0.05$, *** $p < 0.01$

Column 1 examines the impact of the player's beliefs about own IQ, partner's true IQ and their interaction with the treatment dummy, on beliefs about the partner's IQ. While own IQ belief interacted with treatment dummy has no significant effect, own IQ belief positively impacts beliefs about partner's IQ. Column 2 includes the player's (i.e. the predictor's) true IQ as measured by the Raven's test, the player's eyes test score, along with their interactions with the treatment dummy. Columns 2 also includes the control variables - player's age, gender and risk aversion - and the 3 control variables interacted with the treatment dummy. For columns 3 and 4 the dependant variable is the standardised difference between the beliefs about partner's IQ and the partner's true IQ (as measured by the partner's performance in the Raven's test). Hence, for columns 3 and 4 the dependant variable is a measure of the degree by which the player overestimates their partner's IQ. Columns 3 and 4 indicate that an increase in player's own IQ belief leads to overestimation of the partner's IQ, irrespective of being in the treatment or control group i.e. players project beliefs about their own IQ onto their partner. For columns 5 and 6 the dependant variable is the standardised absolute difference between the beliefs about partner's IQ and the partner's true IQ. Hence, for columns 5 and 6 the dependant variable is a measure of the inaccuracy in the player's beliefs about their partner's IQ. In column 6, the significant (p-value < 0.05) negative interaction between own IQ belief and the treatment dummy, implies that as own IQ belief increases, the inaccuracy in beliefs about partner's IQ is significantly lower in the treatment group compared to the control.

Table A.6. Impact of (absolute) difference between own personality and beliefs about partner's personality on level-k strategy chosen

	Level Belief			Level Chosen		
	(1)	(2)	(3)	(4)	(5)	(6)
DiffExtraversion × Treatment	-0.5302*	-0.5600*	-0.5396*	-0.6597***	-0.7395***	-0.6505**
	(0.269)	(0.290)	(0.299)	(0.237)	(0.242)	(0.254)
DiffNeuroticism × Treatment	0.1879	0.2106	0.3353	-0.0415	-0.0060	0.1645
	(0.248)	(0.263)	(0.292)	(0.248)	(0.244)	(0.262)
DiffExtraversion	0.1470	0.1806	0.1448	0.2046	0.2089	0.1663
	(0.198)	(0.201)	(0.204)	(0.177)	(0.180)	(0.183)
DiffNeuroticism	-0.1579	-0.1491	-0.2589	-0.1604	-0.1499	-0.2929
	(0.183)	(0.190)	(0.211)	(0.174)	(0.182)	(0.189)
Treatment	0.1668	0.0079	-2.9732	0.0677	-0.0799	-2.3533
	(0.267)	(0.285)	(2.048)	(0.279)	(0.286)	(1.854)
Own Extraversion × Treatment		-0.0578	0.0509		-0.1528	0.0320
		(0.306)	(0.364)		(0.289)	(0.317)
Own Neuroticism × Treatment		-0.1846	-0.1402		-0.4226	-0.4142
		(0.277)	(0.303)		(0.280)	(0.280)
Own Extraversion		-0.0612	-0.1633		-0.1773	-0.2771
		(0.196)	(0.203)		(0.214)	(0.216)
Own Neuroticism		0.0227	-0.0960		0.2069	0.0490
		(0.197)	(0.215)		(0.201)	(0.200)
Extraversion Belief × Treatment		-0.2863	-0.2942		-0.2422	-0.2628
		(0.272)	(0.284)		(0.255)	(0.263)
Neuroticism Belief × Treatment		-0.2533	-0.1483		-0.2287	-0.1007
		(0.292)	(0.303)		(0.282)	(0.297)
Extraversion Belief		0.1724	0.1403		0.1498	0.1261
		(0.194)	(0.197)		(0.183)	(0.179)
Neuroticism Belief		-0.1412	-0.1978		-0.0924	-0.1278
		(0.195)	(0.196)		(0.203)	(0.209)
Eyes Test Score × Treatment			0.5905*			0.6297**
			(0.301)			(0.311)
Own IQ × Treatment			-0.2809			-0.3072
			(0.296)			(0.307)
IQ Belief × Treatment			0.3462			0.2073
			(0.324)			(0.270)
Female × Treatment			-0.8057			-0.8928
			(0.612)			(0.564)
Order × Treatment			1.1902**			1.1083*
			(0.594)			(0.605)
Eyes Test Score			-0.4462*			-0.4522*
			(0.249)			(0.254)
Own IQ			0.2175			0.2606
			(0.203)			(0.220)
IQ Belief			-0.3538*			-0.3321*
			(0.210)			(0.200)
Female			1.1196**			1.4361***
			(0.435)			(0.388)
Order			-0.7462*			-0.9827**
			(0.401)			(0.420)
Controls	No	No	Yes	No	No	Yes
N	338	338	338	338	338	338

Standard errors in parentheses. Statistical significance indicated as follows:
* $p < 0.10$, ** $p < 0.05$, *** $p < 0.01$

Here, 'Controls' imply the player's risk aversion, age and their interactions with the treatment dummy.

Table A.7. Impact of (absolute) difference between own personality and beliefs about partner's personality on the probability of choosing the best response - Probit Model

	Control		Treatment	
	(1) Pr(Level=2)	(2) Pr(Level=2)	(3) Pr(Level=2)	(4) Pr(Level=2)
DiffExtraversion	-0.0453	-0.0550	0.0846***	0.0992***
	(0.038)	(0.035)	(0.030)	(0.032)
DiffNeuroticism	-0.0008	-0.0077	-0.0459	-0.0358
	(0.031)	(0.031)	(0.032)	(0.033)
Own Extraversion		0.0123		0.0168
		(0.030)		(0.046)
Own Neuroticism		0.0543*		-0.0438
		(0.032)		(0.036)
Extraversion Belief		-0.0165		-0.0109
		(0.035)		(0.031)
Neuroticism Belief		0.0296		0.0656*
		(0.033)		(0.035)
Own IQ		0.0587*		0.0558
		(0.036)		(0.038)
IQ Belief		-0.0441		-0.0028
		(0.029)		(0.036)
Eyes Test Score		0.0549		0.0497
		(0.037)		(0.031)
Controls	No	Yes	No	Yes
N	170	170	168	168

Standard errors in parentheses. Statistical significance indicated as follows:
* $p < 0.10$, ** $p < 0.05$, *** $p < 0.01$

The table reports the average marginal effects from Probit regressions. 'Controls' imply the player's age, gender, risk aversion and the order of play of the two games.

Table A.8. Impact of (absolute) difference between own personality and beliefs about partner's personality on the probability of choosing the best response - Logit Model

	Control		Treatment	
	(1) Pr(Level=2)	(2) Pr(Level=2)	(3) Pr(Level=2)	(4) Pr(Level=2)
DiffExtraversion	-0.0486	-0.0547	0.0843***	0.1016***
	(0.041)	(0.040)	(0.029)	(0.030)
DiffNeuroticism	-0.0019	-0.0074	-0.0459	-0.0370
	(0.030)	(0.031)	(0.032)	(0.032)
Own Extraversion		0.0093		0.0185
		(0.030)		(0.047)
Own Neuroticism		0.0548		-0.0422
		(0.034)		(0.037)
Extraversion Belief		-0.0154		-0.0102
		(0.039)		(0.032)
Neuroticism Belief		0.0321		0.0665*
		(0.035)		(0.035)
Own IQ		0.0618		0.0583
		(0.038)		(0.040)
IQ Belief		-0.0428		-0.0042
		(0.027)		(0.037)
Eyes Test Score		0.0531		0.0454
		(0.038)		(0.033)
Controls	No	Yes	No	Yes
N	170	170	168	168

Standard errors in parentheses. Statistical significance indicated as follows:
* $p < 0.10$, ** $p < 0.05$, *** $p < 0.01$

The table reports the average marginal effects from Logit regressions. 'Controls' imply the player's age, gender, risk aversion and the order of play of the two games.

Table A.9. Impact of beliefs about partner's personality on beliefs about partner's contribution and own contribution in the public goods game

	Control Order 1				Treatment Order 1			
	(1) Contribution Belief	(2) Contribution Belief	(3) Own Contribution	(4) Own Contribution	(5) Contribution Belief	(6) Contribution Belief	(7) Own Contribution	(8) Own Contribution
Extraversion Belief	0.0430	0.0575	0.0951	0.1042	0.1964*	0.1879*	0.1882**	0.1667*
	(0.083)	(0.082)	(0.087)	(0.101)	(0.101)	(0.100)	(0.087)	(0.083)
Neurotcisim Belief	0.0440	0.0456	-0.0207	-0.0275	0.1771	0.1627	0.1591	0.1697
	(0.090)	(0.109)	(0.087)	(0.101)	(0.111)	(0.109)	(0.117)	(0.112)
Own IQ		-0.0664		-0.0114		0.1265		0.1782*
		(0.106)		(0.087)		(0.088)		(0.101)
IQ Belief		0.1329		0.1016		0.0964		0.2512**
		(0.097)		(0.107)		(0.096)		(0.097)
Eyes Test Score		-0.0256		0.0221		0.1197		0.1694
		(0.096)		(0.130)		(0.090)		(0.117)
Controls	No	Yes	No	Yes	No	Yes	No	Yes
N	110	110	110	110	106	106	106	106

Standard errors in parentheses. Statistical significance indicated as follows:
* $p < 0.10$, ** $p < 0.05$, *** $p < 0.01$

This table shows that of the two fundamental personality traits - extraversion and neuroticism - only beliefs about partner's extraversion affect decision making in the public goods game, for treatment group subjects. 'Controls' refers to the player's age, gender and risk aversion.

Table A.10. Impact of beliefs about partner's personality and own personality on beliefs about partner's contribution and own contribution in Public Goods Game - OLS approach

	Control OLS		Treatment OLS	
	(1) Contribution Belief	(2) Own Contribution	(3) Contribution Belief	(4) Own Contribution
ExtraversionBelief	0.0601	0.1110	0.2036**	0.1599*
	(0.082)	(0.092)	(0.099)	(0.085)
OwnExtraversion	-0.0733	-0.2041**	-0.1831	-0.0919
	(0.095)	(0.088)	(0.118)	(0.117)
Own IQ	-0.0583	-0.0417	0.0783	0.1484
	(0.096)	(0.084)	(0.086)	(0.099)
IQ Belief	0.1250	0.1140	0.0953	0.2474**
	(0.091)	(0.100)	(0.096)	(0.099)
Eyes Test Score	-0.0431	-0.0015	0.1328	0.1754
	(0.096)	(0.118)	(0.099)	(0.127)
Controls	Yes	Yes	Yes	Yes
N	110	110	106	106

Standard errors in parentheses. Statistical significance indicated as follows:
* $p < 0.10$, ** $p < 0.05$, *** $p < 0.01$

'Controls' refers to the player's age, gender and risk aversion.

Table A.11. Impact of beliefs about partner's personality and own personality facets on beliefs about partner's contribution and own contribution in Public Goods Game

	Control OLS		Treatment IV	
	(1) Contribution Belief	(2) Own Contribution	(3) Contribution Belief	(4) Own Contribution
ExtraversionBelief	0.0542	0.1036	0.6169**	0.5262**
	(0.084)	(0.093)	(0.265)	(0.251)
OwnAssertiveness	-0.1258	-0.2271*	-0.3287**	-0.3095**
	(0.113)	(0.114)	(0.128)	(0.124)
OwnActivity	0.0593	0.0333	0.0255	0.1562
	(0.122)	(0.123)	(0.125)	(0.106)
Own IQ	-0.0497	-0.0323	0.0781	0.1396
	(0.099)	(0.088)	(0.098)	(0.105)
IQ Belief	0.1391	0.1301	0.1041	0.2708***
	(0.089)	(0.102)	(0.091)	(0.092)
Eyes Test Score	-0.0342	0.0114	0.1193	0.1751
	(0.102)	(0.122)	(0.118)	(0.139)
Controls	Yes	Yes	Yes	Yes
N	110	110	106	106

Standard errors in parentheses. Statistical significance indicated as follows:
* $p < 0.10$, ** $p < 0.05$, *** $p < 0.01$

'Controls' refers to the player's age, gender and risk aversion. Columns 1 and 2 report the OLS regression results for the control group. Column 2 shows that the players own assertiveness has a negative significant effect (p-value < 0.05) on contribution levels whereas facet activity has an insignificant positive effect. None of the facets significantly impact beliefs about partner's contribution. Columns 3 and 4 present the results from 2SLS IV regression for the treatment group. For the treated subjects, beliefs about partner's extraversion positively and significantly (p-value < 0.05) affects beliefs about partner's contribution as well as own-contribution. With regards to the player's own personality, facet assertiveness has a significant negative effect (p-value < 0.05) on both contribution belief and own-contribution, whereas facet activity has an insignificant positive effect.

Table A.12. Impact of beliefs about partner's personality and own personality on beliefs about partner's contribution and own contribution in Public Goods Game - Order 2

	Control OLS		Treatment IV	
	(1) Contribution Belief	(2) Own Contribution	(3) Contribution Belief	(4) Own Contribution
Extraversion Belief	-0.0357	-0.2345*	0.1273	1.2682
	(0.147)	(0.121)	(1.065)	(1.986)
Own Extraversion	0.1603	0.0317	0.1219	-0.1167
	(0.158)	(0.158)	(0.189)	(0.321)
Own IQ	0.1372	0.0435	-0.0345	-0.0495
	(0.203)	(0.162)	(0.120)	(0.223)
IQ Belief	0.1792	0.0170	-0.0657	-0.1679
	(0.159)	(0.133)	(0.142)	(0.209)
Eyes Test Score	-0.2673	0.2327	0.2574	0.0801
	(0.174)	(0.164)	(0.157)	(0.330)
Controls	Yes	Yes	Yes	Yes
N	60	60	62	62

Standard errors in parentheses. Statistical significance indicated as follows:
* $p < 0.10$, ** $p < 0.05$, *** $p < 0.01$

This table replicates the IV regression results from the main paper but only for those subjects which played the 11-20 game first. 'Controls' refers to the player's age, gender and risk aversion.

Table A.13. Impact of beliefs about partner's personality and own personality on beliefs about partner's contribution and own contribution in Public Goods Game - for both orders of play

	Control OLS		Treatment IV	
	(1) Contribution Belief	(2) Own Contribution	(3) Contribution Belief	(4) Own Contribution
ExtraversionBelief	0.0229	-0.0031	0.4261	0.5759*
	(0.071)	(0.075)	(0.275)	(0.301)
OwnExtraversion	-0.0007	-0.1202	-0.1269	-0.1544
	(0.083)	(0.080)	(0.107)	(0.115)
Own IQ	-0.0135	-0.0213	0.0437	0.0390
	(0.087)	(0.074)	(0.076)	(0.093)
IQ Belief	0.1426*	0.0827	0.0050	0.0824
	(0.081)	(0.082)	(0.072)	(0.087)
Eyes Test Score	-0.1205	0.0825	0.1323	0.1295
	(0.082)	(0.092)	(0.085)	(0.113)
Order	-0.0876	0.0501	-0.0520	-0.1242
	(0.181)	(0.162)	(0.190)	(0.208)
Controls	Yes	Yes	Yes	Yes
N	170	170	168	168

Standard errors in parentheses

* $p < 0.10$, ** $p < 0.05$, *** $p < 0.01$

This table replicates the IV regression results from the main paper for both orders of play. Order is a dummy variable that equals 1 when the 11-20 game is played first and 0 when the public goods game is played first. 'Controls' refers to the player's age, gender and risk aversion. The table shows that playing the 11-20 game first has a negative impact on both beliefs about partner's contribution as well as own contribution in the public goods game for the Treatment group, although the effect is statistically insignificant.

Table A.14. Relationship between number of words and emotional content of text spoken by the subject and the subject's own personality

	Own Extraversion			Own Neuroticism		
	(1)	(2)	(3)	(4)	(5)	(6)
Own Number of Words	0.1439**	0.1733**	0.1781**	0.1289*	0.1282*	0.0814
	(0.071)	(0.073)	(0.075)	(0.071)	(0.071)	(0.073)
Own Valence		-0.5325*	-0.5533**		0.2299	0.3302
		(0.284)	(0.276)		(0.269)	(0.255)
Own Arousal		0.0296	0.0358		-0.2117	-0.2409*
		(0.150)	(0.152)		(0.135)	(0.132)
Own Dominance		0.4011	0.4033		-0.0124	-0.0905
		(0.279)	(0.272)		(0.258)	(0.252)
Own IQ			-0.1804**			-0.0031
			(0.085)			(0.076)
Eyes Test Score			-0.0069			0.1478*
			(0.090)			(0.084)
Age			0.0210			0.0021
			(0.027)			(0.023)
Female			0.0201			0.3858**
			(0.169)			(0.162)
Non-Native Speaker			0.0798			-0.1319
			(0.156)			(0.174)
First Speaker			0.1562			0.1071
			(0.156)			(0.162)
N	168	168	168	168	168	168

Standard errors in parentheses

* $p < 0.10$, ** $p < 0.05$, *** $p < 0.01$

The explanatory variables, namely the number of words spoken by the subject, and valence, arousal and dominance ratings of language used, are standardised for comparability across coefficients. Number of words used by the subject is a positive indicator of the subject's extraversion, even after adding valence, arousal and dominance ratings of the text used as explanatory variables. The result persists after controlling for the subject's IQ, eyes test score, age, gender, a dummy for non-native speaker (equals 1 if the subject is a non-native English speaker and 0 otherwise) and a dummy for first speaker (equals 1 if the subject started the conversation and 0 otherwise). Trait neuroticism also appears to be positively associated with number of words used, although the coefficient becomes insignificant after adding sensible control variables.

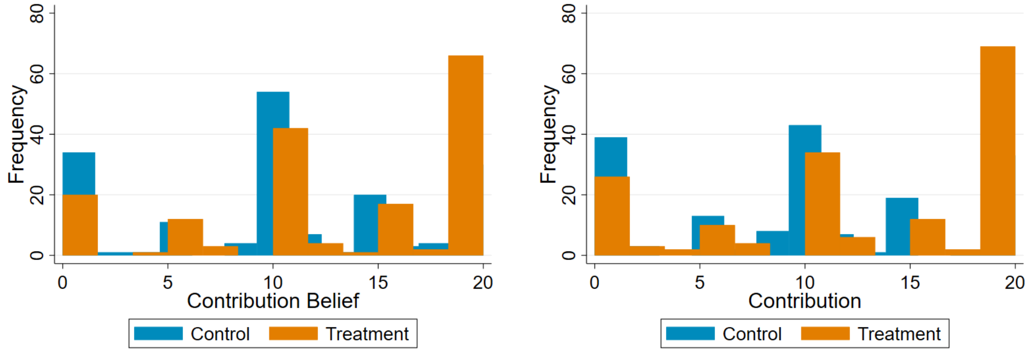

(A) Contribution Belief **(B)** Own Contribution

Fig A.1. Distribution of (A) Beliefs about Partner's Contribution and (B) Own Contribution in the Public Goods Game

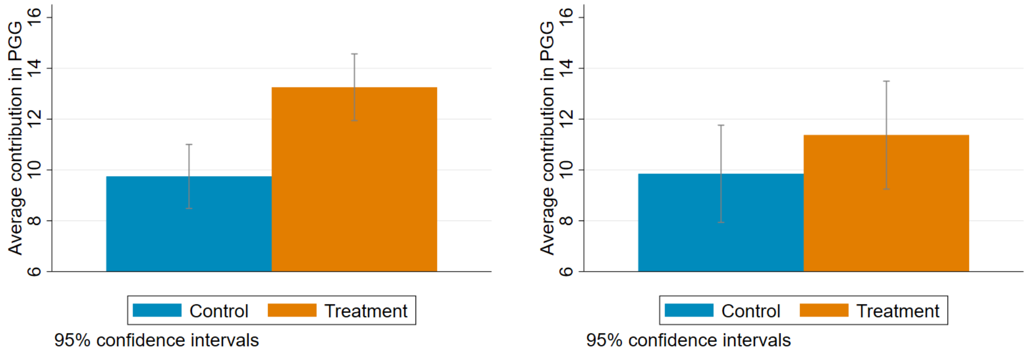

(A) Order 1 **(B)** Order 2

Fig A.2. Average contribution in Public Goods Game (PGG) (A) when PGG is played first (order 1) and (B) when the 11-20 game is played first (order 2). Treated subjects contribute more than control group subjects in order 1. The average contribution of treated subjects is 13.2 EP where as that of control group subjects is 9.7 EP in order 1. The difference is statistically significant with t-statistic of -3.8060 and p-value < 0.01. There is no significant difference in contribution levels between the treatment and control groups in order 2.

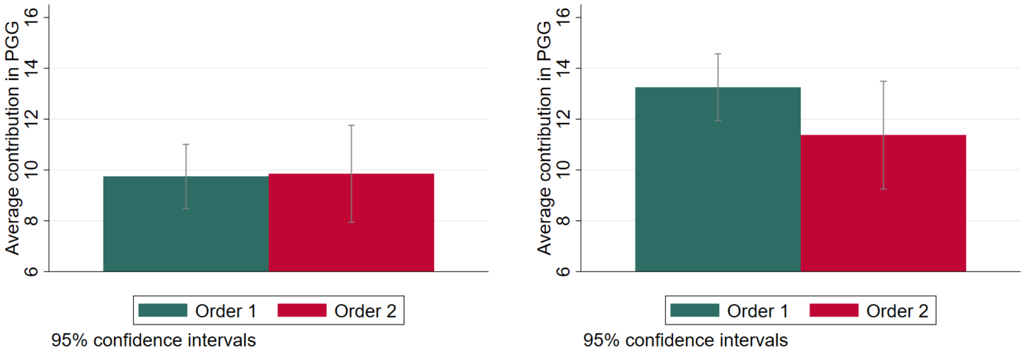

(A) Control **(B)** Treatment

Fig A.3. Average contribution in Public Goods Game (PGG) for different orders of play of the two games for (A) Control and (B) Treatment groups. Order 1 is when PGG is played first and order 2 is when the 11-20 game is played first. On average players contribute more in the treatment group (figure (b)) when PGG is played first (order 1) compared to when 11-20 is played first (order 2). In a one-tailed t-test, we reject the null of no significant difference in contribution between treated players in order 1 and treated players in order 2 in favour of the alternative that treated players in order 1 contribute more at the 10% significance level (t-statistic = 1.5752, p-value =0.0586). There is no significant difference for control group subjects (figure (A)).

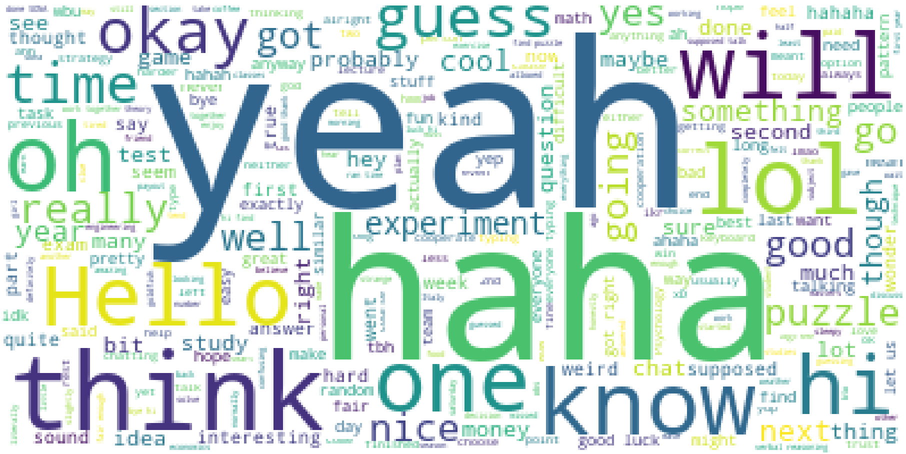

Fig A.4. Most frequently used words by subjects during small talk communication

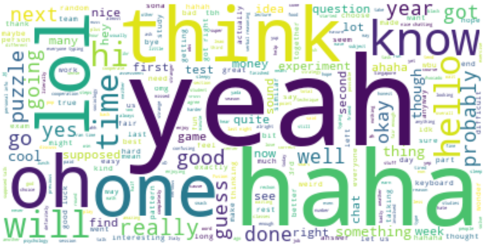

(A) Highly Extraverted

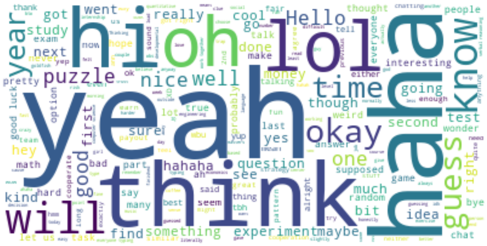

(B) Less Extraverted

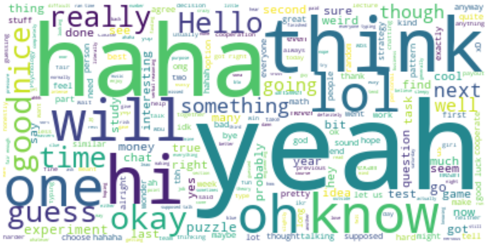

(C) Highly Neurotic

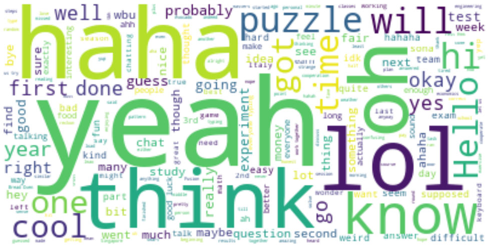

(D) Less Neurotic

Fig A.5. Most frequently used words during small talk communication by subjects who are believed to be (A) highly extraverted (B) less extraverted (C) highly neurotic and (D) less neurotic.

C Examples of Small Talk Communication

Example 1

Player 1: *hey*

Player 2: *Hey how are you doing :)*

Player 1: *lol alright*

Player 1: *you*

Player 2: *yeah fine haha*

Player 1: *tbh this is strange*

Player 2: *this is strange*

Player 2: *exactly haha*

Player 1: *omg*

Player 1: *so...*

Player 1: *do you have any pets?*

Player 2: *probably they want to see if we will cooperate depending on our chat or something haha*

Player 2: *nope and you?*

Player 1: *trying to make conversation :D*

Player 1: *yep, two cats*

Player 2: *I had fish when I was little haha*

Player 2: *What are their names?*

Player 1: *aww like goldfish?*

Player 1: *Cosmos and Titan*

Player 2: *Yes a goldfish and one more but I forgot the type lol*

Player 2: *That is great!*

Player 1: *i used to have goldfish*

Player 1: *but we could not keep them cause of the cats*

Player 2: *Goldfish live a long I think generally haha*

Player 2: *Oh no!*

Player 1: *we had 4 goldfish*

Player 2: *Cats is more interesting haha*

Player 2: *are**

Player 1: *yeah i know*

Player 1: *only problem is they scratch you*

Player 1: *a lot*

Player 2: *Ahaha yes*

Player 2: *scars all the time*

Player 1: *so now i have lots of marks on me*

Player 2: *This keyboard is so bad*

Player 2: *Oh no*

Player 2: *The pain of being a cat owner haha*

Player 1: *the keyboard never crossed my mind lol*

Player 2: *I barely can type on it haha*

Player 2: *It was nice chatting to you haha*

Player 1: *aww goodbye*

Example 2

Player 1: *hi*

Player 2: *hey*

Player 1: *what is up?*

Player 2: *not much, you?*

Player 1: *same, just waiting haha*

Player 2: *same, it is a bit dead is not it*

Player 1: *it really is...*

Player 2: *think I mucked up most of those puzzles tbh*
Player 1: *although everyone is now typing fervently*
Player 1: *you think you did that bad?*
Player 2: *not that bad, but some of them I just did not get*
Player 2: *or I almost got them and then the time ran out*
Player 1: *there were some really weird ones though*
Player 2: *yeah igy*
Player 1: *yeah same, 30 seconds is a bit too quick for some of those*
Player 2: *some just made no sense to me*
Player 1: *true that*
Player 1: *but they take 2/30 anyway,*
Player 2: *seems like a bit of a waste of time*
Player 2: *to do 30 and then only 2 count*
Player 1: *and for some reason \ q random \ q selection always ends up in me being paid nothing xD*
Player 2: *same haha*
Player 1: *Ikr*
Player 2: *or i am in a team and the team does really badly and i get almost no money*
Player 1: *but yeah, pretty much a waste*
Player 2: *really**
Player 1: *omg yes....*
Player 2: *its a bit annoying*
Player 1: *These dictator games where in the end one person decides whether I can keep my money or get nothing*
Player 2: *yes! so irritating*
Player 1: *Being paid £3 after 1,5 hours....*
Player 2: *what a drag*
Example 3
Player 1: *Hi*
Player 2: *Hello*
Player 1: *how are you?*
Player 2: *How are you?*
Player 2: *haha*
Player 1: *haha i'm good you?*
Player 2: *great*
Player 2: *How are exams going?*
Player 1: *yeah not too bad, some have gone worse that i had wanted, you?*
Player 2: *Most of them were alright, three more to go*
Player 2: *How about you?*
Player 2: *Any more left?*
Player 1: *i've got 1 more to go, thank god, i have 7 overall*
Player 1: *how many do you have overall?*
Player 2: *That's a lot. When is your last one?*
Player 2: *I have 6 in total*
Player 1: *next wednesday*
Player 1: *so i can go to circle and pop and celebrate by getting black out drunk haha*
Player 2: *Still some time to prepare. I have one this Saturday*
Player 2: *Yeah, pop is back on again next week*
Player 1: *that's grim, my boyfriend does to, i don't get why exams on saturday is a thing*
Player 1: **too*
Player 2: *None of your 7 exams were on Saturday?*

Player 1: *nope, i had 1 in week 3, 1 week 4, 3 last week, 1 this week and one next week*

Player 2: T*ime is running out heh*

D Experiment Script

This following part is read out by the experimenter (Note that the script presented includes certain comments in italics which were not shown to subjects during the experiment).

Thank you everyone for coming to our experiment today. Before we begin, please check that the number on the card handed to you matches with the number on the cubicle that you are seated in.

During the whole experiment, please do not speak with each other. If you do not understand something, please ask the experimenter by raising your hand. We will come to you and answer your question individually. Please also refrain from using your mobile phones during the experiment.

Also bear in mind that you may have to wait a few moments during the experiment, as we want everyone to finish at the same time. You will see the message 'Please wait until the experiment continues' on your screen when this is applicable.

Before we begin, I would just like to say, that your participation is very crucial for our research and we truly appreciate all of you being here. Thank you.

We will now begin the experiment.

General Instructions

In the laboratory experiment you are taking part in, you can - depending on your decisions and the decisions of your fellow players - earn money in addition to the show-up fee of £4. It is, therefore, of importance that you read these instructions carefully.

Today's experiment consists of the following: In the first section, you will be asked to answer a few questions and solve some puzzles. In the second section, you will be asked to make decisions in a few tasks. Lastly, there will be some questions for you to answer.

Please note that the experiment will not involve any deception and your answers today will remain strictly anonymous. The generated anonymous data will only be used for the purpose of our study. Therefore, we request you to answer to the best of your ability as it is integral to our research.

The outcomes from each task will be disclosed at the end of the experiment.

Detailed instructions for each part will follow.

We will now begin the experiment.

(a) Questionnaire: Personality (44 questions)

You will be asked to answer some questions about yourself. Your payment will not be affected by this. Just to remind you, your answers will remain anonymous so please answer as truthfully as possible as this is critically important for our research.

You will see a number of characteristics that may or may not apply to you. For example, do you agree that you are someone who likes to spend time with others? Please pick an option next to each statement to indicate the extent to which you agree or disagree with that statement.

I see myself as someone who...

START BFI QUESTIONNAIRE

(b) PUZZLES: Raven's Test (30 items)

You will be asked to solve some puzzles, a pattern game.

On the screen, you will see a set of abstract pictures with one of the pictures missing. You need to choose a picture from the choices given below to complete the pattern. You will have 30 seconds to complete each set of pictures. The first picture you will see will be an example, no input is required. You will then be asked to solve a total of 30 such puzzles. 2 of these 30 puzzles will randomly be selected. For each correct answer, from the random 2, you will receive £1. Please make sure to click 'submit answer', as otherwise your answer will not be recorded, and you might lose money.

START RAVEN TEST

Out of the 30 puzzles you just saw, how many puzzles do you think you correctly solved?

If your answer to this question is correct, then you will win an additional £1.

Now subjects will be allocated to one of 2 groups - control or treatment.

Control Group

Placebo Task[3]

Can you please indicate the title and summarize the story of the last movie you have seen? Please be as specific as possible and include as many details as possible. Please use a minimum of 250 characters. You will have 4 minutes to write the summary.

Please write the summary in the box provided on the next screen.

(next screen) Please make sure to click 'Submit' after you are done, as otherwise your answer will not be recorded.

Beliefs

You have been randomly and anonymously matched with another person in this room who is participating in the experiment. Please answer a few questions about the other player to the best of your ability, before you proceed with the tasks.

1. You will see a number of characteristics that may or may not apply to the other player. For example, do you agree that the other player is someone who likes to spend time with others? Please pick an option next to each statement to indicate the extent to which you agree or disagree with the statement regarding the other player.

   You will see 11 statements about the other player.

   1 out of these 11 statements will be randomly chosen and if your answer matches that of the other player, then you will win an additional £1.

   START PERSONALITY PREDICTION QUESTIONNAIRE

2. Recall the visual puzzle task from earlier in the experiment. On the screen, you saw a set of abstract pictures with one of the pictures missing. You had to choose a picture from the choices given below to complete the pattern. You had 30 seconds to complete each set of pictures. You were asked to solve a total of 30 such puzzles. How many puzzles do you think the other player, with whom you have been matched, correctly solved? Please indicate a (whole) number between 0 and 30.

   If your answer to this question is correct, then you will win an additional of £1.

Tasks (note that the order of the two tasks below were randomised)

You will now take part in a few decision-making tasks with the player with whom you have already been matched. Note that you will be participating in all tasks with the same player. Your payoff from these tasks will be calculated in Experimental Pounds (EP). The exchange rate between £ and EP is 1:5, i.e. 5 EP = £1.

The outcomes from each task will be disclosed at the end of the experiment. You will receive payment based on your results from one of the tasks randomly selected from the tasks in this part of the experiment. Please note that each task is equally likely to be chosen for payment.

Task 1: PGG

You will now participate in a task with the player with whom you have been matched. You have 20 EP and the other player has 20 EP as well. Your task in the game, and also the other player's task, is to decide how much to contribute to a joint project. You can choose to contribute any amount between 0 and 20 EP (only integer numbers). Your earnings from the project is the total contribution to the project, made by you and the other player, multiplied by a factor of 3/4. Your payoff from this task will be your earnings from the project, plus the amount you did not contribute. Thus, your final payoffs (in EP) will be given by:

[3] This task has been adapted from the Placebo Task used in [21].

Your payoff = (20 − your contribution)+3/4(your contribution + the other player's contribution)

Other player's payoff = (20 − the other player's contribution)+3/4(your contribution + the other player's contribution)

If for example, you contribute 20 EP to the project and the other player contributes 20 EP then,

Your payoff will be: $20–20 + 3/4(20 + 20) = 30$

The other player's payoff will be: $20–20 + 3/4(20 + 20) = 30$

If for example, you contribute 0 EP to the project and the other player contributes 20 EP then,

Your payoff will be: $20–0 + 3/4(0 + 20) = 35$

The other player's payoff will be: $20–20 + 3/4(0 + 20) = 15$

If you have a question, please raise your hand.

If you have read the instructions and do not have any questions, please click 'OK' to proceed to a practice quiz. The quiz is to make sure that you understand the task and your answers will not affect your payoffs from the experiment.

Suppose you choose to contribute 20 EP and the other player chooses to contribute 0 EP.

Your payoff will be:

The other player's payoff will be:

Suppose you choose to contribute 10 EP and the other player chooses to contribute 14 EP.

Your payoff will be:

The other player's payoff will be:

You have correctly answered the practice quiz. Click 'Continue' to proceed with the task.

How much money do you think the other player will contribute? Please indicate a number (an integer) between 0 and 20.

If your answer to this question matches that of the other player, then you will win an additional £1.

How much would you like to contribute? Please choose a number (an integer) between 0 and 20.

Task 2: 11-20 money request game

You will now participate in a different task with the same player.

You and the other player are playing a game in which each player requests an amount of money. The amount must be (an integer) between 11 and 20 Experimental Pounds. Each player will receive the amount he or she requests. A player will receive an additional amount of 20 Experimental Pounds if he or she asks for exactly one Experimental Pound less than the other player.

If for example, you request 19 EP and the other player requests 20 EP then,

Your payoff will be: $19 + 20 = 39$

The other player's payoff will be: 20

If for example, you request 17 EP and the other player requests 16 EP then,

Your payoff will be: 17

The other player's payoff will be: $16 + 20 = 36$

If you have a question, please raise your hand.

If you have read the instructions and do not have any questions, please click 'OK' to proceed to a practice quiz. The quiz is to make sure that you understand the task and your answers will not affect your payoffs from the experiment.

Suppose you choose to request 13 EP and the other player chooses to request 14 EP.

Your payoff will be:

The other player's payoff will be:

Suppose you choose to request 15 EP and the other player chooses to request 18 EP.

Your payoff will be:

The other player's payoff will be:

You have correctly answered the practice quiz. Click 'Continue' to proceed with the task.

How much money do you think the other player will request? Please indicate a number (an integer) between 11 and 20.

If your answer to this question matches that of the other player, then you will win an additional £1.

What amount of money would you request? Please choose a number (an integer) between 11 and 20.

Treatment Group

Chat Instructions

You have been randomly and anonymously matched with another person in this room who is participating in the experiment.

Before you proceed with the tasks, you are allowed to chat with the other player for 4 minutes. You can type in the box provided at the bottom of the screen and press Enter on your keyboard to send your messages.

Your message should not contain any personal information such as your name or your computer ID. The purpose is to preserve anonymity throughout the experiment. You are allowed to chat freely in English and in a non-abusive manner.

Beliefs

Now that you have chatted with the other player please answer a few questions about the other player, before you proceed with the tasks.

1. You will see a number of characteristics that may or may not apply to the other player. For example, do you agree that the other player is someone who likes to spend time with others? Please pick an option next to each statement to indicate the extent to which you agree or disagree with the statement regarding the other player.

   You will see 11 statements about the other player.

   1 out of these 11 statements will be randomly chosen and if your answer matches that of the other player, then you will win an additional £1.

   START PERSONALITY PREDICTION QUESTIONNAIRE

2. Recall the visual puzzle task from earlier in the experiment. On the screen, you saw a set of abstract pictures with one of the pictures missing. You had to choose a picture from the choices given below to complete the pattern. You had 30 seconds to complete each set of pictures. You were asked to solve a total of 30 such puzzles. How many puzzles do you think the other player, with whom you chatted, correctly solved? Please indicate a (whole) number between 0 and 30.

   If your answer to this question is correct, then you will win an additional £1.

Tasks (note that the order of the two tasks below were randomised)

You will now take part in a few decision-making tasks with the player you chatted with. Note that you will be participating in all tasks with the same player. Your payoff from these tasks will be calculated in Experimental Pounds (EP). The exchange rate between £ and EP is 1:5, i.e. 5 EP = £1.

The outcomes from each task will be disclosed at the end of the experiment. You will receive payment based on your results from one of the tasks randomly selected from the tasks in this part of the experiment. Please note that each task is equally likely to be chosen for payment.

Task 1: PGG

You will now participate in a task with the player you chatted with. You have 20 EP and the other player has 20 EP as well. Your task in the game, and also the other player's task, is to decide how much to contribute to a joint project. You can choose to contribute any amount between 0 and 20 EP (only integer numbers). Your earnings from the project is the total contribution to the project, made by you and the other player, multiplied by a factor of ¾. Your payoff from this task will be your earnings from the project, plus the amount you did not contribute. Thus, your final payoffs (in EP) will be given by:

Your payoff = (20– your contribution)+$^3/_4$(your contribution + the other player's contribution)

Other player's payoff = (20– the other player's contribution)+$^3/_4$(your contribution + the other player's contribution)

Examples and quiz related to the game, then partner's strategy belief and own choice

Task 2: 11-20 money request game

You will now participate in a different task with the same player.

You and the other player are playing a game in which each player requests an amount of money. The amount must be (an integer) between 11 and 20 Experimental Pounds. Each player will receive the amount he or she requests. A player will receive an additional amount of 20 Experimental Pounds if he or she asks for exactly one Experimental Pound less than the other player.

Examples and quiz related to the game, then partner's strategy belief and own choice

FOR BOTH CONTROL AND TREATMENT:

Eyes Test (36 questions)

In this section, you will be asked to look at 36 pictures of different pairs of eyes.

For each set of eyes, choose the word which best describes what the person in the picture is thinking or feeling. You may feel that more than one word is applicable but please choose just one word, the word which you consider to be most suitable. Before making your choice, make sure that you have read all 4 words. You should try to do the task as quickly as possible, but you will not be timed. If you do not know what a word means you can read the meaning of the word provided at the bottom of the screen.

2 of these 36 questions you answer will randomly be selected. For each correct answer, from the random 2, you will receive £1.

You will first see a practice question with four options. The correct option will be highlighted. After that you may proceed to the questions.

Which word best describes what the person in the picture is thinking or feeling?

START EYES TEST

Questionnaire

Thank you. Now, in the final section, you will be asked to answer some questions about yourself.

(a) Risk

Please indicate the likelihood that you would engage in the described activity or behaviour if you were to find yourself in that situation.

START DOSPERT

(b) Personal information

1. How old are you? (in years)
2. What is your year of study? (1, 2, 3, Post-graduate Other)
3. What is your gender? (M, F, Other, Prefer not to say)
4. What is your nationality?
5. Is English your Native language? (Yes, No)

6. What is your current degree course?
7. Would you consider your degree course mostly: (quantitative, qualitative)
8. Have you ever taken any game theory modules/courses? (Yes, No)
9. How dissatisfied or satisfied are you with your life in general? (1-7 scale from completely dissatisfied to completely satisfied)

Profit display screen

1. Number of correct answers from the visual puzzles task (out of 30):
2. Your payoff (in EP) from the first decision-making task:
3. Your payoff (in EP) from the second decision-making task:
4. Number of correct answers from the eyes task (out of 36):
5. Additional amount earned (in £):
6. Total earnings (in £):

Thank you for completing the experiment successfully. Please queue at the marked line once you are done, show the number card and collect your payment in cash.

E Personality Beliefs Questionnaire

The personality beliefs questionnaire used in the study was adapted from [22] and is presented below:
Please pick an option next to each statement to indicate the extent to which you agree or disagree with the statement **regarding the other player**.
For each of the below statements the subject could pick any one of five options - Disagree strongly, Disagree a little, Neither agree nor disagree, Agree a little and Agree strongly

1. The other player is reserved.
2. The other player is generally trusting.
3. The other player tends to be lazy.
4. The other player is relaxed, handles stress well.
5. The other player has few artistic interests.
6. The other player is outgoing, sociable.
7. The other player tends to find fault with others.
8. The other player does a thorough job.
9. The other player gets nervous easily.
10. The other player has an active imagination.
11. The other player is considerate and kind to almost everyone.

References

1. Proto E, Rustichini A, Sofianos A. Intelligence, Personality, and Gains from Cooperation in Repeated Interactions. Journal of Political Economy. 2019;127(3):1351–1390. doi:10.1086/701355.

2. Proto E, Rustichini A. Cooperation and Personality. The Warwick Economics Research Paper Series (TWERPS). 2014;(1045).

3. Rustichini A, DeYoung CG, Anderson JE, Burks SV. Toward the integration of personality theory and decision theory in explaining economic behavior: An experimental investigation. Journal of Behavioral and Experimental Economics. 2016;64:122–137. doi:10.1016/j.socec.2016.04.019.

4. Johnson MK, Rustichini A, MacDonald AW. Suspicious personality predicts behavior on a social decision-making task. Personality and Individual Differences. 2009;47(1):30–35. doi:10.1016/j.paid.2009.01.050.

5. Hirsh JB, Peterson JB. Extraversion, neuroticism, and the prisoner's dilemma. Personality and Individual Differences. 2009;46(2):254–256. doi:10.1016/j.paid.2008.10.006.

6. Arad A, Rubinstein A. The 11-20 Money Request Game: A Level-k Reasoning Study. American Economic Review. 2012;102(7):3561–73. doi:10.1257/aer.102.7.3561.

7. Eaton LG, Funder DC. The creation and consequences of the social world: an interactional analysis of extraversion. European Journal of Personality. 2003;17(5):375–395. doi:10.1002/per.477.

8. Thorne A. The press of personality: A study of conversations between introverts and extraverts. Journal of Personality and Social Psychology. 1987;53(4):718–726. doi:10.1037/0022-3514.53.4.718.

9. Tamir DI, Mitchell JP. Anchoring and adjustment during social inferences. Journal of Experimental Psychology: General. 2013;142(1):151–162. doi:10.1037/a0028232.

10. Thomas K, DeScioli P, Sultan Haque O, Pinker S. The Psychology of Coordination and Common Knowledge. Journal of Personality and Social Psychology. 2014;107(4):657–676. doi:10.1037/a0037037.

11. Carlo G, Okun MA, Knight GP, de Guzman MRT. The interplay of traits and motives on volunteering: Agreeableness, extraversion and prosocial value motivation. Personality and Individual Differences. 2005;38(6):1293–1305. doi:10.1016/j.paid.2004.08.012.

12. Burke DM, Hall M. Personality Characteristics of Volunteers in a Companion for Children Program. Psychological Reports. 1986;59(2):819–825. doi:10.2466/pr0.1986.59.2.819.

13. Ross SR, Rausch MK, Canada KE. Competition and cooperation in the five-factor model: Individual differences in achievement orientation. The Journal of psychology. 2003;137(4):323–37. doi:10.1080/00223980309600617.

14. Koole SL, Jager W, van den Berg AE, Vlek CA, Hofstee WK. On the Social Nature of Personality: Effects of Extraversion, Agreeableness, and Feedback About Collective Resource Use on Cooperation in a Resource Dilemma. Personality and Social Psychology Bulletin. 2001;27(3):289–301. doi:10.1177/0146167201273003.

15. Costa PT, McCrae RR. Influence of extraversion and neuroticism on subjective well-being: Happy and unhappy people. Journal of Personality and Social Psychology. 1980;38:668–678. doi:10.1037//0022-3514.38.4.668.

16. Lai DWL, Qin N. Extraversion personality, perceived health and activity participation among community-dwelling aging adults in Hong Kong. PloS one. 2018;13(12):1–13. doi:10.1371/journal.pone.0209154.

17. Tov W, Nai ZL, Lee HW. Extraversion and Agreeableness: Divergent Routes to Daily Satisfaction With Social Relationships. Journal of Personality. 2016;84(1):121–134. doi:10.1111/jopy.12146.

18. King DD, Ott-Holland CJ, Ryan AM, Huang JL, Wadlington PL, Elizondo F. Personality Homogeneity in Organizations and Occupations: Considering Similarity Sources. Journal of Business and Psychology. 2017;32(6):641–653. doi:10.1007/s10869-016-9459-4.

19. Warriner AB, Kuperman V, Brysbaert M. Norms of valence, arousal, and dominance for 13,915 English lemmas. Behavior Research Methods. 2013;45 VN - r(4):1191–1207. doi:10.3758/s13428-012-0314-x.

20. Mehl MR, Gosling SD, Pennebaker JW. Personality in its natural habitat: Manifestations and implicit folk theories of personality in daily life. Journal of Personality and Social Psychology. 2006;90(5):862–877. doi:10.1037/0022-3514.90.5.862.

21. Bursztyn L, Ferman B, Fiorin S, Kanz M, Rao G. Status Goods: Experimental Evidence from Platinum Credit Cards. The Quarterly Journal of Economics. 2017;133(3):1561–1595. doi:10.1093/qje/qjx048.

22. Rammstedt B, John OP. Measuring personality in one minute or less: A 10-item short version of the Big Five Inventory in English and German. Journal of Research in Personality. 2007;41(1):203–212. doi:10.1016/j.jrp.2006.02.001.
