# Supplementary figures and images for: The role of personality beliefs and “small talk” in strategic behaviour
Source: PLoS One. 2022 Sep 2;17(9):e0269523. doi: 10.1371/journal.pone.0269523 (PMC9438804; doi:10.1371/journal.pone.0269523)

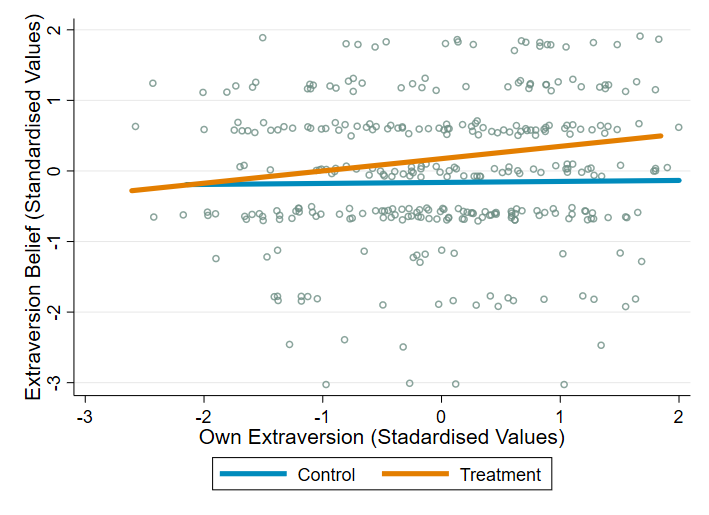

Supplement: S2 File — (ZIP) [file pone.0269523.s002.zip › SmallTalk_Figures/beliefs_interactionplot.png]

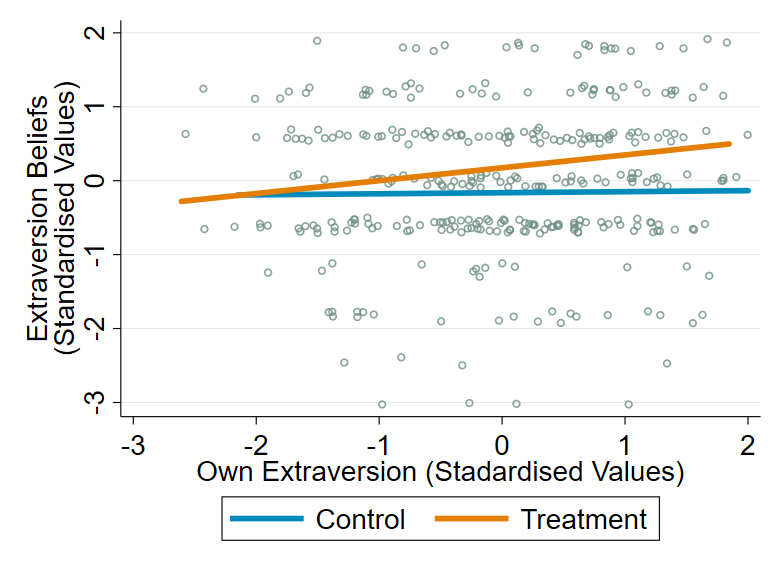

Supplement: S2 File — (ZIP) [file pone.0269523.s002.zip › SmallTalk_Figures/beliefs_projection.png]

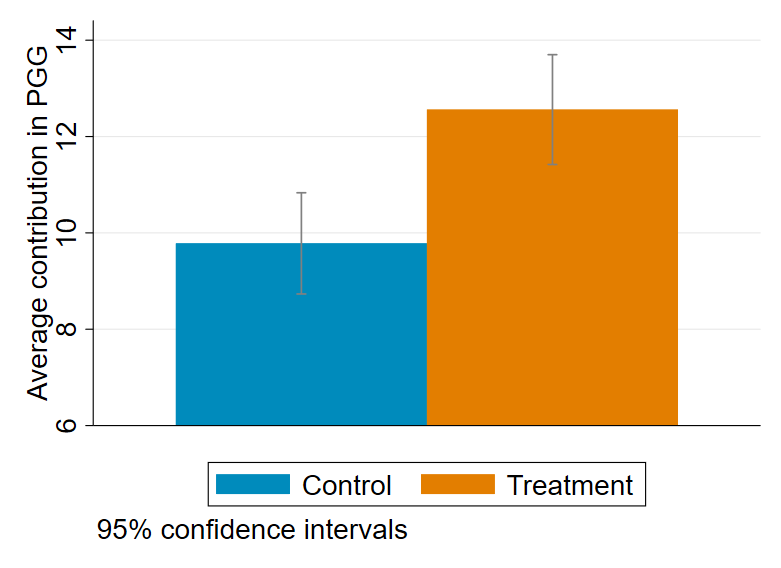

Supplement: S2 File — (ZIP) [file pone.0269523.s002.zip › SmallTalk_Figures/contribution.png]

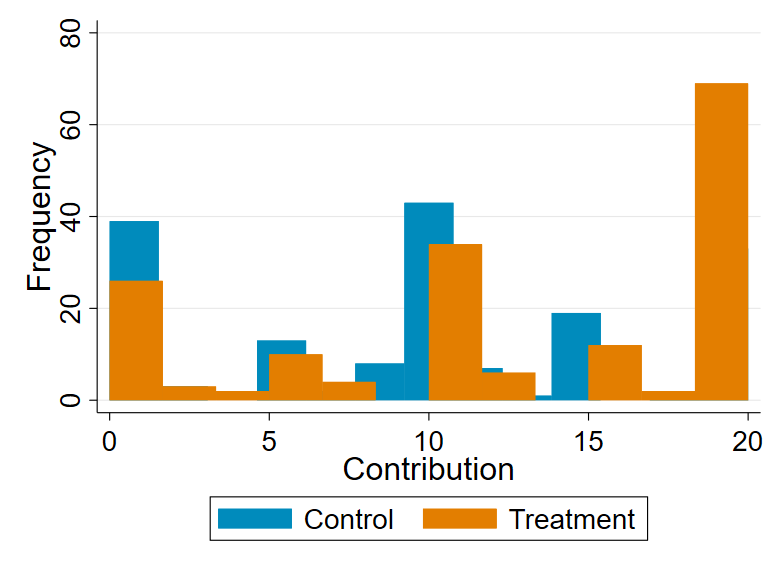

Supplement: S2 File — (ZIP) [file pone.0269523.s002.zip › SmallTalk_Figures/contributiondist.png]

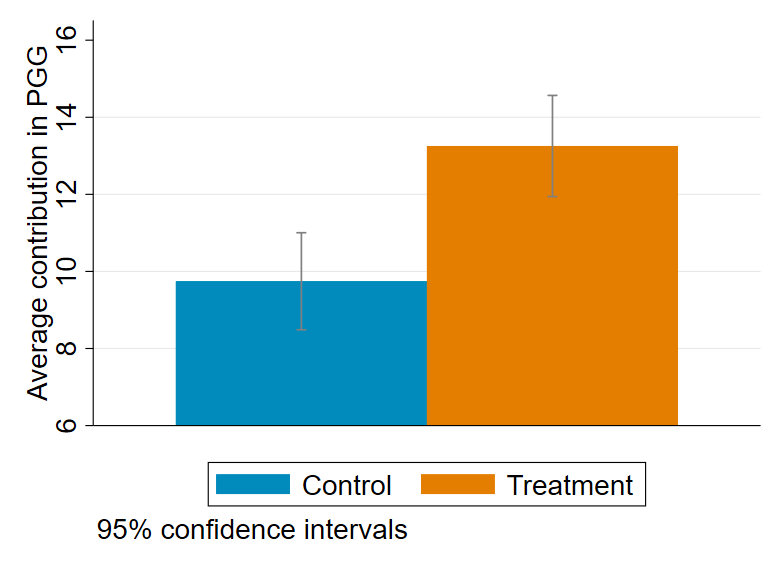

Supplement: S2 File — (ZIP) [file pone.0269523.s002.zip › SmallTalk_Figures/ContributionOrder1.png]

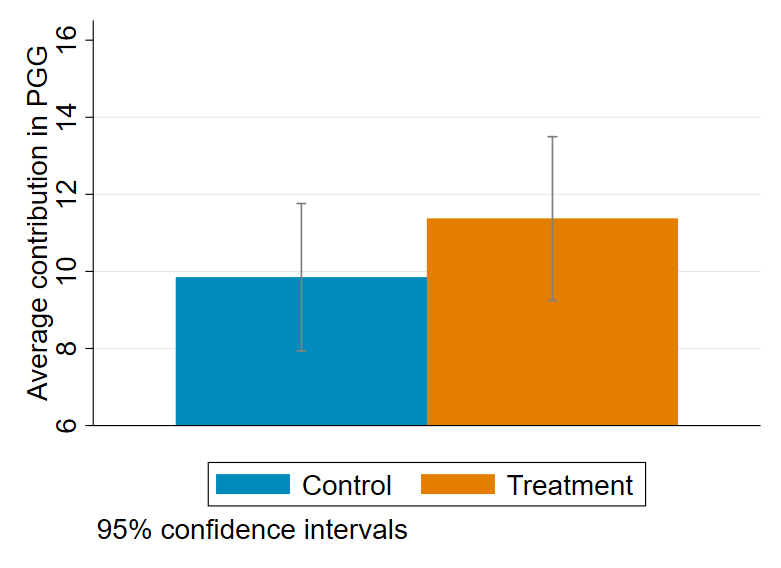

Supplement: S2 File — (ZIP) [file pone.0269523.s002.zip › SmallTalk_Figures/ContributionOrder2.png]

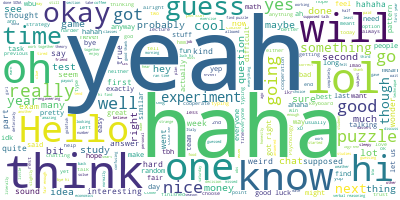

Supplement: S2 File — (ZIP) [file pone.0269523.s002.zip › SmallTalk_Figures/imageALL.png]

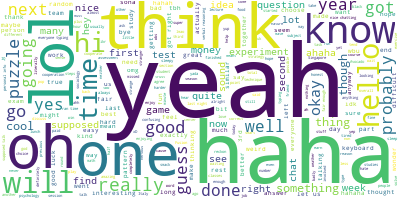

Supplement: S2 File — (ZIP) [file pone.0269523.s002.zip › SmallTalk_Figures/imageHE.png]

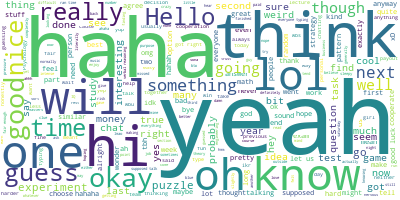

Supplement: S2 File — (ZIP) [file pone.0269523.s002.zip › SmallTalk_Figures/imageHN.png]

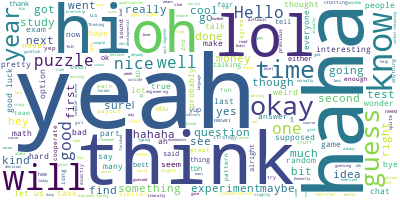

Supplement: S2 File — (ZIP) [file pone.0269523.s002.zip › SmallTalk_Figures/imageLE.png]

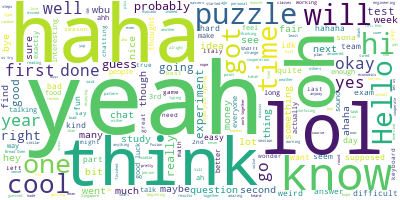

Supplement: S2 File — (ZIP) [file pone.0269523.s002.zip › SmallTalk_Figures/imageLN.png]

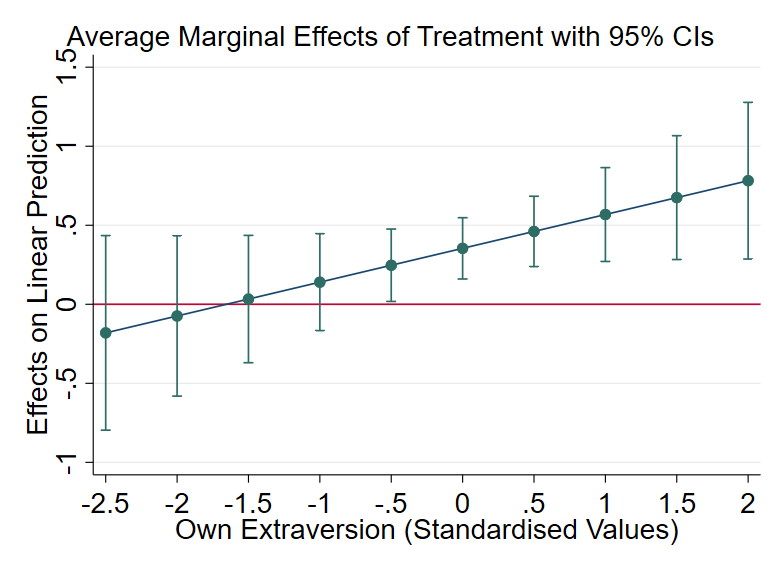

Supplement: S2 File — (ZIP) [file pone.0269523.s002.zip › SmallTalk_Figures/interaction_extraversion.png]

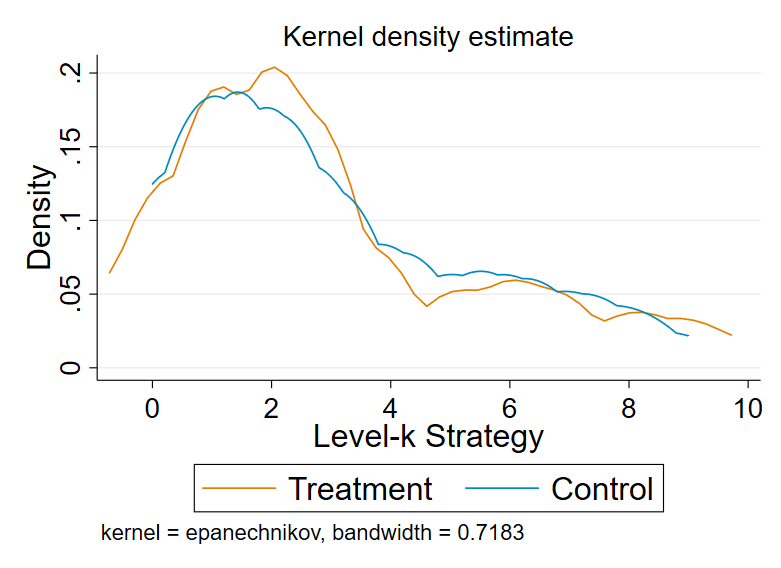

Supplement: S2 File — (ZIP) [file pone.0269523.s002.zip › SmallTalk_Figures/kernel_level.png]

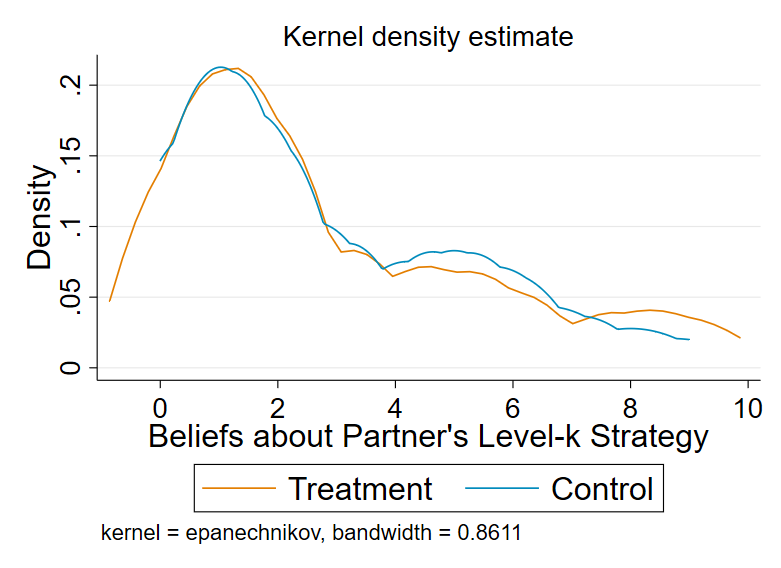

Supplement: S2 File — (ZIP) [file pone.0269523.s002.zip › SmallTalk_Figures/kernel_levelbelief.png]

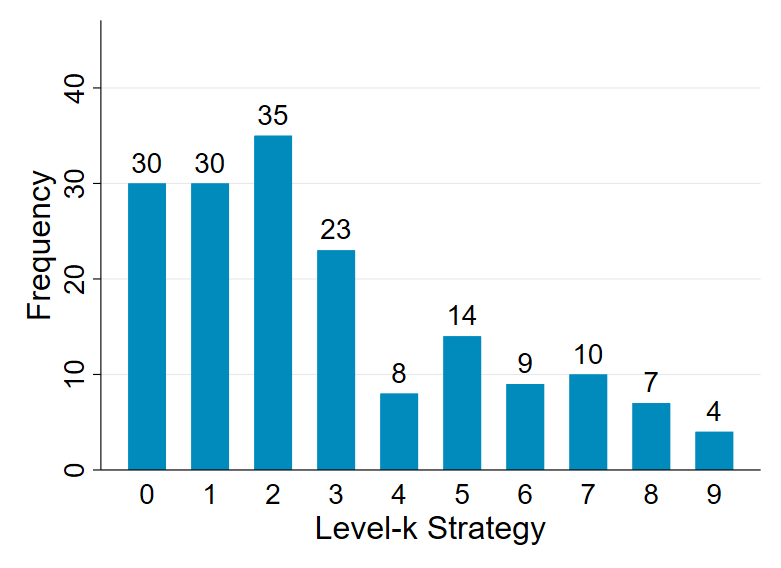

Supplement: S2 File — (ZIP) [file pone.0269523.s002.zip › SmallTalk_Figures/level_control.png]

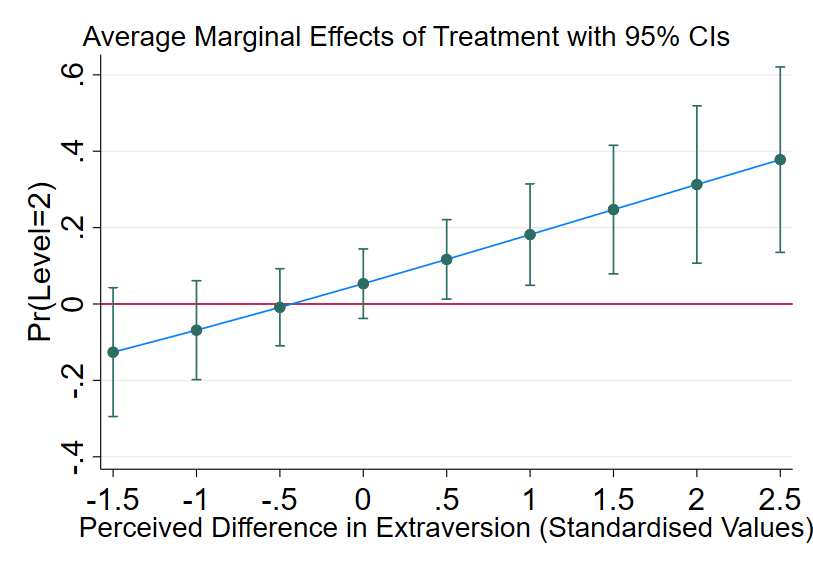

Supplement: S2 File — (ZIP) [file pone.0269523.s002.zip › SmallTalk_Figures/level_prob.png]

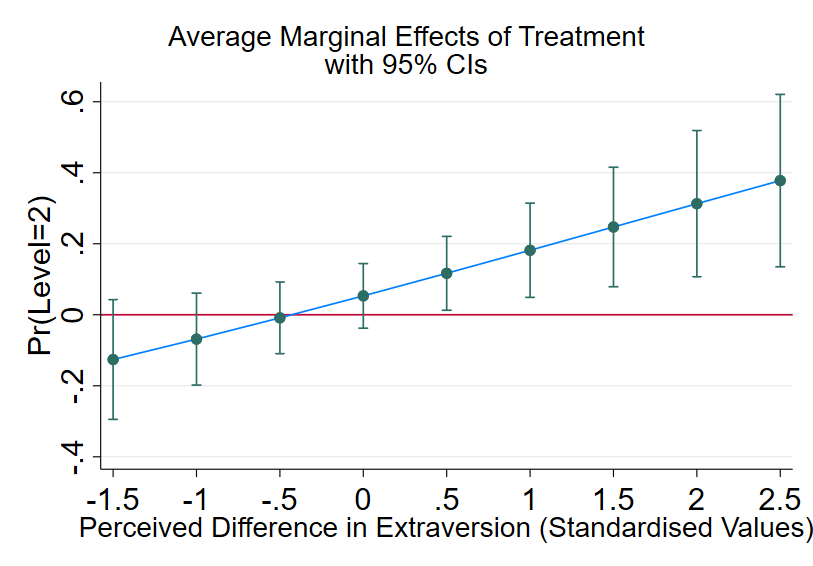

Supplement: S2 File — (ZIP) [file pone.0269523.s002.zip › SmallTalk_Figures/level_prob2.png]

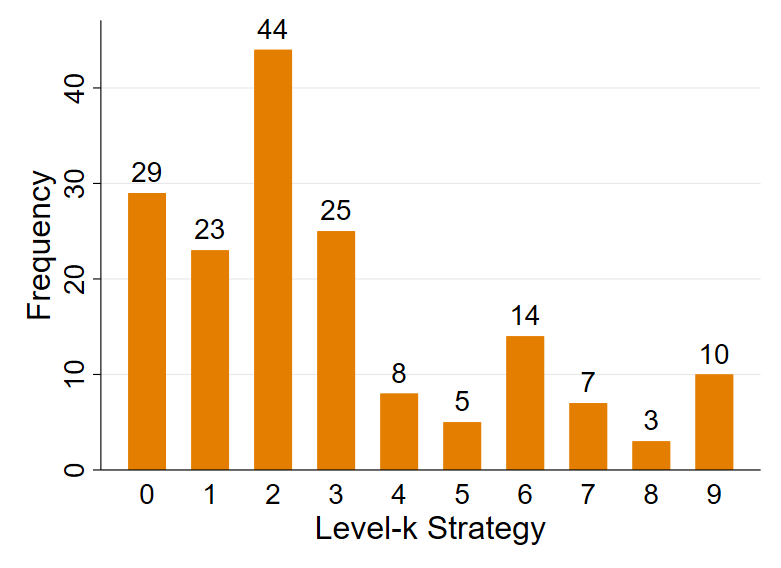

Supplement: S2 File — (ZIP) [file pone.0269523.s002.zip › SmallTalk_Figures/level_treatment.png]

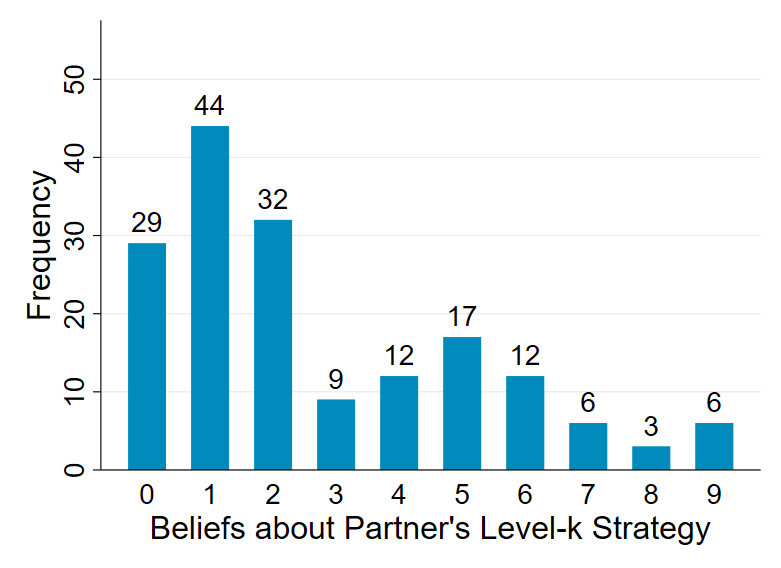

Supplement: S2 File — (ZIP) [file pone.0269523.s002.zip › SmallTalk_Figures/levelbelief_control.png]

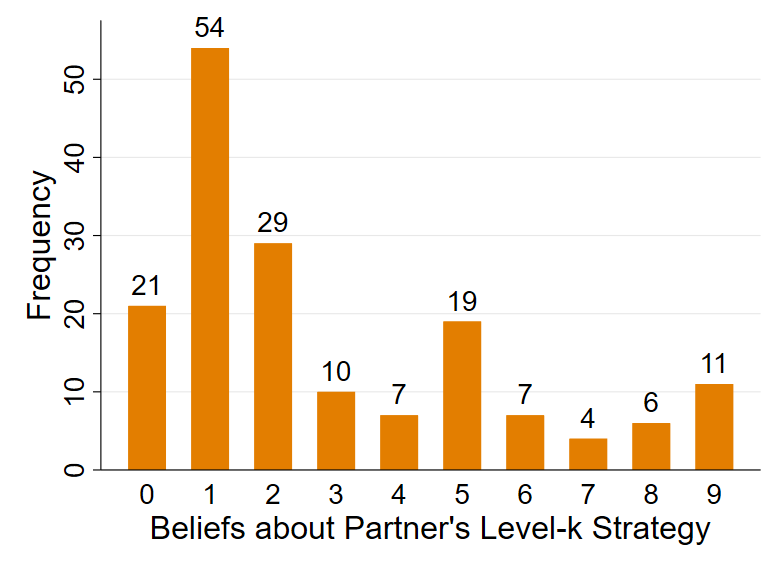

Supplement: S2 File — (ZIP) [file pone.0269523.s002.zip › SmallTalk_Figures/levelbelief_treatment.png]

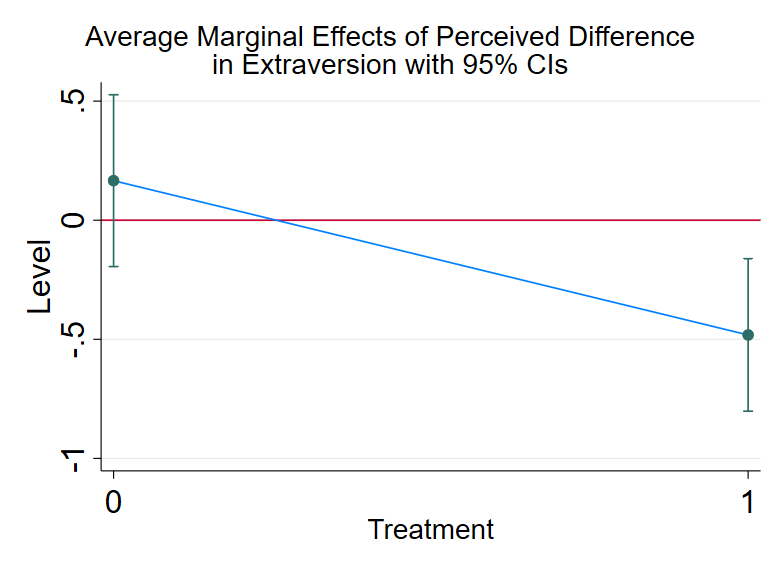

Supplement: S2 File — (ZIP) [file pone.0269523.s002.zip › SmallTalk_Figures/levelk_treatment.png]

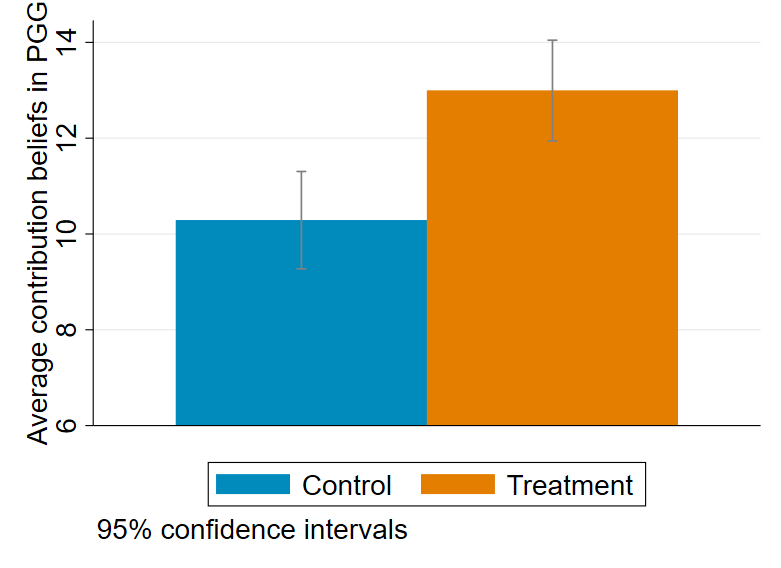

Supplement: S2 File — (ZIP) [file pone.0269523.s002.zip › SmallTalk_Figures/PGGbelief.png]

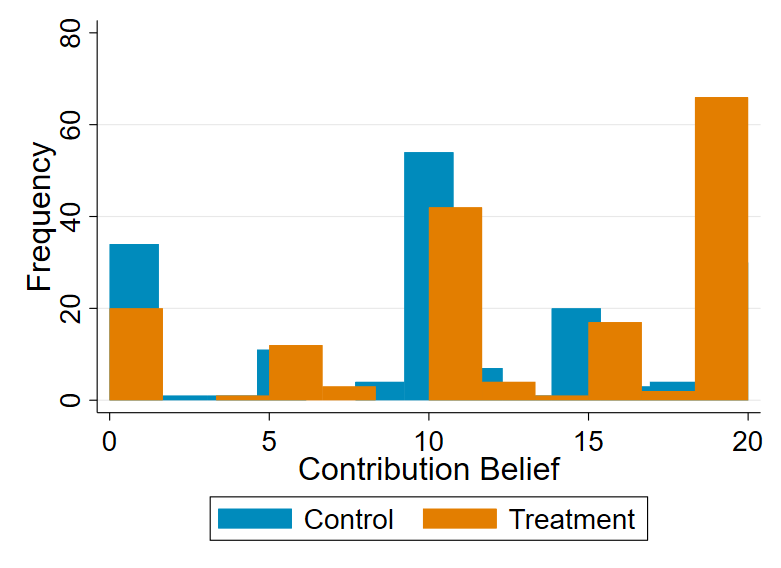

Supplement: S2 File — (ZIP) [file pone.0269523.s002.zip › SmallTalk_Figures/PGGbeliefdist.png]

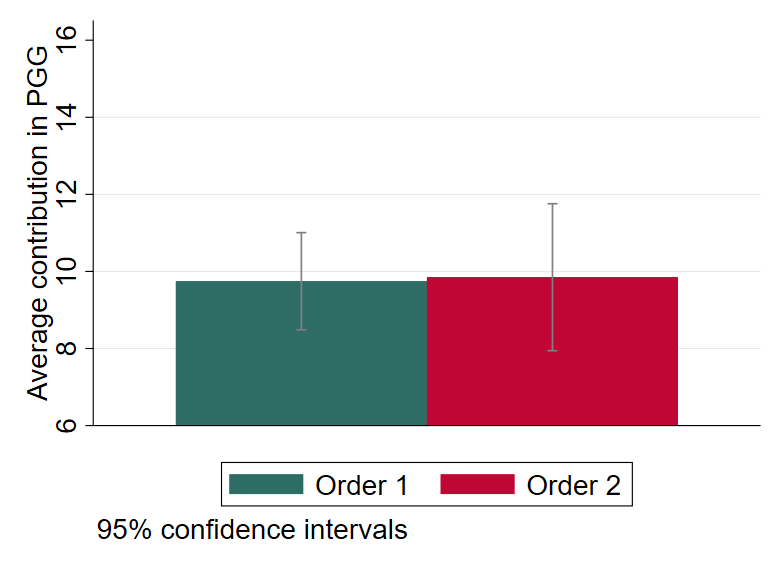

Supplement: S2 File — (ZIP) [file pone.0269523.s002.zip › SmallTalk_Figures/seqControl.png]

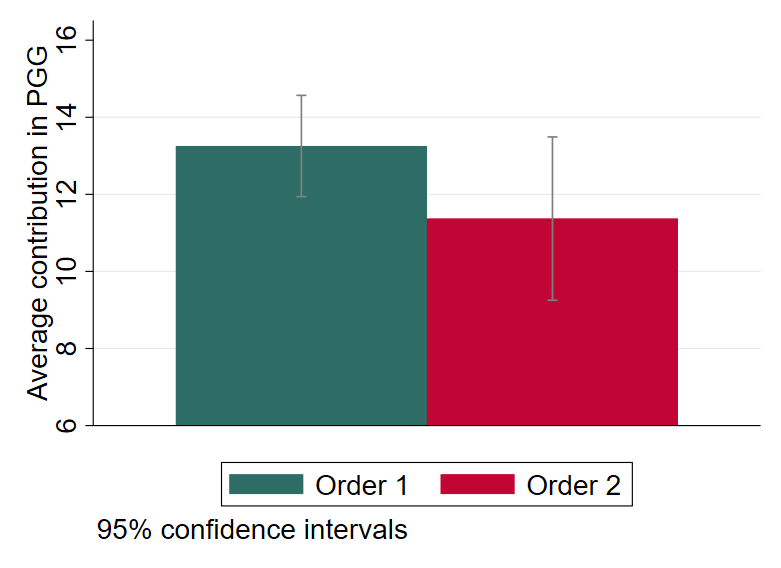

Supplement: S2 File — (ZIP) [file pone.0269523.s002.zip › SmallTalk_Figures/seqTreatment.png]

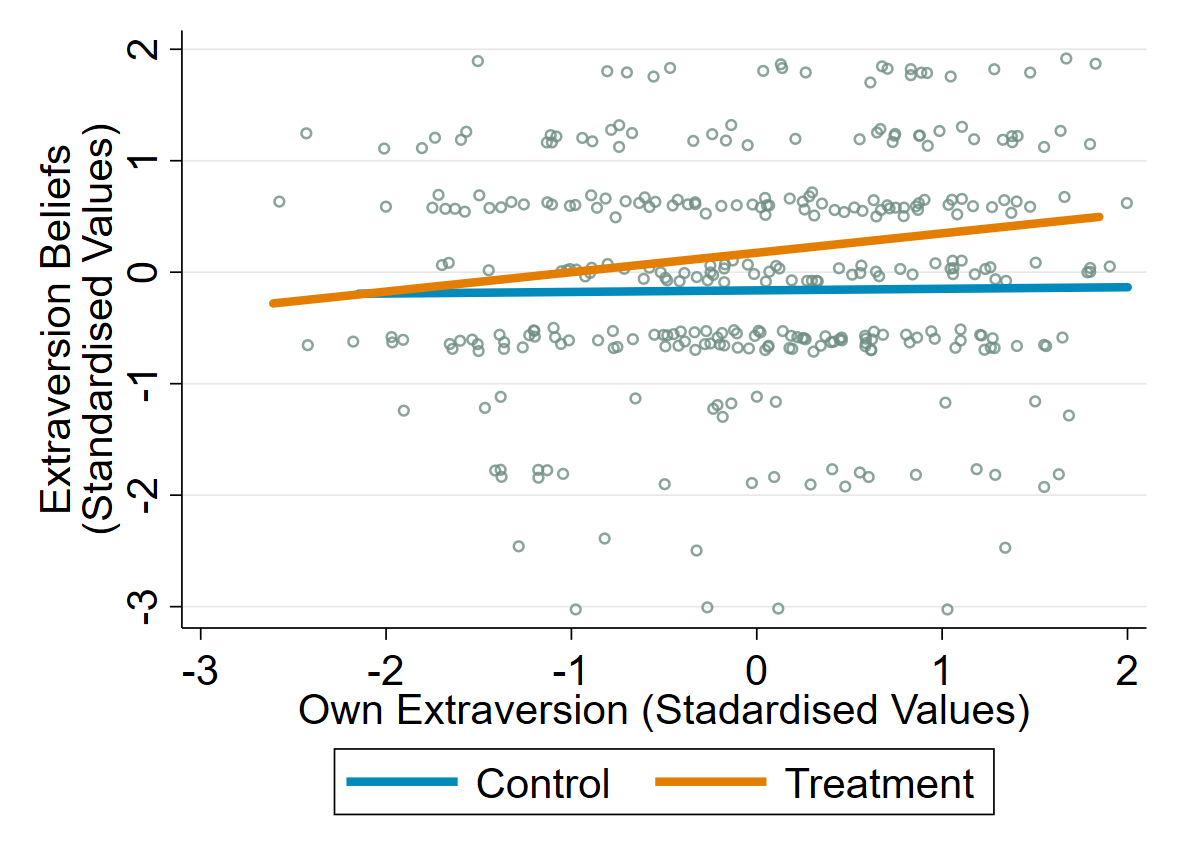

Supplement: S2 File — (ZIP) [file pone.0269523.s002.zip › Main_paper_tif_figures/Fig1A.tif]

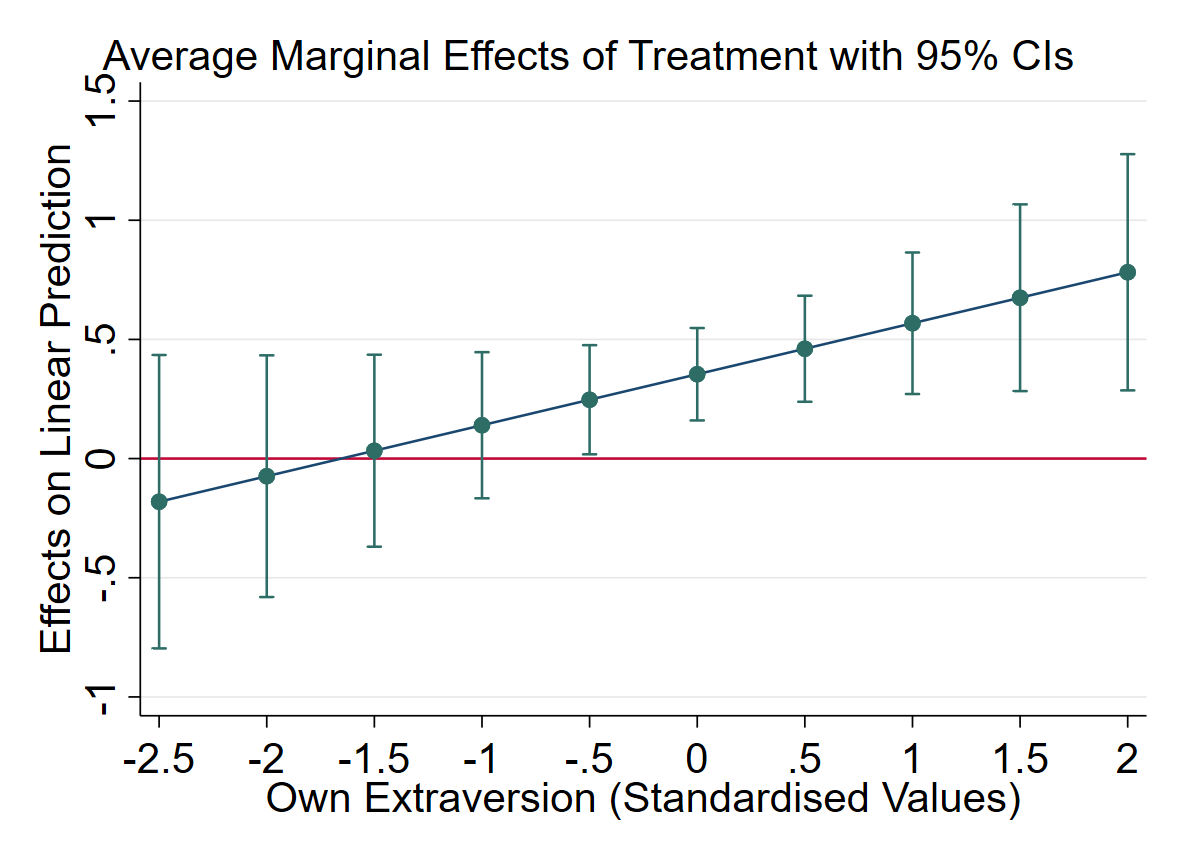

Supplement: S2 File — (ZIP) [file pone.0269523.s002.zip › Main_paper_tif_figures/Fig1B.tif]

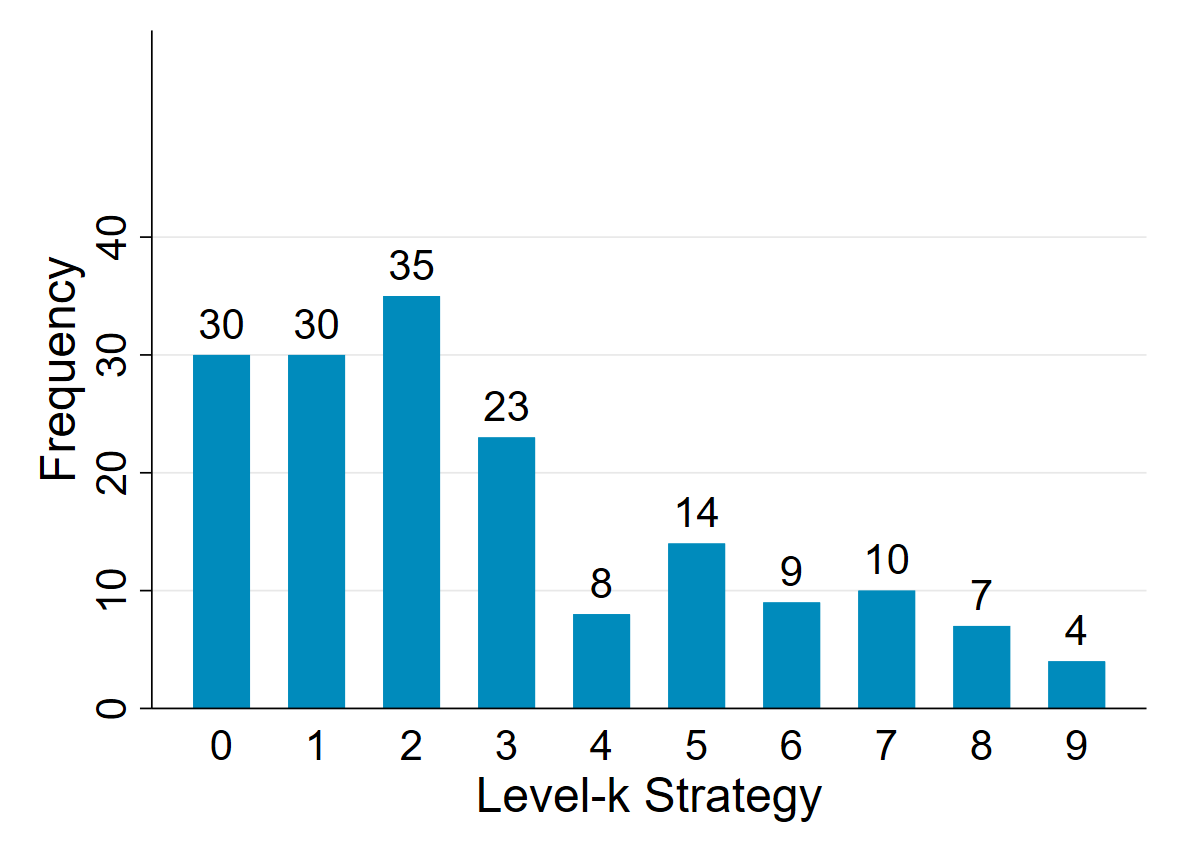

Supplement: S2 File — (ZIP) [file pone.0269523.s002.zip › Main_paper_tif_figures/Fig2A.tif]

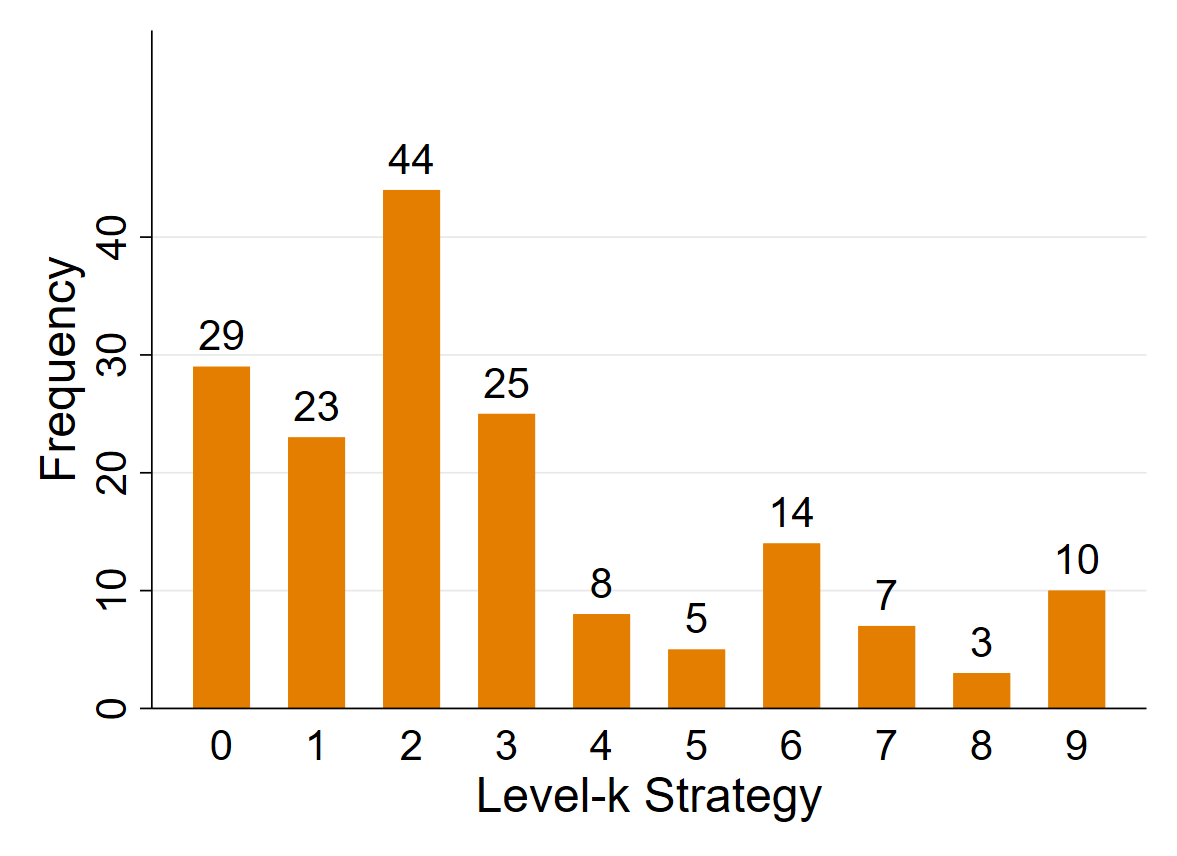

Supplement: S2 File — (ZIP) [file pone.0269523.s002.zip › Main_paper_tif_figures/Fig2B.tif]

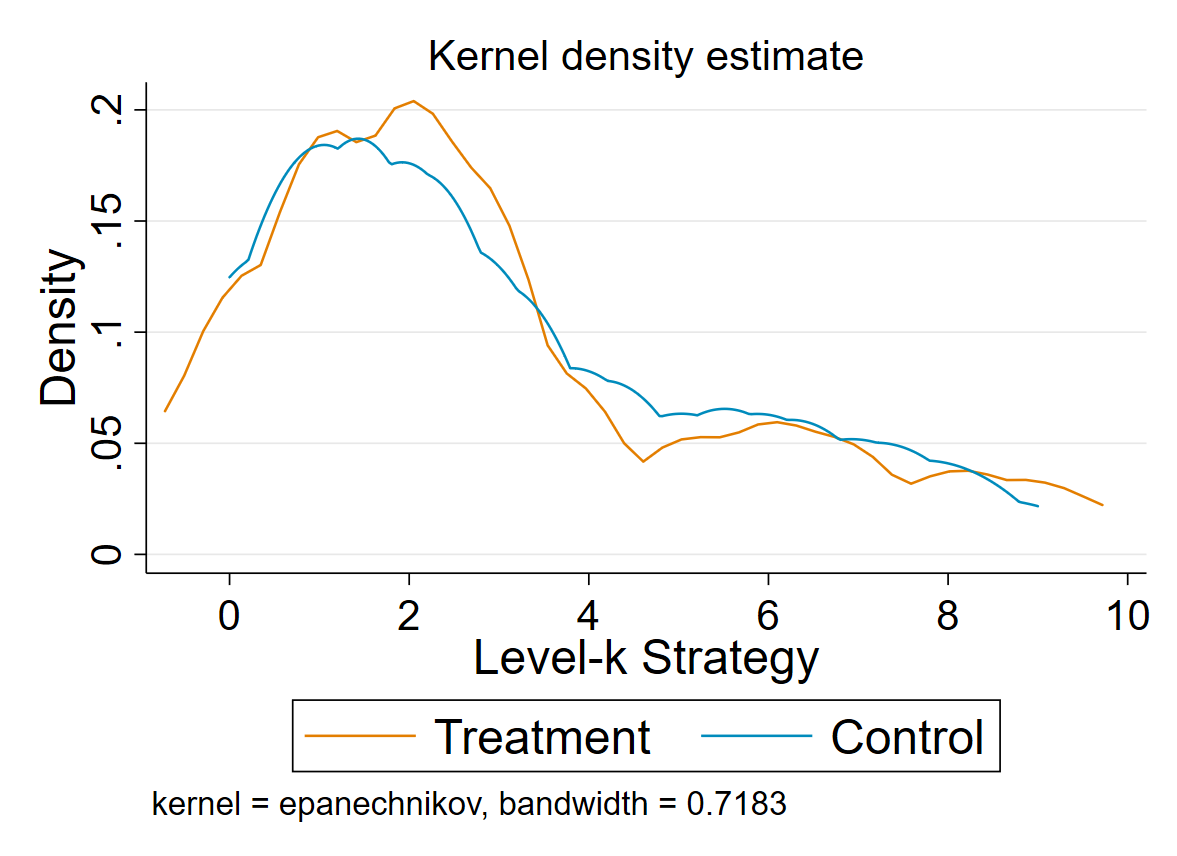

Supplement: S2 File — (ZIP) [file pone.0269523.s002.zip › Main_paper_tif_figures/Fig2C.tif]

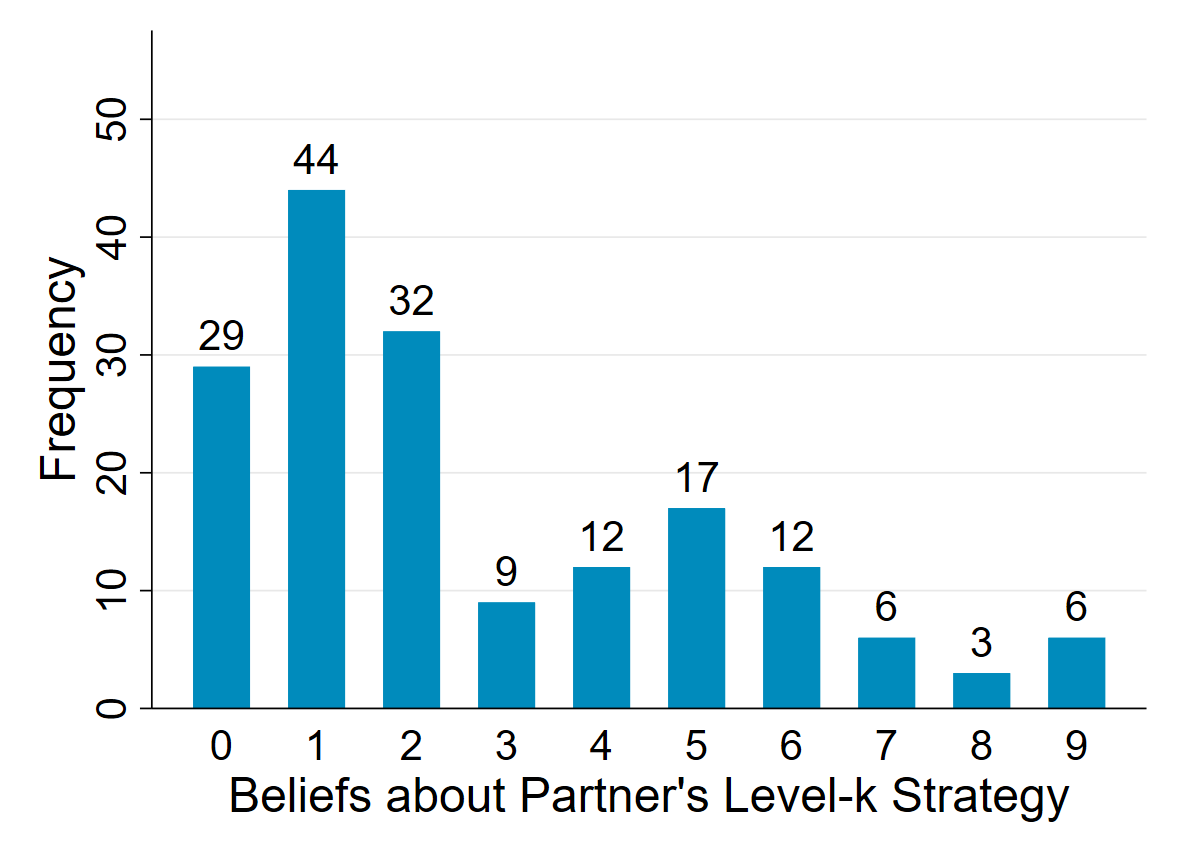

Supplement: S2 File — (ZIP) [file pone.0269523.s002.zip › Main_paper_tif_figures/Fig3A.tif]

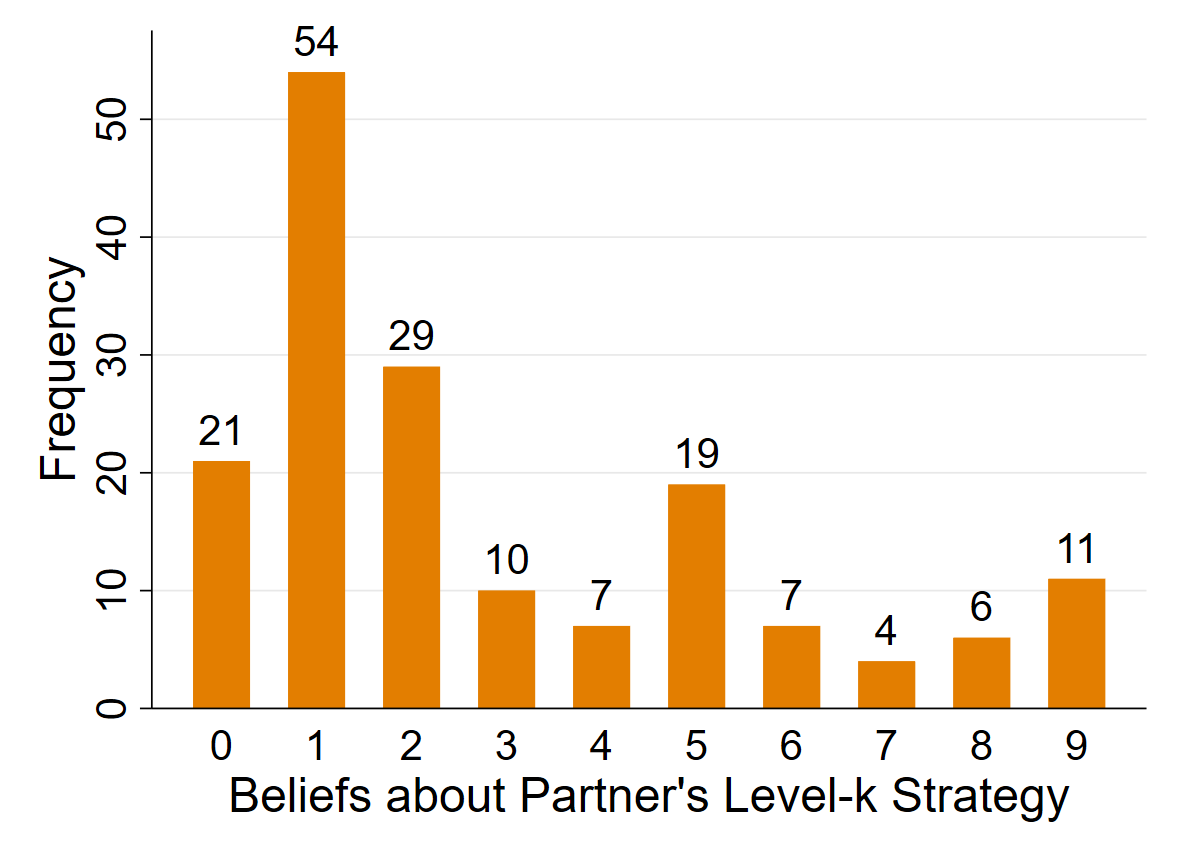

Supplement: S2 File — (ZIP) [file pone.0269523.s002.zip › Main_paper_tif_figures/Fig3B.tif]

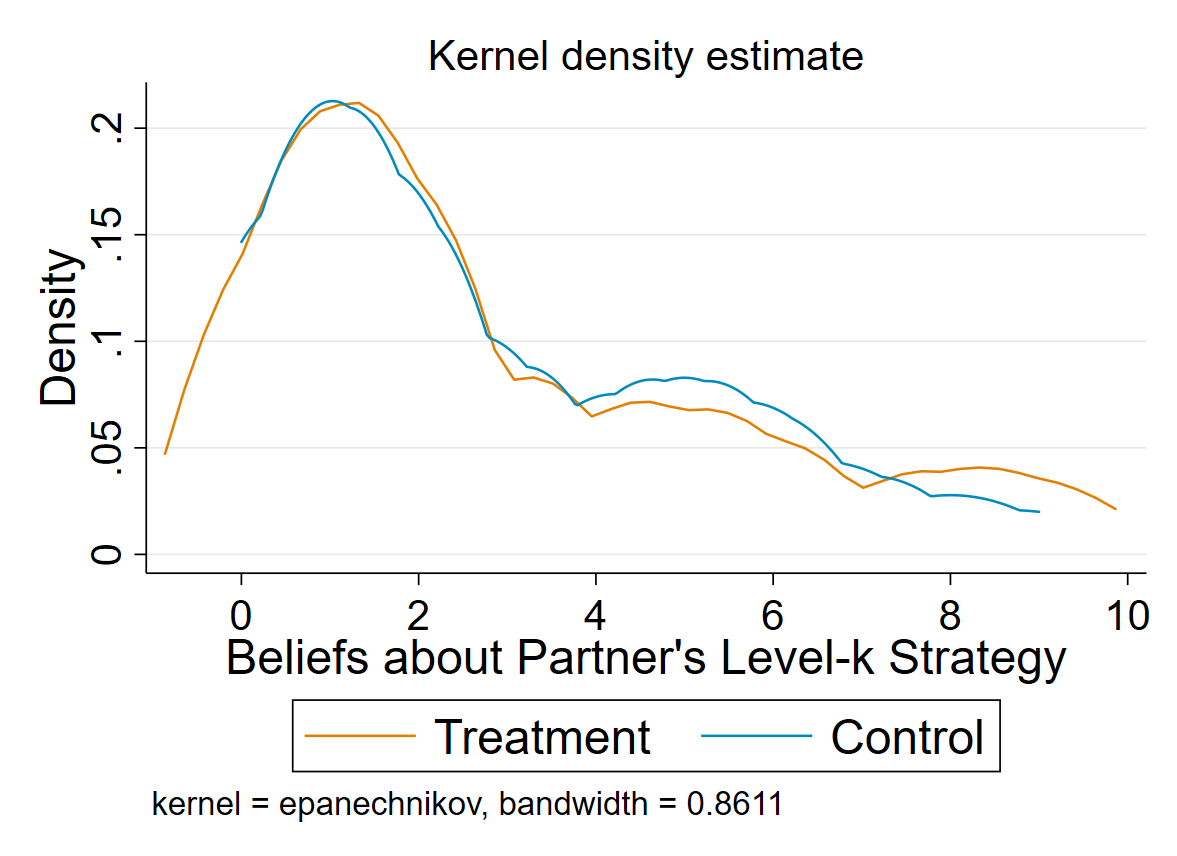

Supplement: S2 File — (ZIP) [file pone.0269523.s002.zip › Main_paper_tif_figures/Fig3C.tif]

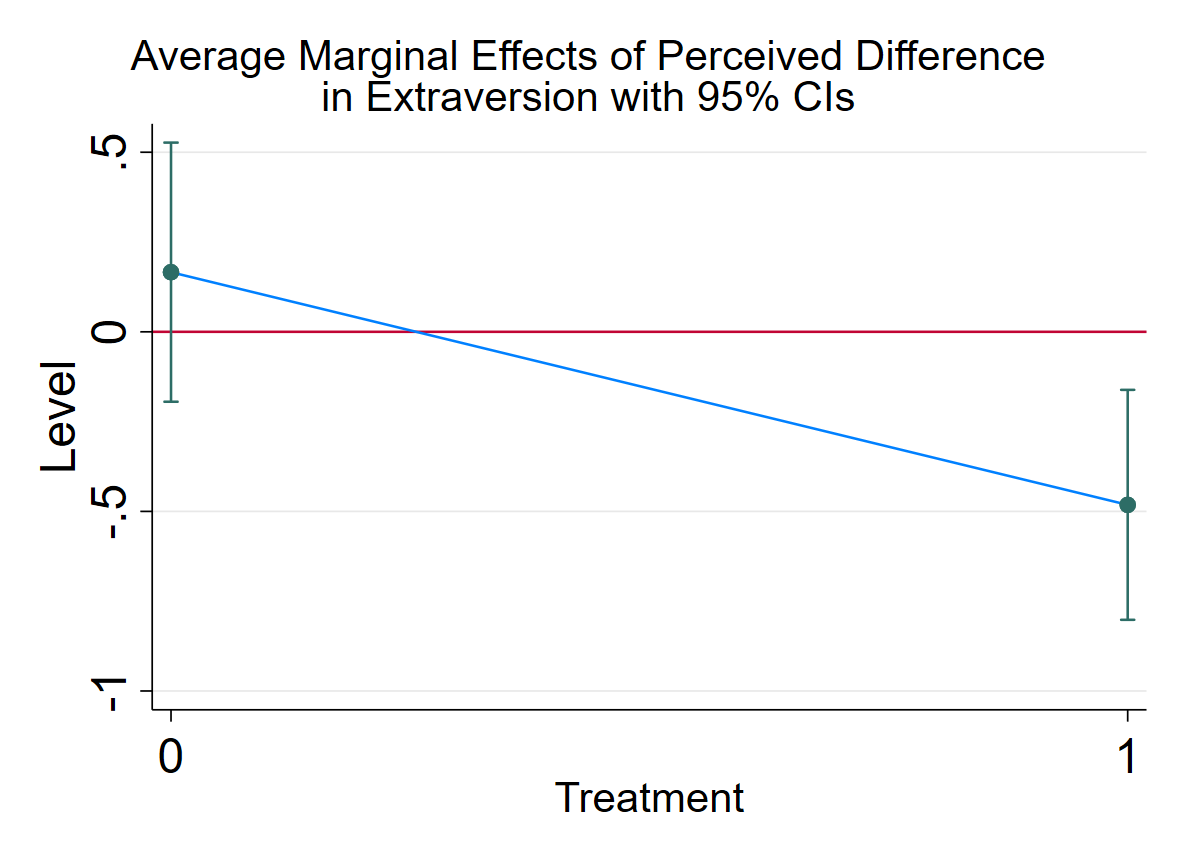

Supplement: S2 File — (ZIP) [file pone.0269523.s002.zip › Main_paper_tif_figures/Fig4A.tif]

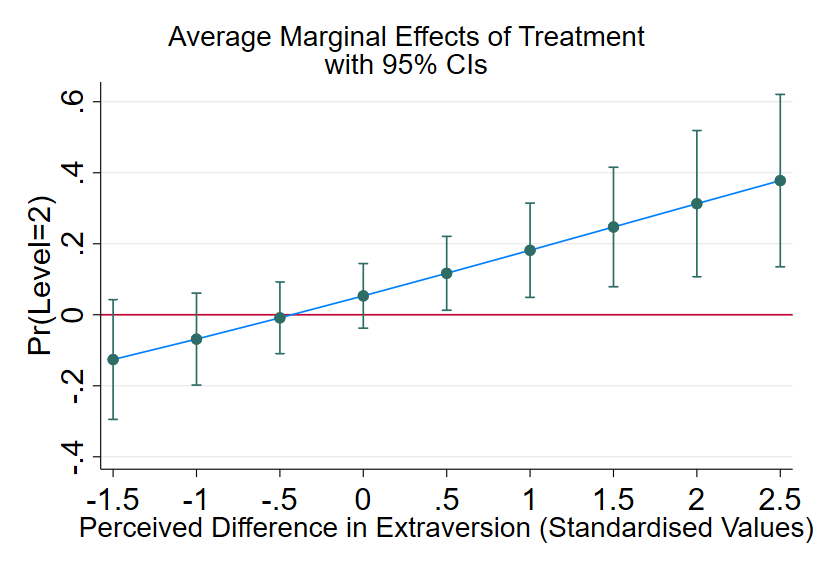

Supplement: S2 File — (ZIP) [file pone.0269523.s002.zip › Main_paper_tif_figures/Fig4B.tif]

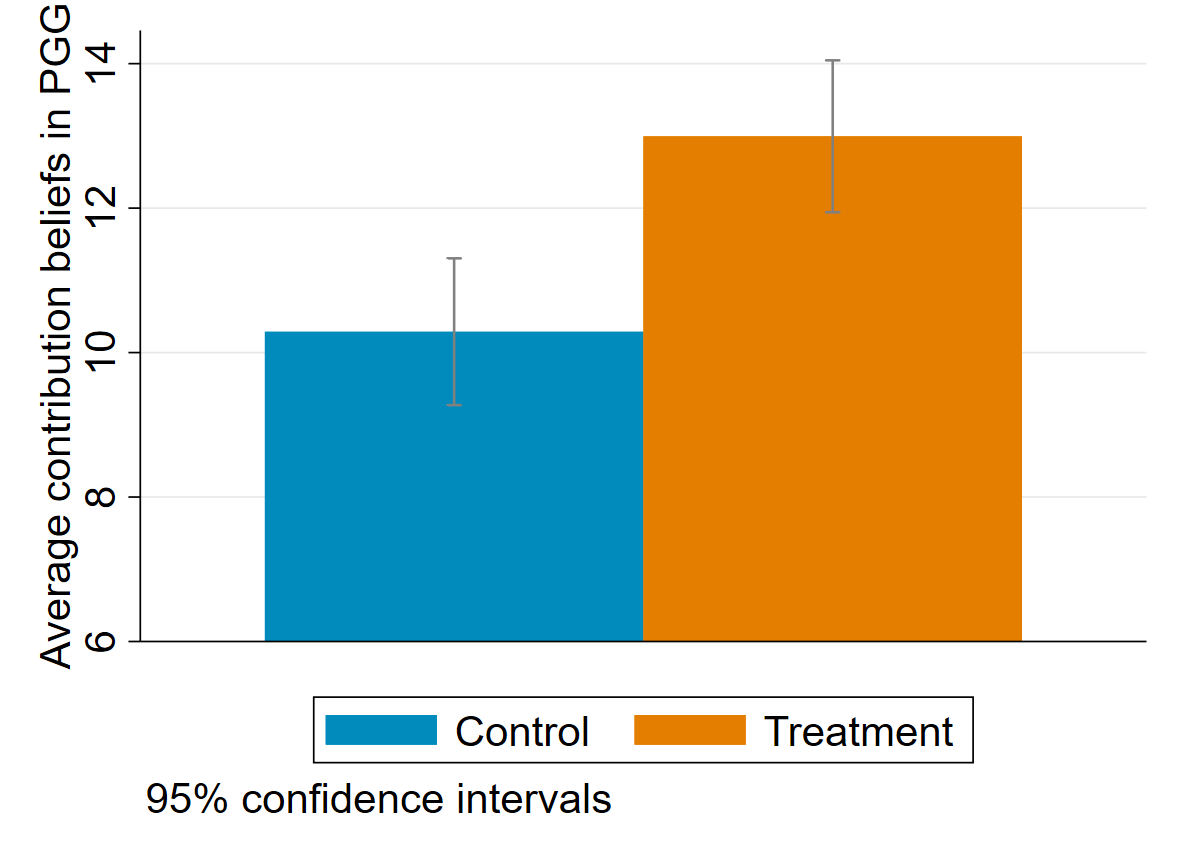

Supplement: S2 File — (ZIP) [file pone.0269523.s002.zip › Main_paper_tif_figures/Fig5A.tif]

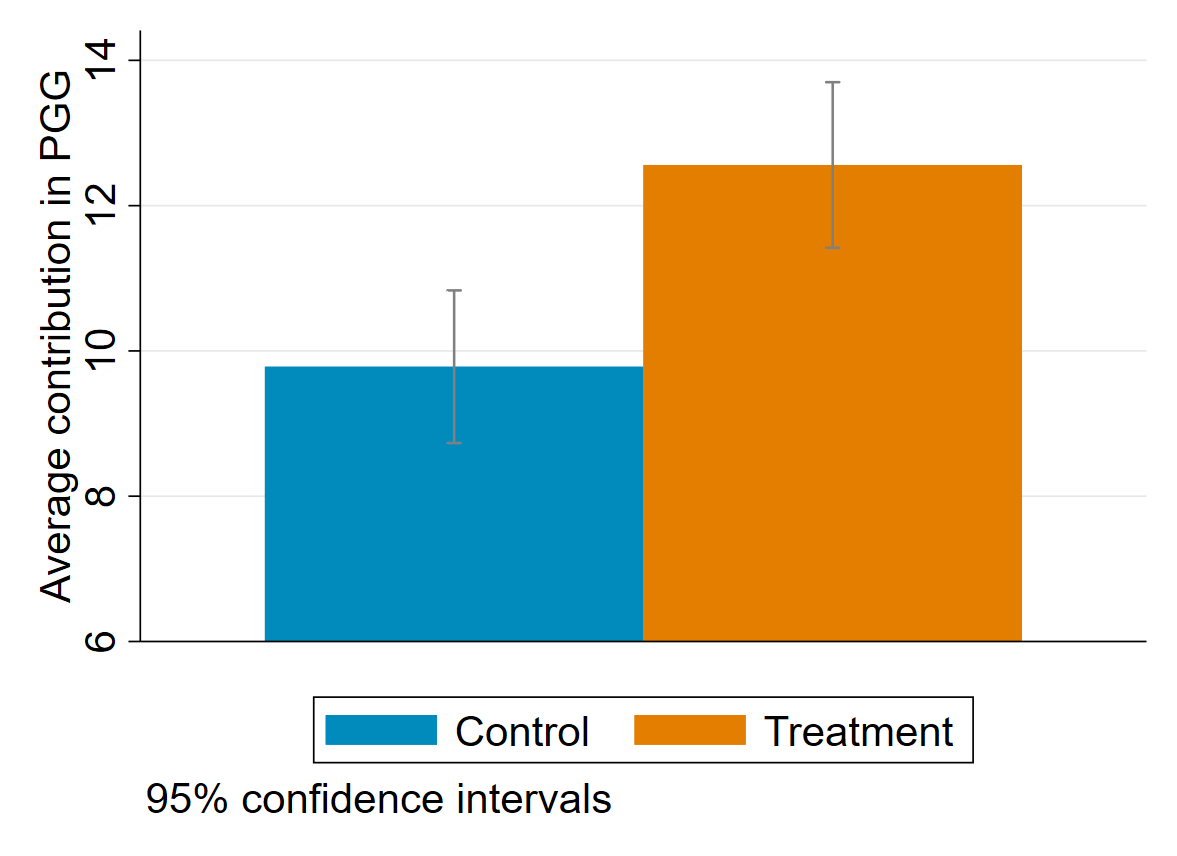

Supplement: S2 File — (ZIP) [file pone.0269523.s002.zip › Main_paper_tif_figures/Fig5B.tif]

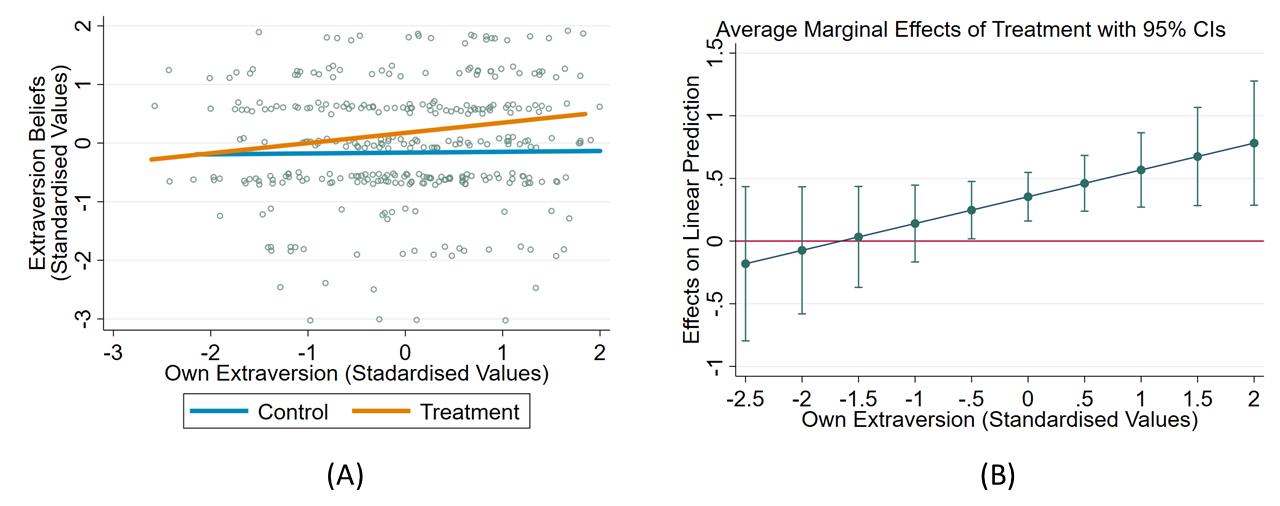

Supplement: S2 File — (ZIP) [file pone.0269523.s002.zip › Combining_multipanel_figures/Fig1.tif]

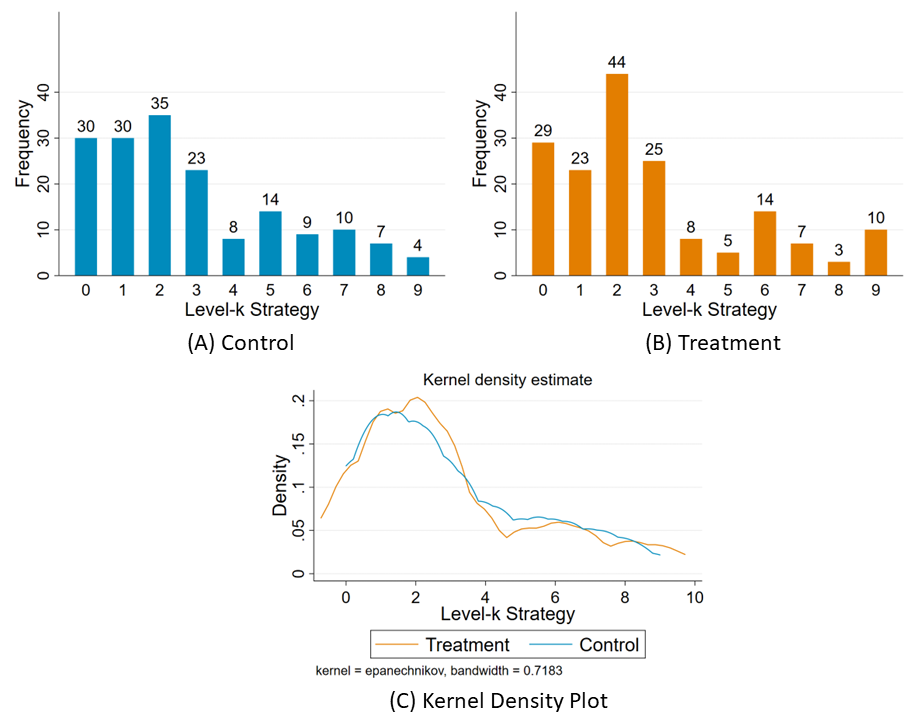

Supplement: S2 File — (ZIP) [file pone.0269523.s002.zip › Combining_multipanel_figures/Fig2.tif]

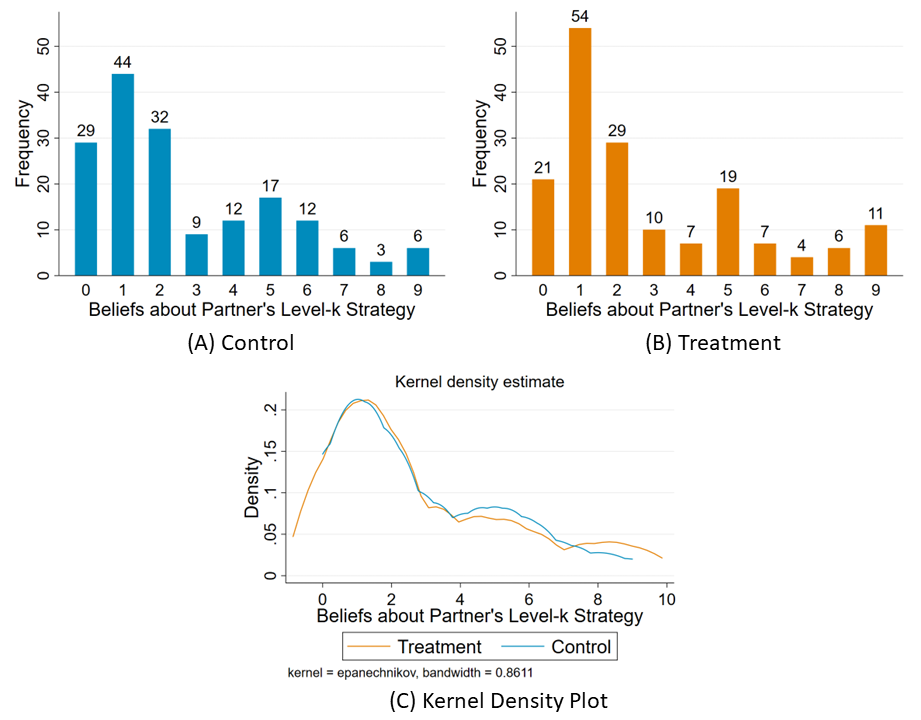

Supplement: S2 File — (ZIP) [file pone.0269523.s002.zip › Combining_multipanel_figures/Fig3.tif]

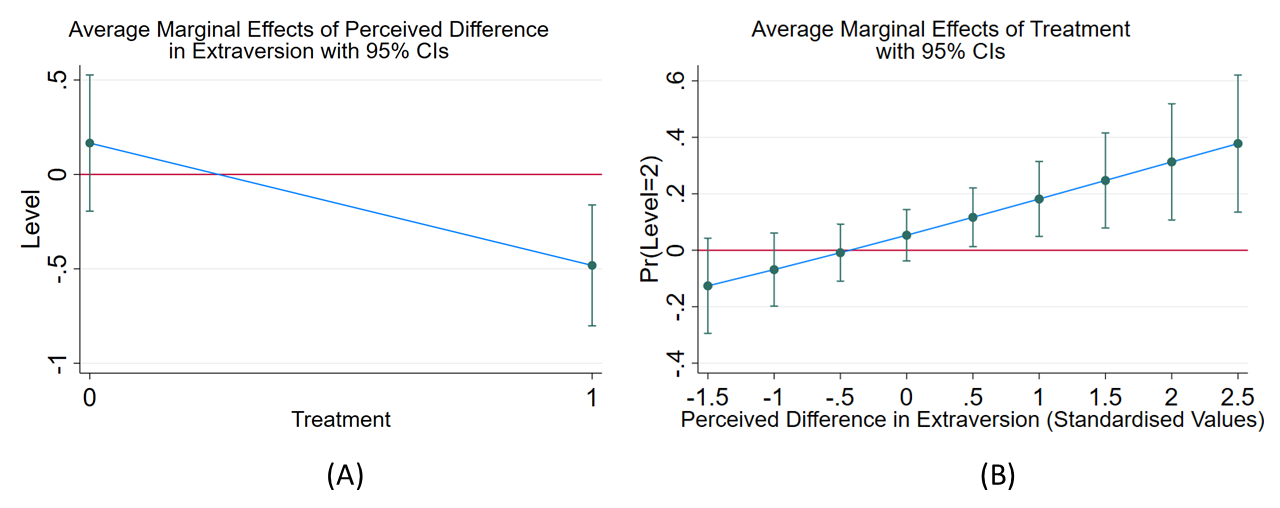

Supplement: S2 File — (ZIP) [file pone.0269523.s002.zip › Combining_multipanel_figures/Fig4.tif]

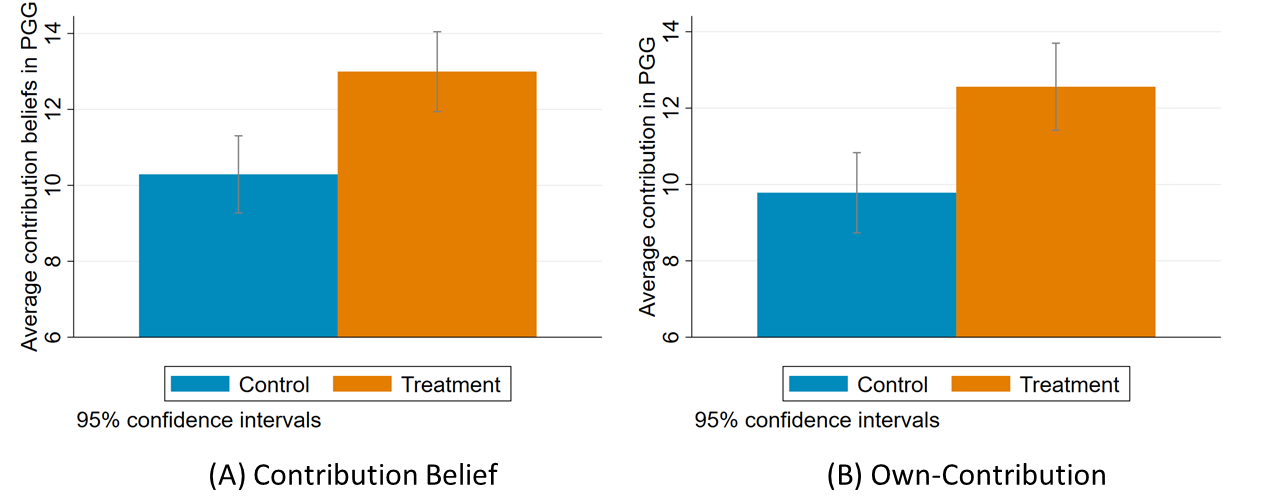

Supplement: S2 File — (ZIP) [file pone.0269523.s002.zip › Combining_multipanel_figures/Fig5.tif]
